# Arginine ADP-Ribosylation: Chemical Synthesis of Post-Translationally Modified Ubiquitin Proteins

**DOI:** 10.1021/jacs.2c06249

**Published:** 2022-11-01

**Authors:** Jim Voorneveld, Max S. Kloet, Sven Wijngaarden, Robbert Q. Kim, Angeliki Moutsiopoulou, Marnix Verdegaal, Mohit Misra, Ivan Đikić, Gijsbert A. van der Marel, Herman S. Overkleeft, Dmitri V. Filippov, Gerbrand J. van der Heden van Noort

**Affiliations:** † Bio-Organic Synthesis, Leiden Institute of Chemistry, Leiden University, Einsteinweg 55, 2333 CC Leiden, The Netherlands; ‡ Oncode Institute and Department of Cell and Chemical Biology, Leiden University Medical Centre, Einthovenweg 20, 2333 ZC Leiden, The Netherlands; § Buchmann Institute for Molecular Life Sciences, Goethe University Frankfurt, Max-von-Laue-Straße 15, 60438 Frankfurt am Main, Germany

## Abstract

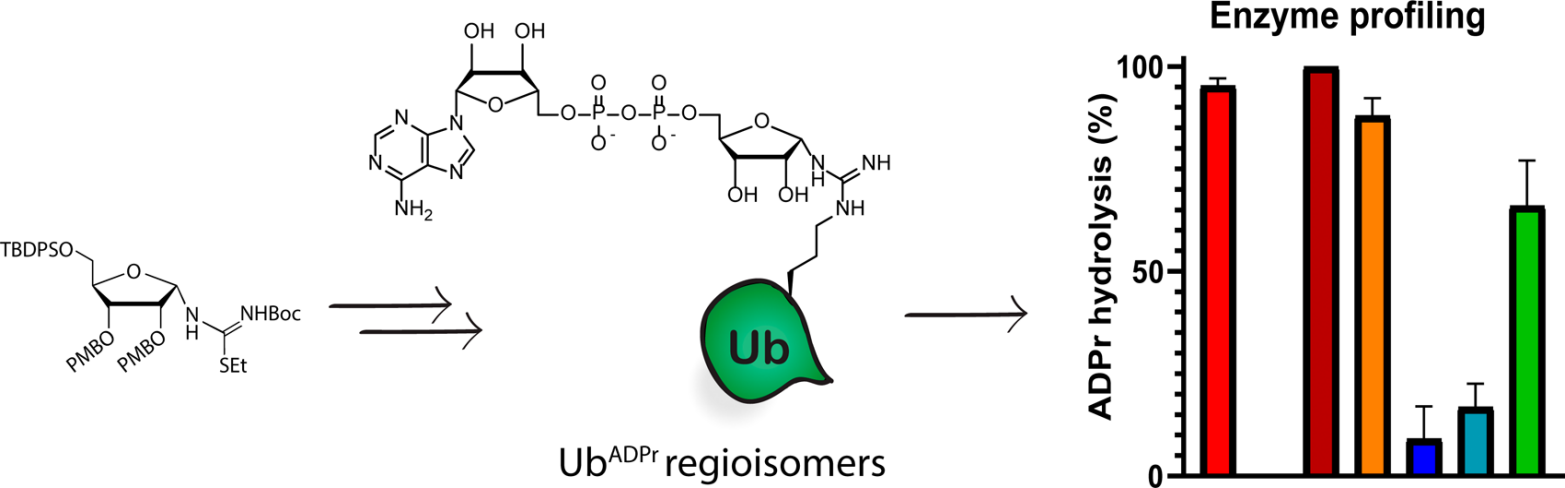

We describe the development and optimization of a methodology to prepare peptides and proteins modified on the arginine residue with an adenosine-di-phosphate-ribosyl (ADPr) group. Our method comprises reacting an ornithine containing polypeptide on-resin with an α-linked anomeric isothiourea N-riboside, ensuing installment of a phosphomonoester at the 5′-hydroxyl of the ribosyl moiety followed by the conversion into the adenosine diphosphate. We use this method to obtain four regioisomers of ADP-ribosylated ubiquitin (Ub^ADPr^), each modified with an ADP-ribosyl residue on a different arginine position within the ubiquitin (Ub) protein (Arg42, Arg54, Arg72, and Arg74) as the first reported examples of fully synthetic arginine-linked ADPr-modified proteins. We show the chemically prepared Arg-linked Ub^ADPr^ to be accepted and processed by Legionella enzymes and compare the entire suite of four Arg-linked Ub^ADPr^ regioisomers in a variety of biochemical experiments, allowing us to profile the activity and selectivity of *Legionella pneumophila* ligase and hydrolase enzymes.

## Introduction

**Figure 1:**
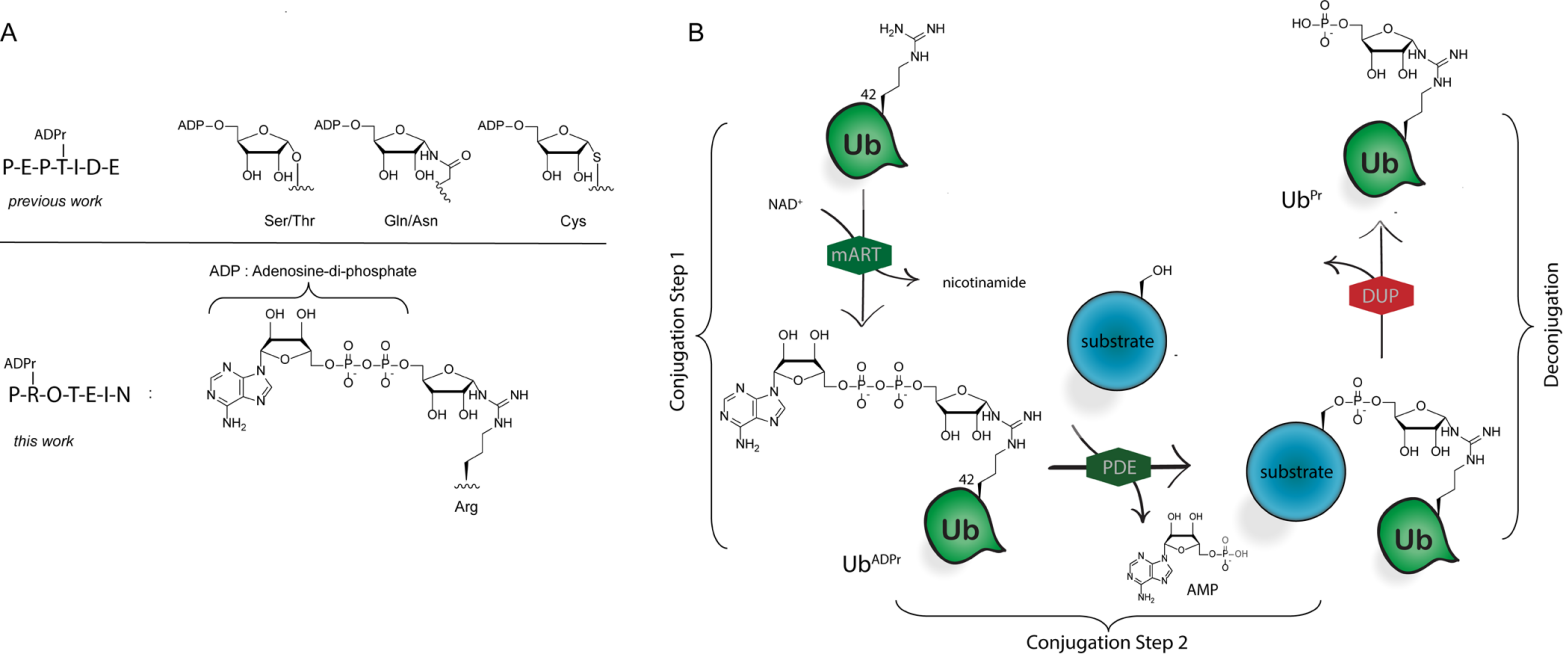
(A) Advances presented in this study and (B) schematic representation of the pathway *L. pneumophila* enzymes use to (de)ubiquitinate host cell substrate proteins.

Post-translational modification (PTM) of cellular proteins can affect their functioning and localization, influencing a wide range of cellular signaling processes. PTMs include relatively small groups such as a phosphate or methyl but can also involve more complex molecular entities such as (poly-)glycosides and ADP-ribose (ADPr)-moieties, or even entire proteins, such as ubiquitin (Ub). In the case of ADPr, mono-ADP-ribosyltransferases (mARTs) catalyze the displacement of nicotinamide from β-NAD^+^ by a nucleophilic amino acid side chain in the target protein, thereby effectively connecting ADP-ribose to the protein via an α-configured ribosyl linkage.^1,2^ As is the case for most PTMs, ADP-ribosylation is a highly dynamic process and specific writer (mART) and eraser (ADPr-hydrolase (ARH)) enzymes can act on specific proteins or amino acids.^3^ ARTs can be classified into two families, ART-C and ART-D, named after their first identification in cholera and diphtheria bacteria, respectively. ADP-ribosylation of the δ-guanidinium group of an arginine residue is typically catalyzed by the ART-C subfamily.^3−5^ The effector family of *Legionella pneumophila* SidE proteins (SdeA, SdeB, SdeC, and SidE) combines multiple enzymatic active domains in a single protein, including an ART-C-type domain and a phosphodiesterase (PDE) domain. Legionella uses these SidE proteins to hijack the eukaryotic host cell’s ubiquitin pathway and ubiquitinate host cell proteins in an unconventional manner. This multistep cascade starts with the Legionella SidE mART domain that catalyzes the attachment of ADPr on Arg42 of the host cell ubiquitin proteins. Subsequently, the phosphodiesterase (PDE) domain in SidE catalyzes the formation of a phosphodiester bond between the serine of host cell substrate protein and the arginine-linked Ub^ADPr^ while expelling adenosine monophosphate (Figure 1B).^6−9^

In this way, the bacterial effector enzyme effectively links host Ub to host substrate proteins via an arginine-phosphoribosyl linkage. It contrasts with the canonical ubiquitination process in which an isopeptide bond between the Ub C-terminal Gly76 carboxylic acid and ε-amine of a lysine residue in the substrate protein is formed by ubiquitin ligases. By using these SidE enzymes to achieve phosphoribosyl ubiquitination of host substrates and so-called deubiquitinases for phosphoribosyl ubiquitination (DUP) hydrolases to release the substrate protein in a deconjugation step, Legionella has dynamic control over part of the host cell’s ubiquitinome, predominantly ER- and Golgi-associated proteins, which allow the bacterium to create an environment in which it can effectively replicate.^10−12^ These SidE effectors are important for Legionella to proliferate in the host cell and effectively dodge the immune system, as bacterial replication is greatly reduced without these effectors.^13^ Synthetic ADP-ribosylated peptides and proteins and reagents based thereon are of great use in studying activities, preferences, and molecular mechanisms of (de)ADP-ribosylating enzymes. Chemical synthesis offers the possibility of preparing well-defined material on a scale that is useful for interrogating the complex biology associated with this PTM. We and others have previously reported on the synthesis of ADP-ribosylated peptides where ADP-ribose is attached to Ser, Thr or Cys,^14,15^ and Asn, Gln^16−18^ as well as to unnatural amino acids^19−21^ (Figure 1A). The closest reported Arg^ADPr^-mimicking isostere is Cit^ADPr^,^17^ resembling the natively linked Arg^ADPr^, but with the distinction that the guanidinium moiety of the arginine side chain is replaced by the urea side chain of citrulline. Besides the synthesis of mono-ADP-ribosylated peptides, solid support-based synthesis protocols for defined poly-ADPr chains have been developed.^22−25^ Recent advances in the chemical synthesis of stabilized ADPr-protein conjugates show that copper-catalyzed azide–alkyne cycloaddition (CuAAC)^26,27^ can be used to obtain functional mimics of ADP-ribosylated substrates. A semisynthetic approach based on a native chemical ligation/desulfurization methodology of a synthetic ADPr-peptide and a truncated expressed histone gave rise to ADP-ribosylated histones used to reveal the impact of serine ADP-ribosylation on the chromatin structure and function.^28^ Another powerful approach toward such modified histones is the use of chemoenzymatic methods to mono- or poly-ADP-ribosylate synthetic peptides on designated serine sites using PARP1 in isolation or in combination with HPF1 followed by native chemical ligation strategies to obtain modified histones.^29−31^ We here set out to develop a methodology that would be generally applicable in the synthesis of peptides ADP-ribosylated at arginine and expand this chemistry to the first entire chemical synthesis of a natively linked ADP-ribosylated protein, Ub^ADPr^. We validated the applicability of this approach by synthesizing Ub^ADPr^, with an ADPr residue on all four different Arg positions in Ub (Arg42, Arg54, Arg72, and Arg74).

## Results and Discussion

In our recent work, we use an orthogonally protected ribosylated amino acid in solid-phase peptide synthesis to yield a ribosylated peptide that can be turned into an ADPr-peptide by on-resin phosphitylation and subsequent pyrophosphate formation.^15^ Threonine-, serine-, and cysteine-linked ribosyl amino acids were thus prepared via the stereoselective glycosylation of a suitably protected amino acid acceptor with a ribosyl donor. However, such a direct glycosylation reaction is difficult to perform on the guanidinium group of arginine due to its high basicity. An alternative route toward glycosylated arginine building blocks uses a Lewis acid (silver-ion) promoted coupling of the less basic nucleophilic amine in the ornithine side chain to an α-oriented isothiourea glycoside,^32−35^ which proved to be useful for the solution-phase synthesis of glycosylarginine building blocks. This method is also suitable for Fmoc-based SPPS to synthesize arginine-linked glycopeptides^34,35^ and can even be adapted to perform glycosylations on a resin-bound peptide.^32,33^ We applied a similar strategy to couple an α-configured isothiourea riboside to the δ-amine of ornithine in resin-bound peptides. To the best of our knowledge, this is the first example showing such an isothiourea-based guanidinylation for furanoses.

### Synthesis of Isothiourea Riboside

**Scheme 1:**
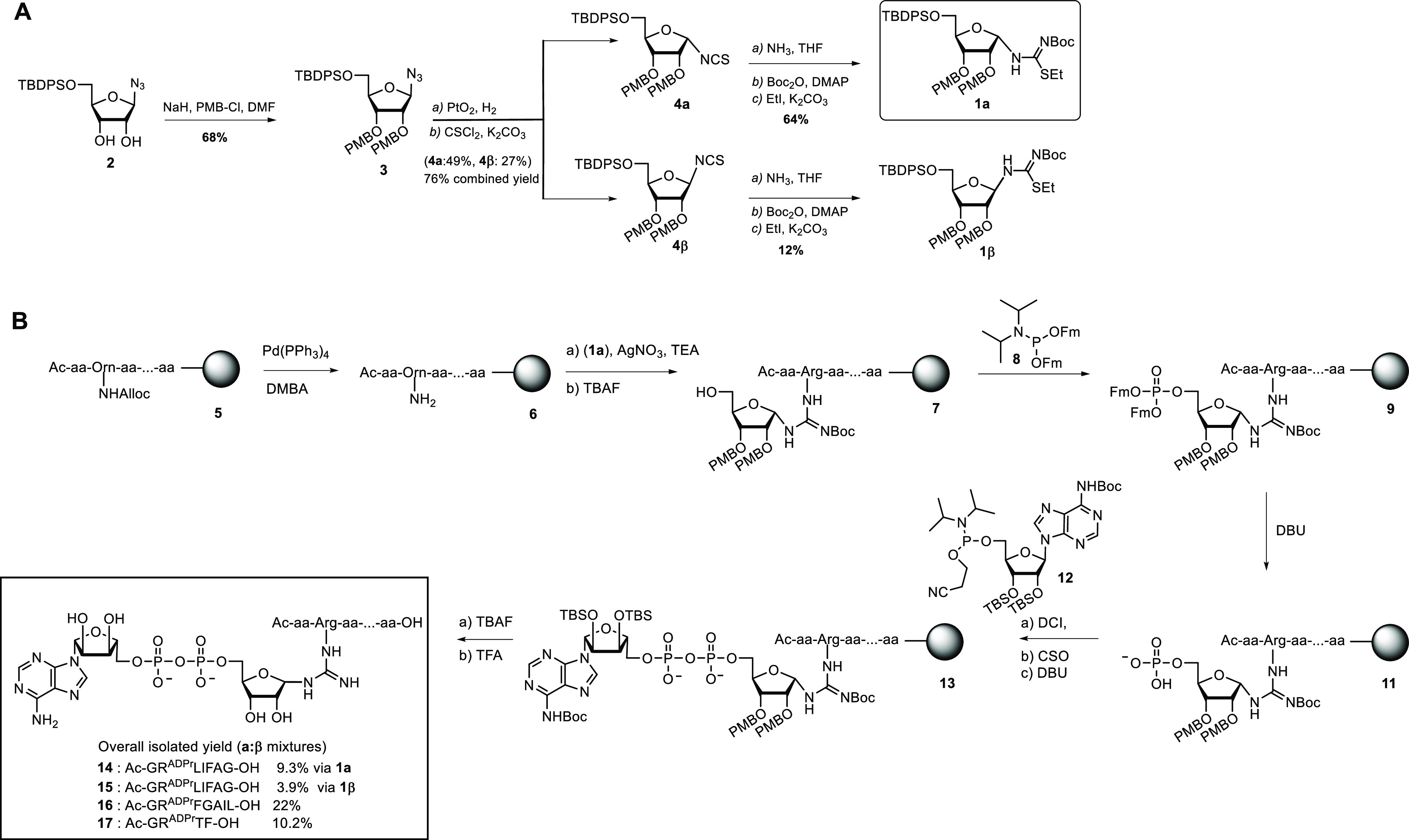
Synthetic Scheme toward Arginine-Linked ADPr-Peptides (A) Solution-phase chemistry toward building blocks **1α** and **1β** and (B) solid-phase chemistry toward ADPr-peptides **14**–**17**.

The synthesis of isothiourea ribosyl building block **1α** (Scheme 1A) started with the preparation of 5-*O*-((*tert*-butyl)-diphenylsilyl)-β-d-ribofuranosyl azide **2**, as described previously.^16,36^ PMB protection on the 2′- and 3′-hydroxyls in **2** yielded **3** in 68%. Next, the anomeric azide was reduced using Adam’s catalyst and H_2_. Attempts to work up the reaction proved unsuccessful as the resulting ribosylamine is highly labile and concentration *in vacuo* led to total degradation of the product. Therefore, after filtration over a pad of celite to remove the catalyst, the filtrate was directly used without further work-up or purification to install the isothiocyanate. The resulting anomeric mixture of isothiocyanates could easily be separated by column chromatography to obtain the α-anomer **4α** in a yield of 49% over the two steps. In addition, the β-anomer **4β** was obtained in a yield of 27% over the two steps. Next, α-anomer **4α** was subjected to ammonolysis using ammonia in THF to give the thiourea that was directly treated with Boc_2_O to protect the amine functionality, followed by treatment with iodoethane to furnish ribosyl isothiourea **1α** in a 64% yield. The same sequence of steps was performed to synthesize **1β** in a 12% yield.

### ADP-Ribosyl Peptide and Protein Synthesis

With ribosyl isothiourea **1α** in hand, the on-resin synthesis of model heptapeptide **14** (Ac-GR^ADPr^LIFAG-OH) was undertaken (Scheme 1B). Peptide **14** is derived from the human Ub protein and contains the amino acids 42–47 known to be ADP-ribosylated on the Arg42 residue by *L. pneumophila* effector enzymes. On the prospected ADP-ribosylation-site, N^δ^-Alloc-protected ornithine was incorporated into the peptide sequence. The Alloc protecting group allows for orthogonal on-resin deprotection with Pd(PPh_3_)_4_ to furnish the primary amine. When, after a test cleavage of an aliquot of resin, full removal of the Alloc-group was observed, peptide **6** was guanidinylated with building block **1α** using AgNO_3_ as a Lewis acid. After full deprotection and removal from the resin on a test sample, LC-MS analysis showed complete conversion with no notable side products detected. Next, on-resin desilylation of the 5′-OH on the ribosyl moiety was performed to yield resin **7** and the primary alcohol was subsequently phosphitylated using the appropriate Fm-protected phosphoramidite reagent **8**, followed by on-resin P^III^ to P^V^ oxidation. During this phosphorylation reaction, however, along with the desired product **9**, a side product **10** originating from the phosphitylation of the guanidine group was observed (see Table S1). We optimized this reaction and suppressed the formation of the side product by varying the activator (5-ethylthio-1H-tetrazole (ETT), tetrazole, or 4,5-dicyanoimidazole (DCI)) and equivalents of the respective phosphitylating reagent (2.5 and 5.0 equiv). Overphosphitylation could be largely suppressed when utilizing DCI as an activator with 2.5 equiv of the phosphitylating reagent. Subsequent 1,8-diazabicyclo[5.4.0]undec-7-ene (DBU)-mediated deprotection of the phosphotriester toward peptide **11** prepared the resin for P^V^ to P^III^ coupling with adenosine amidite **12** that bears TBS and Boc as protecting groups. Subsequent oxidation with (1S)-(+)-(10-camphorsulfonyl)oxaziridine (CSO) and removal of the cyanoethyl protective group on the pyrophosphate moiety with DBU led to the protected ADPr-peptide **13**. The silyl ethers on the adenosine moiety were removed by treatment of the resin with a 1 M TBAF solution. Finally, the peptide was cleaved from the resin using 10% trifluoroacetic acid (TFA) in DCM with concomitant loss of all remaining protecting groups (Boc and PMB). RP-HPLC purification of the crude mixture led to the isolation of **14** in a 9.3% overall yield (based on the initial loading of the resin), as the first example of a synthetic Arg-linked ADPr-peptide. While characterizing the Arg-ADPr-peptide **14** by ^1^H NMR, we observed a mixture of anomers in a ratio of 6:4 (α/β), although the isothiourea riboside used in the guanidinylation reaction was of the pure α-configuration. It has been reported by Oppenheimer et al. that Arg-ADPr is prone to spontaneous anomerization during purification under either buffered or acidic conditions leading to the 6:4 (α/β) ratio.^37−39^ In our methodology, we applied 10% TFA to release the Arg-ADPr-peptide conjugates from resin, which might thus potentially induce or even enhance anomerization. To examine this further, we coupled the pure β-configured isothiourea riboside **1β** to peptide **6** and conducted the full cycle to obtain ADPr-peptide **15**. Analysis of this ADPr-peptide revealed that a similar 6:4 ratio of anomers was formed, confirming that indeed during the liberation from the resin and deprotection of the peptides or its subsequent purification anomerization occurs toward the same anomeric equilibrium. We additionally also synthesized a randomized heptamer **16** and a shorter tetramer peptide **17** in 22 and 10.2% yields, respectively.

Our next aim was to extrapolate our synthetic methodology from peptides to proteins. Therefore, full-length ubiquitin in which Arg42 was replaced with N^δ^-Alloc-protected ornithine was prepared using SPPS. Chemical synthesis of ^Arg42^Ub^ADPr^ was performed using procedures similar to those used to obtain **14** (Scheme S1) and monitored via test cleavages on small resin samples. Alloc deprotection using Pd(0) chemistry exposed the amine of the ornithine moiety and on-resin guanidinylation with **1α** proceeded uneventfully (Figure S1). Subsequent phosphitylation and oxidation (Figure S2) and P^V^–P^III^ coupling (Figure S3) resulted in fully protected resin-bound ^Arg42^Ub^ADPr^. For the short peptide **14**, 10% TFA in DCM was sufficient to remove all protective groups, and under these conditions, the glycosidic bonds and the pyrophosphate moiety underwent minimal hydrolysis. For synthetic Ub^ADPr^, however, the Pbf protective groups on the three remaining arginine residues in Ub needed prolonged reaction times at higher TFA concentrations (routinely, 90.5% TFA is used for 2 h to deprotect synthetic Ub fully). Strikingly, considering the acid lability of glycosidic bonds and the intrinsic lability of the pyrophosphate bond, test cleavages in 90.5% TFA for 1.5 h on Ub^ADPr^ showed no notable traces of cleavage of these bonds and confirmed the formation of Ub^ADPr^. We confirmed this acid stability by the incubation of Ub^ADPr^ (and heptamer **14**) in TFA (90.5%) for 1.5 h (Figures S4 and S5). We observed the full-length protein to be more acid-stable than the heptamer peptide, observing neither glycosidic bond nor pyrophosphate bond cleavage, respectively. Using these conditions, full cleavage from the resin and global deprotection followed by HPLC purification yielded synthetic ^Arg42^Ub^ADPr^**18** in an overall yield of 1.8%. The introduction of the ADPr group on the other arginine residues in Ub can be achieved straightforwardly by incorporating the N^δ^-Alloc-protected ornithine in another position in the protein during SPPS. Hence, we successfully synthesized Ub^ADPr^ on Arg54, Arg72, or Arg74, obtaining conjugates **19**–**21** in 1.8, 1.2, and 1.7% overall isolated yields, respectively. All four Ub^ADPr^s were characterized by HRMS and SDS-PAGE (Figures S6–S10). We observed between 14 and 30% Ub^Pr^ in our samples and attribute this to inefficient pyrophosphate formation caused by incomplete coupling of the nucleoside phosphoramidite to ribose 5-phosphate on-resin, as we established ^Arg42^Ub^ADPr^ to be stable under acidic conditions (Figure S5).

### Validation on Legionella Enzymes

**Figure 2:**
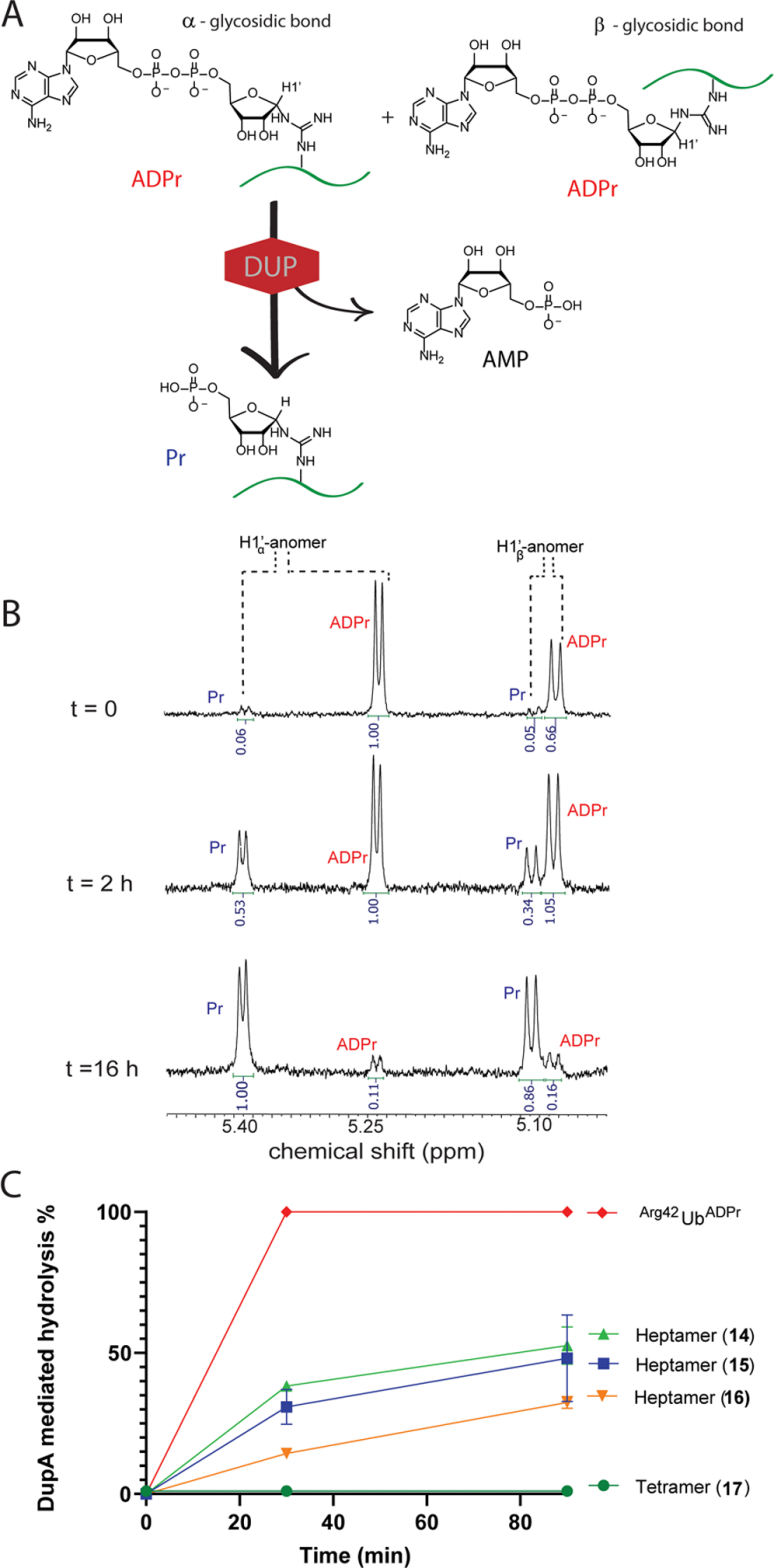
(A) Schematical representation of the experimental setup, where DupA cleaves the pyrophosphate linkage in α- or β-configured Arg-ADPr-peptides. (B) DupA-mediated hydrolysis of heptamer **14** followed over time using ^1^H NMR. The anomeric (α- or β-glycosidic-linked **14**) is hydrolyzed into the α- or β-linked phosphoribose variant providing different chemical shifts for each product. The associated protons are annotated and integrated. (C) DupA-mediated hydrolysis of **14**–**17** as compared to enzymatically produced ^Arg42^Ub^ADPr^. The conversion is measured over time and followed with HRMS. **14** is prepared using **1α**, and **15** is prepared using **1β**.

To investigate whether Legionella effector enzymes (DUPs) are able to hydrolyze the pyrophosphate in our synthetic ADPr-peptides or are affected by the anomeric configuration of the arginine-ribosyl linkage, we incubated Ub-derived Arg-ADPr heptapeptide **14** with DupA and followed the enzyme-mediated hydrolysis of the pyrophosphate bond over time using ^1^H NMR (Figure 2A). After 2 h, we observed hydrolysis of the ADPr-peptide (α-anomer, proton H1′: δ = 5.27 ppm, β-anomer, proton H1′: δ = 5.08 ppm) to the corresponding phosphoribosyl (Pr)-peptide (α-anomer, proton H1′: δ = 5.40 ppm, β-anomer, proton H1′: δ = 5.14 ppm) (Figure 2B) and a change in the initial 6:4 (α/β) ratio between the anomeric protons belonging to α- and β-anomers of the remaining ADPr-peptide. This verifies that our synthetic Arg-ADPr-peptide is being recognized and processed by the catalytic activity of the enzyme. Additionally, DupA seems to have a preference for α over β, hydrolyzing α-oriented Arg-ADPr-peptide **14** (roughly 1.5×) faster than its β-anomer. A similar observation has been reported previously for the recognition of Arg^ADPr^ by ARH1.^39^A measurement of the same sample after overnight incubation in the presence of DupA showed a near completion of the pyrophosphate hydrolysis reaction for both anomers and formation of both α- and β-phosphoribosyl peptide as major products. Although indeed both Ub^ADPr^ anomers appear to be processed by the enzyme over this extended time, we cannot conclude that DupA directly hydrolyzes the β-anomer (at a lower rate) or rather this hydrolysis is caused by spontaneous epimerization of the β-anomer to the α-anomer that gets processed by the enzyme. Likewise, the observed Ub^Pr^ β-anomer product of this enzymatic reaction might originate from anomerization of the α-anomer of Ub^Pr^ until the equilibrium ratio has been reached. Estimated *t*_1/2_ for anomerization of Arg^ADPr^ under physiological conditions is between 3 and 6 h, although no experimental determination has been conducted, and the rate of the related spontaneous anomerization of α-NADH to β-NADH was determined to be 3.1 × 10^–3^ min^–1^ (*t*_1/2_ = 4 h).^38^

**Figure 3:**
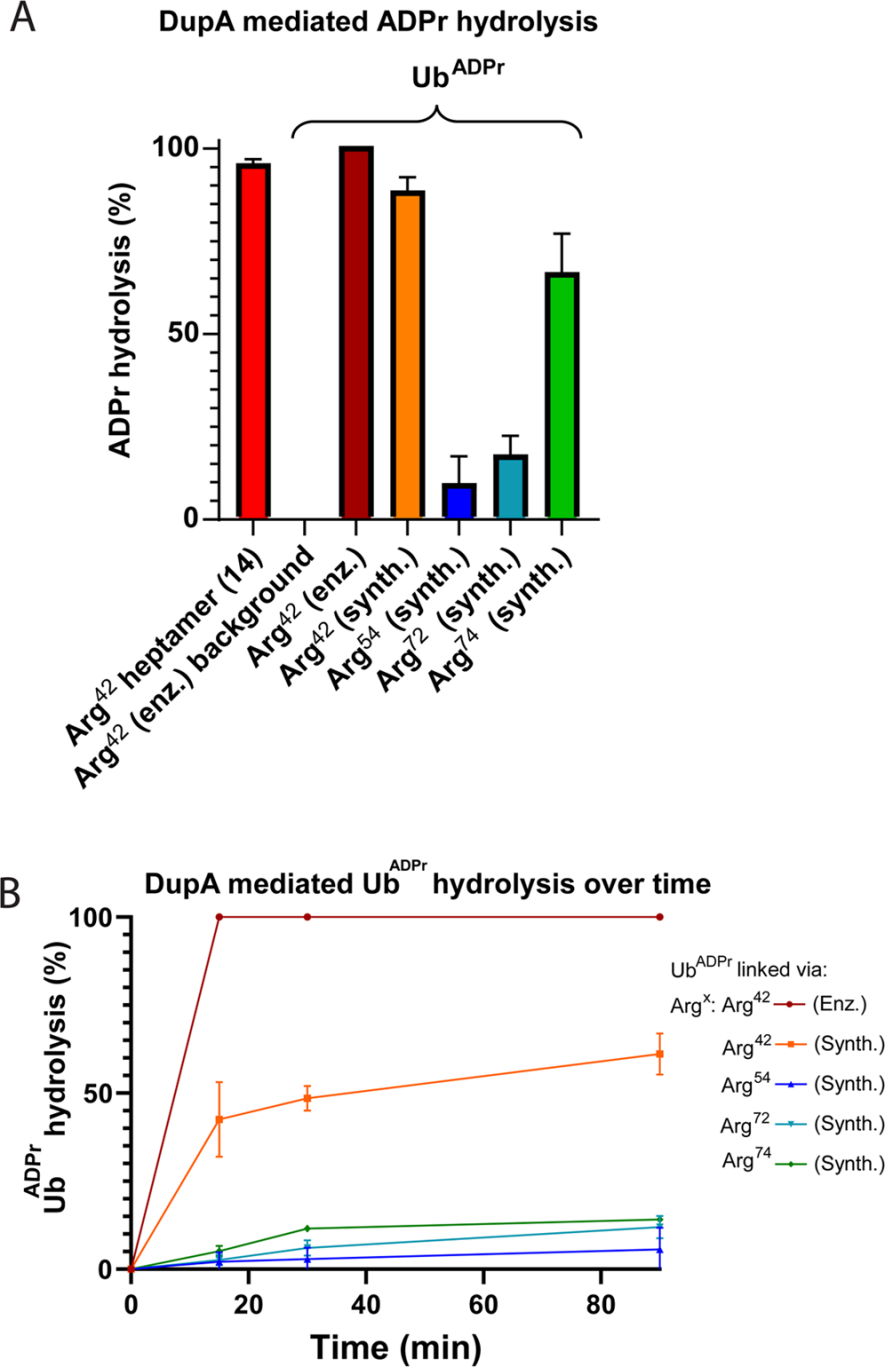
DupA-mediated hydrolysis of Ub^ADPr^ into Ub^Pr^. (A) DupA-mediated pyrophosphate bond cleavage in Ub^ADPr^ arginine variants after overnight incubation. (B) Hydrolysis of Ub^ADPr^ by DupA followed over a time course of 0–90 min. Both graphs are analyzed with HRMS. The measurements in both graphs are normalized for background Ub^Pr^ present as impurity associated with the synthesis.

Encouraged by the fact that DupA processes the synthetic ADPr-peptides, we next set out to compare the rate of hydrolysis with that of enzymatically produced ^Arg42^Ub^ADPr^ (Figures S11 and S12).^6^ We also included heptameric peptide **16**, randomized in the amino acid sequence surrounding the Arg42 Ub recognition site, and tetrapeptide **17** (Scheme 1), a sequence shorter in length and not derived from Ub. Enzymatic ^Arg42^Ub^ADPr^ was prepared by incubating ubiquitin with SdeA H277A mutant and NAD^+^ followed by purification using size-exclusion chromatography under buffered conditions at pH 7.5.^7^ The synthetic ADPr-peptides were incubated in the presence of DupA under buffered conditions and analyzed using high-resolution mass spectrometry at indicated times (Figure 2C). In this hydrolysis assay, the enzymatic ^Arg42^Ub^ADPr^ was completely hydrolyzed by DupA to ^Arg42^Ub^Pr^ within 30 min. Ubiquitin-derived heptamers **14** and **15** were processed at a rate lower than ^Arg42^Ub^ADPr^, showing 48 and 52% hydrolysis after 90 min, respectively. The sequence surrounding Arg42 of Ub seems to affect recognition by DupA as scrambled heptamer **16** was processed significantly slower (32% after 90 min) and tetramer **17** was not hydrolyzed by DupA at all. It hence seems that DupA can recognize the specific peptide context and/or peptide length of the Ub surrounding position 42. Not surprisingly, the full-length ^Arg42^Ub^ADPr^ protein, being the native substrate for Legionella effector proteins, provides more sequence context and structure and is hydrolyzed more efficiently than **14**–**15**, although we cannot exclude that anomerization of **14**–**15** might also contribute to the observed reduced rate of hydrolysis. We next examined the recognition and hydrolysis of our four synthetic Ub^ADPr^ proteins **18**–**21**, further annotated as (synth.), in comparison to enzymatically prepared ^Arg42^Ub^ADPr^, further annotated as (enz.), by incubating the respective Ub^ADPr^-analogues with DupA. We first analyzed if the synthetic conjugates were processed at all during overnight incubation with DupA and observed hydrolysis of all four synthetic Ub^ADPr^’s, albeit in different amounts (see Figure 3A). This hydrolysis is DupA-mediated as the incubation of enz. ^Arg42^Ub^ADPr^ in buffer without DupA does not lead to hydrolysis at these prolonged times. Synthetic ^Arg42^Ub^ADPr^**18** and Arg42-derived Ub^ADPr^ heptamer **14** were almost completely processed, as is enz. ^Arg42^Ub^ADPr^. Although less than ^Arg42^Ub^ADPr^, ^Arg74^Ub^ADPr^ is hydrolyzed significantly in contrast to ^Arg54^Ub^ADPr^ and^Arg72^Ub^ADPr^. Performing a similar assay and analyzing the conversion at earlier time points (15–90 min) showed enz. ^Arg42^Ub^ADPr^ to be completely hydrolyzed after 15 min. The processing of synth. ^Arg42^Ub^ADPr^ was less pronounced in this time frame (52% after 30 min) and (65% after 90 min) (Figure 3B), whereas the other three Ub^ADPr^’s linked via Arg72, Arg74, and Arg54 showed significantly less hydrolysis by DupA, complementing the demonstrated preference of DupA for Arg42. The initial swift turnover of roughly half the synth. ^Arg42^Ub^ADPr^ could be the processing of the α-anomer at a rate comparable to enz. ^Arg42^Ub^ADPr^.

The subsequent slower hydrolysis after this 50% mark might be indicating that either the β-anomer is processed by the enzyme at a reduced rate or that the β-anomer spontaneously anomerizes over time to give the α-anomer that, in turn, is processed by the enzyme. We set out to examine whether the use of β-thioisourea ribose **1β** instead of **1α** in the synthesis of ^Arg42^Ub^ADPr^ would lead to an ADPr-protein that is processed differently by the DupA enzyme and synthesized ^Arg42^Ub^ADPr^ (**22**) via the coupling of β-isothiourea **1β** to the ornithine side chain, followed by the installation of the ADP-ribosyl group at Arg42. Interestingly, **22** is processed to the same extent as ^Arg42^Ub^ADPr^ synthesized using α-riboside **1α**, indicating a comparable anomeric ratio after synthesis/isolation, as observed for peptides **14** and **15** (Figure S13). The observed difference between enz. ^Arg42^Ub^ADPr^ and synth. ^Arg42^Ub^ADPr^ is striking, and we speculate this reduced processing rate to be caused by anomerization during synthesis of the ADPr-protein, as shown using ^1^H NMR for synthetically prepared heptapeptide **14** (Figure 2C). We wondered whether enzymatically prepared Ub^ADPr^ also anomerizes spontaneously under physiological conditions. It is speculated in the literature that such a spontaneous anomerization of ADP-ribosylated proteins *in vivo* might not occur due to the physical stabilization of the ADPr group by the protein context, in contrast to the ADP-ribosylated-arginine in *in vitro* settings.^39^ If indeed the formed α-configurated Ub^ADPr^ is stabilized by Ub’s C-terminal tail, this might explain that enzymatically produced ^Arg42^Ub^ADPr^ retains the α-configuration, while the synthetic ^Arg42^Ub^ADPr^ anomerizes completely during the unfolded state in the SPPS protocol.

**Figure 4:**
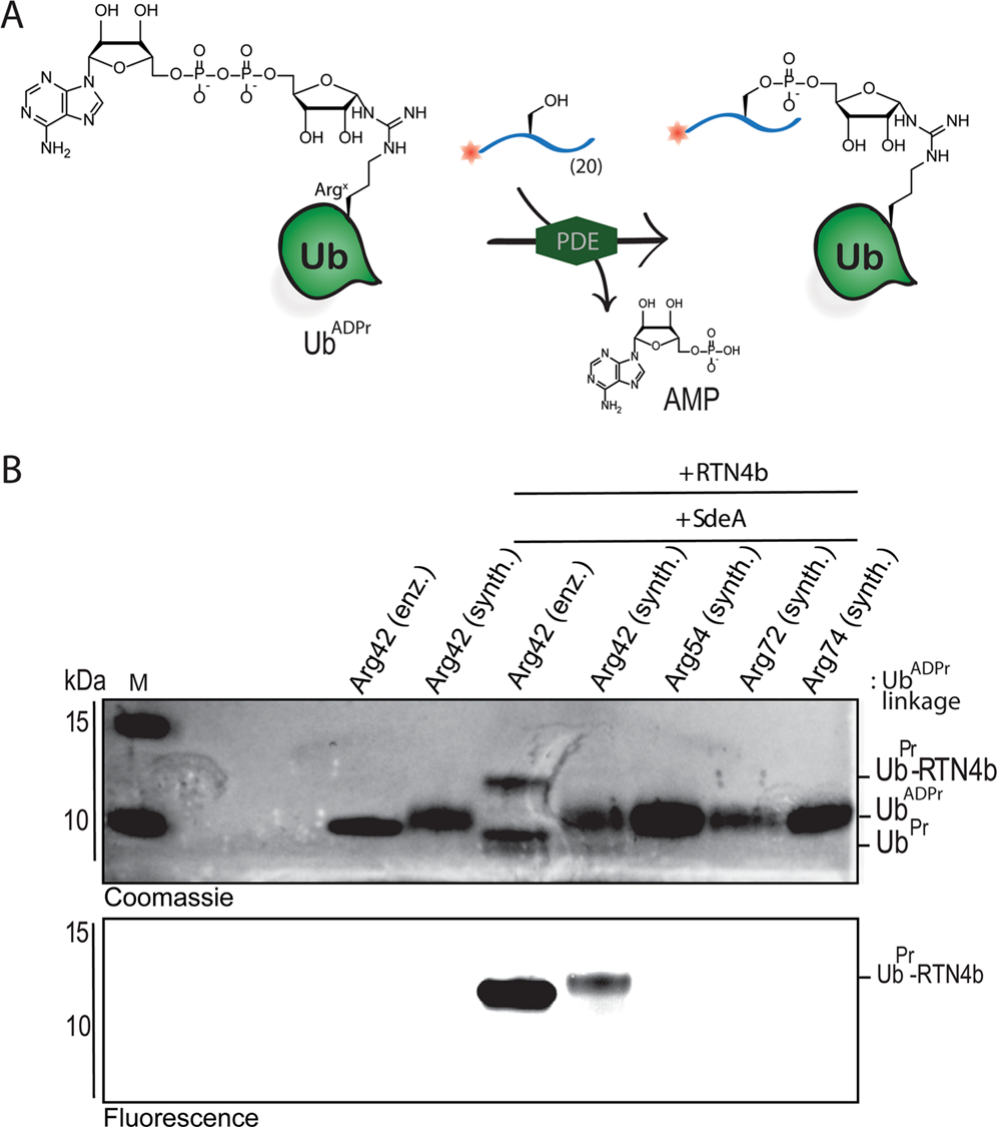
SdeA-mediated ligation of Ub^ADPr^ and fluorescent RTN4b 20-mer fragment **23**. (A) Schematic representation of the conducted assay showing SdeA-mediated conjugation of Ub^ADPr^ and peptide **23** to form a fluorescent product. (B) ^Arg42^Ub^ADPr^ is recognized and processed by SdeA. SdeA-mediated ligation assay performed for all (synthetic) Ub^ADPr′^s and analyzed by SDS-PAGE; top panel: gel stained with Coomassie blue protein stain, bottom panel; fluorescence scan. M: molecular weight marker.

Our next aim was to investigate the SdeA-mediated ligation of substrate ER-proteins to Ub^ADPr^, the critical biological process in the onset of Legionnaires’ disease.^40^ We synthesized a 20-mer peptide (sequence on page S29) derived from the ER remodeling RTN4b protein (**23**) known to be a substrate of SidE effectors,^8^ equipped with a rhodamine fluorophore on the N-terminus. We tested whether SdeA, using its PDE domain, would ligate Ub^ADPr^ to this RTN4b peptide to form a fluorescent peptide-Pr-Ub conjugate (Figure 4A). The full RTN4b 20-mer peptide **23** contains six serine residues as potential conjugation sites.^10^ Enzymatically produced ^Arg42^Ub^ADPr^ was incubated with SdeA and **23** as control and analyzed by mass spectrometry (Figure S14). Under the used conditions, SdeA couples ^Arg42^Ub^ADPr^ to peptide **23** to form the phosphoribosyl-linked ^Arg42^Ub-RTN4b product (Figure 4A) and shows partial hydrolysis of the pyrophosphate bond to ^Arg42^Ub^Pr^, as has been reported.^6,10^ This confirms that peptide **23** is a suitable substrate for inducing the PDE-mediated ligation of ^Arg42^Ub^ADPr^. We next examined if our four synthetic ubiquitins **18–21** could also participate in this process. LC-MS analysis confirmed the formation of the product for (synth.) ^Arg42^Ub^ADPr^**18** although the conversion was lesser compared to the enzymatic material (Figure S15). We then used SDS-PAGE analysis to compare the ligation of **23** to the enzymatic and synthetic Ub^ADPr′^s. Indeed, fluorescent product formation could clearly be visualized by in-gel fluorescence for the enzymatic ^Arg42^Ub^ADPr^ and synthetic ^Arg42^Ub^ADPr^ (Figure 4B). Synth. Ub^ADPr^ modified at Arg54, Arg72, and Arg74 (**19**–**21**) were neither coupled to RTN4b peptide **23** nor hydrolyzed by SdeA, also showing the preference of the SdeA ligase activity for the Arg42 position. Synthetic ^Arg42^Ub^ADPr^ is coupled to **23** by SdeA significantly less than enzymatically produced ^Arg42^Ub^ADPr^, which might be caused by the degree of anomerization in the former. Since β-NAD^+^ is coupled to Ub by the mART domain of SdeA, the expected product (Ub^ADPr^) carries the α-orientation and hence the PDE domain of SdeA would facilitate the coupling of the RTN4b-derived peptide to only the α-Ub^ADPr^. The presence of the β-Ub^ADPr^ might hinder efficient coupling to peptide **23** by competing for entry toward the active site of the SdeA PDE domain.

## Conclusions

We developed a methodology to synthesize arginine-linked ADPr-peptides and Ub^ADPr^ proteins, showcasing the first total chemical synthesis of an ADP-ribosylated protein carrying a native arginine linkage. Our synthetic strategy features a Lewis acid-mediated on-resin guanidinylation of the primary amine in the ornithine side chain of the protein with a thioisourea riboside to furnish the native Arg-ribosyl residue. Subsequent phosphorylation and formation of the adenosine-di-phosphate were also conducted on resin. After global deprotection and resin release using acidic conditions, the ADP-ribosylated proteins were purified using RP-HPLC. This methodology to install the N-glycosidic linkage and sequentially build up the ADP-moiety was effective, and the product proved resistant to a high concentration of TFA during deprotection. Of note, the synthetic Ub^ADPr^’s contain varying amounts of phosphoribosylated protein, indicating that the adenosine-di-phosphate formation reaction was not quantitative. The ADPr-peptides and ADPr-ubiquitin regioisomers were recognized by Legionella effectors (DupA and SdeA) in hydrolysis and ligation assays, albeit at a lower rate than the enzymatically produced Ub^ADPr^. We speculate this reduced processing to be caused by the anomerization of the N-glycoside linkage in Arg-ADPr that connects ribose to the side chain of arginine. Although anomerization is known to occur under physiological conditions, the conditions used to prepare synthetic Ub^ADPr^ might contribute to an increased degree of anomerization, leading to a slower processing by the Legionella hydrolase. The ability to site-specifically introduce the ADPr moiety allowed us to synthesize Ub^ADPr^ on every arginine residue of ubiquitin (Arg42, Arg54, Arg72, Arg74), giving access to well-defined material currently not attainable using biochemical methods. In hydrolysis and ligation assays, we demonstrate that Legionella effectors DupA and SdeA favor the ^Arg42^Ub^ADPr^ linkage. We hence developed a synthetic approach that provides peptides and proteins with the native ADPr-amino acid junction that were used to profile the site-specificity of enzymes involved in installing and removing ADPr-modifications.

## Supporting Information Available

Synthetic procedures and analysis data of key compounds and products, including NMR and HRMS analyses (PDF)

## Abbreviations

Ub: ubiquitin
ADPr: adenosine-di-phosphate-ribose

## References

[ref1] Ueda K.; Hayaishi O.; Oka J.; Komura H.; Nakanishi K. ADP-Ribosylation of Proteins; Althaus F. R.; Hilz H.; Shall S., Eds.; Springer: Berlin, 1985; pp 159–166.

[ref2] Zhou G. C.; Parikh S. L.; Tyler P. C.; Evans G. B.; Furneaux R. H.; Zubkova O. V.; et al. Inhibitors of ADP-ribosylating bacterial toxins based on oxacarbenium ion character at their transition states. J. Am. Chem. Soc. 2004, 126, 5690–5698. 10.1021/ja038159+. 15125661

[ref3] Cohen M. S.; Chang P. Insights into the biogenesis, function, and regulation of ADP-ribosylation. Nat. Chem. Biol. 2018, 14, 236–243. 10.1038/nchembio.2568. 29443986 PMC5922452

[ref4] Palazzo L.; Mikoč A.; Ahel I. ADP-ribosylation: new facets of an ancient modification. FEBS J. 2017, 284, 2932–2946. 10.1111/febs.14078. 28383827 PMC7163968

[ref5] Hottiger M. O.; Hassa P. O.; Lüscher B.; Schüler H.; Koch-Nolte F. Toward a unified nomenclature for mammalian ADP-ribosyltransferases. Trends Biochem. Sci. 2010, 35, 208–219. 10.1016/j.tibs.2009.12.003. 20106667

[ref6] Bhogaraju S.; Kalayil S.; Liu Y.; Bonn F.; Colby T.; Matic I.; Dikic I. Phosphoribosylation of Ubiquitin Promotes Serine Ubiquitination and Impairs Conventional Ubiquitination. Cell 2016, 167, 1636–1649. 10.1016/j.cell.2016.11.019. 27912065

[ref7] Kalayil S.; Bhogaraju S.; Bonn F.; Shin D.; Liu Y.; Gan N.; Basquin J.; Grumati P.; Luo Z.-Q.; Dikic I. Insights into catalysis and function of phosphoribosyl-linked serine ubiquitination. Nature 2018, 557, 734–738. 10.1038/s41586-018-0145-8. 29795347 PMC5980784

[ref8] Akturk A.; Wasilko D. J.; Wu X.; Liu Y.; Zhang Y.; Qiu J.; Luo Z. Q.; Reiter K. H.; Brzovic P. S.; Klevit R. E.; Mao Y. Mechanism of phosphoribosyl-ubiquitination mediated by a single legionella effector. Nature 2018, 557, 729–733. 10.1038/s41586-018-0147-6. 29795346 PMC5980775

[ref9] Dong Y.; Mu Y.; Xie Y.; Zhang Y.; Han Y.; Zhou Y.; Wang W.; Liu Z.; Wu M.; Wang H.; Pan M.; Xu N.; Xu C.-Q.; Yang M.; Fan S.; Deng H.; Tan T.; Liu X.; Liu L.; Li J.; Wang J.; Fang X.; Feng Y. Structural basis of ubiquitin modification by the Legionella effector SdeA. Nature 2018, 557, 674–678. 10.1038/s41586-018-0146-7. 29795342

[ref10] Shin D.; Mukherjee R.; Liu Y.; Gonzalez A.; Bonn F.; Liu Y.; Rogov V. V.; Heinz M.; Stolz A.; Hummer G.; Dötsch V.; Luo Z.-Q.; Bhogaraju S.; Dikic I. Regulation of Phosphoribosyl-Linked Serine Ubiquitination by Deubiquitinases DupA and DupB. Mol. Cell 2019, 77, 164–179. 10.1016/j.molcel.2019.10.019. 31732457 PMC6941232

[ref11] Wan M.; Sulpizio A. G.; Akturk A.; Beck W. H. J.; Lanz M.; Faca V. M.; Smolka M. B.; Vogel J. P.; Mao Y. Deubiquitination of phosphoribosyl-ubiquitin conjugates by phosphodiesterase-domain–containing Legionella effectors. Proc. Natl. Acad. Sci. U.S.A. 2019, 116, 23518–23526. 10.1073/pnas.1916287116. 31690664 PMC6876201

[ref12] Liu Y.; Mukherjee R.; Bonn F.; Colby T.; Matic I.; Glogger M.; Heilemann M.; Dikic I. Serine-ubiquitination regulates Golgi morphology and the secretory pathway upon Legionella infection. Cell Death Differ. 2021, 28, 2957–2969. 10.1038/s41418-021-00830-y. 34285384 PMC8481228

[ref13] Bardill J. P.; Miller J. L.; Vogel J. P. IcmS-dependent translocation of SdeA into macrophages by the *Legionella pneumophila* type IV secretion system. Mol. Microbiol. 2005, 56, 90–103. 10.1111/j.1365-2958.2005.04539.x. 15773981

[ref14] Voorneveld J.; Rack J. G. M.; Ahel I.; Overkleeft H. S.; Van Der Marel G. A.; Filippov D. V. Synthetic α- And β-Ser-ADP-ribosylated Peptides Reveal α-Ser-ADPr as the Native Epimer. Org. Lett. 2018, 20, 4140–4143. 10.1021/acs.orglett.8b01742. 29947522 PMC6038095

[ref15] Voorneveld J.; Rack J. G. M.; Gijlswijk L.; Meeuwenoordm N. J.; Liu Q.; Overkleeft H. S.; Van Der Marel G. A.; Ahe I.; Filippov D. V. Molecular Tools for the Study of ADP-Ribosylation: A Unified and Versatile Method to Synthesise Native Mono-ADP-Ribosylated Peptides. Chem. – Eur. J. 2021, 27, 10621–10627. 10.1002/chem.202100337. 33769608 PMC8360141

[ref16] van der Heden van Noort G. J.; van der Horst M. G.; Overkleeft H. S.; van der Marel G. A.; Filippov D. V. Synthesis of mono-ADP-ribosylated oligopeptides using ribosylated amino acid building blocks. J. Am. Chem. Soc. 2010, 132, 5236–5240. 10.1021/ja910940q. 20232863

[ref17] Kistemaker H. A. V.; Nardozza A. P.; Overkleeft H. S.; van der Marel G. A.; Ladurner A. G.; Filippov D. V. Synthesis and Macrodomain Binding of Mono-ADP-Ribosylated Peptides. Angew. Chem., Int. Ed. 2016, 55, 10634–10638. 10.1002/anie.201604058. 27464500

[ref18] Speciale G.; Bernardi A.; Nisic F. A facile Synthesis of α-N-Ribosyl-Asparagine and α-N-Ribosyl-GLutamine Building Building Block. Molecules 2013, 18, 8779–8785. 10.3390/molecules18088779. 23887719 PMC6270248

[ref19] Moyle P. M.; Muir T. W. Method for the synthesis of Mono-ADP-ribose Conjugated Peptides. J. Am. Chem. Soc. 2010, 132, 15878–15880. 10.1021/ja1064312. 20968292 PMC3010531

[ref20] Li L.; Li Q.; Ding S.; Xin P.; Zhang Y.; Huang S.; Zhang G. ADP-ribosyl-N_3_: A versatile precursor for divergent syntheses of ADP-ribosylated compounds. Molecules 2017, 22, 1346 10.3390/molecules22081346. PMC6152188 28805740

[ref21] Zhu A.; Li X.; Bai L.; Zhu G.; Guo Y.; Lin J.; Cui Y.; Tian G.; Zhang L.; Wang J.; Li X. D.; Li L. Biomimetic α-selective ribosylation enables two-step modular synthesis of biologically important ADP-ribosylated peptides. Nat. Commun. 2020, 11, 5600 10.1038/s41467-020-19409-1. 33154359 PMC7645758

[ref22] van der Heden van Noort G. J.; Overkleeft H. S.; van der Marel G. A.; Filippov D. V. Ribosylation of Adenosine: An Orthogonally Protected Building Block for the Synthesis of ADP-Ribosyl Oligomers. Org. Lett. 2011, 13, 2920–2923. 10.1021/ol200971z. 21561136

[ref23] Kistemaker H. A. V.; Overkleeft H. S.; van der Marel G. A.; Filippov D. V. Branching of poly(ADP-ribose): Synthesis of the Core Motif. Org. Lett. 2015, 17, 4328–4331. 10.1021/acs.orglett.5b02143. 26307949

[ref24] Liu Q.; Kistemaker H. A. V.; Overkleeft H. S.; van der Marel G. A.; Filippov D. V. Synthesis of ribosyl-ribosyl-adenosine-5′,5″,5‴(triphosphate)—the naturally occurring branched fragment of poly(ADP ribose). Chem. Commun. 2017, 53, 10255–10258. 10.1039/C7CC05755E. 28868552

[ref25] Kistemaker H. A. V.; Lameijer L. N.; Meeuwenoord N. J.; Overkleeft H. S.; van der Marel G. A.; Filippov D. V. Synthesis of Well-Defined Adenosine Diphosphate Ribose Oligomers. Angew. Chem., Int. Ed. 2015, 54, 4915–4918. 10.1002/anie.201412283. 25704172

[ref26] Liu Q.; Kistemaker H. A. V.; Bhogaraju S.; Dikic I.; Overkleeft H. S.; van der Marel G. A.; Ovaa H.; van der Heden van Noort G. J.; Filippov D. V. A General Approach Towards Triazole-Linked Adenosine Diphosphate Ribosylated Peptides and Proteins. Angew. Chem., Int. Ed. 2018, 57, 1659–1662. 10.1002/anie.201710527. 29215186

[ref27] Kim R. Q.; Misra M.; Alexis G.; Thomaškoviç I.; Shin D.; Schinderlin H.; FIlippov D. V.; Ovaa H.; Dikic I.; van der Heden van Noort G. J. Development of ADPribosyl ubiquitin analogs to study enzymes involved in Legionella infection. Chem. – Eur. J. 2021, 27, 2506–2512. 10.1002/chem.202004590. 33075184 PMC7898697

[ref28] Hananya N.; Daley S. K.; Bagert J. D.; Muir T. W. Synthesis of ADP-Ribosylated Histones Reveals Site-Specific Impacts on Chromatin Structure and Function. J. Am. Chem. Soc. 2021, 143, 10847–10852. 10.1021/jacs.1c05429. 34264659

[ref29] Mohapatra J.; Tashiro K.; Beckner R. L.; Sierra J.; Kilgore J. A.; Williams N. S.; Liszczak G. Serine ADP-ribosylation marks nucleosomes for ALC1-dependent chromatin remodeling. eLife 2021, 10, e71502 10.7554/eLife.71502. 34874266 PMC8683085

[ref30] Bonfiglio J. J.; Leidecker O.; Dauben H.; Longarini E. L.; Colby T.; Segundo-Acosta P. S.; Perez K. A.; Matic I. An HPF1/PARP1-Based Chemical Biology Strategy for Exploring ADP-Ribosylation. Cell 2020, 183, 1086–1102. 10.1016/j.cell.2020.09.055. 33186521

[ref31] Tashiro K.; Mohapatra J.; Brautigam C. A.; Liszczak G. A Protein Semisynthesis-Based Strategy to Investigate the Functional Impact of Linker Histone Serine ADP-Ribosylation. ACS Chem. Biol. 2022, 17, 810–815. 10.1021/acschembio.2c00091. 35312285 PMC10202128

[ref32] Pan M.; Li S.; Li X.; Shao F.; Liu L.; Hu H. G. Synthesis of and specific antibody generation for glycopeptides with arginine N-GlcNAcylation. Angew. Chem., Int. Ed. 2014, 53, 14517–14521. 10.1002/anie.201407824. 25353391

[ref33] Li X.; Krafczyk R.; Macošek J.; Li L.; Zou Y.; Simon B.; Pan X.; Wu Q. Y.; Yan F.; Li S.; Hennig J.; Jung K.; Lassak J.; Hu H. G. Resolving the α-glycosidic linkage of arginine-rhamnosylated translation elongation factor P triggers generation of the first ArgRha specific antibody. Chem. Sci. 2016, 7, 6995–7001. 10.1039/C6SC02889F. 28451135 PMC5363779

[ref34] Wang S.; Corcilius L.; Sharp P. P.; Rajkovic A.; Ibba M.; Parker B. L.; Payne R. J. Synthesis of rhamnosylated arginine glycopeptides and determination of the glycosidic linkage in bacterial elongation factor P. Chem. Sci. 2017, 8, 2296–2302. 10.1039/C6SC03847F. 28451332 PMC5363394

[ref35] Wang S.; Corcilius L.; Sharp P. P.; Payne R. J. Synthesis of a GlcNAcylated arginine building block for the solid phase synthesis of death domain glycopeptide fragments. Bioorg. Med. Chem. 2017, 25, 2895–2900. 10.1016/j.bmc.2017.03.012. 28320614

[ref36] Štimac A.; Kobe J. An improved preparation of 2,3,5-tri-O-acyl-β-d-ribofuranosyl azides by the Lewis acid-catalysed reaction of β-d-ribofuranosyl acetates and trimethylsilyl azide: an example of concomitant formation of the α anomer by trimethylsilyl triflate catalysis. Carbohydr. Res. 1992, 232, 359–365. 10.1016/0008-6215(92)80068-C.

[ref37] Oppenheimer N. J. Structural determination and stereospecificity of the choleragen-catalyzed reaction of NAD+ with guanidines. J. Biol. Chem. 1978, 253, 4907–4910. 10.1016/S0021-9258(17)34632-X. 209022

[ref38] Oppenheimer N. J. ADP-Ribosylarginine. Methods Enzymol. 1984, 106, 399–403. 10.1016/0076-6879(84)06042-0. 6436643

[ref39] Moss J.; Oppenheimer N. J.; West R. E.; Stanley S. J. Amino acid specific ADP-ribosylation: substrate specificity of an ADP-ribosylarginine hydrolase from turkey erythrocytes. Biochemistry 1986, 25, 5408–5414. 10.1021/bi00367a010. 3778868

[ref40] Puvar K.; Saleh A. M.; Curtis R. W.; Zhou Y.; Nvalapatla P. R.; Fu J.; Rovira A. R.; Tor Y.; Luo Z. Q.; Ghosh A. K.; Wirth M. J.; Chmielewski J.; Kinzer-Ursem T. L.; Das C. Fluorescent Probes for Monitoring Serine Ubiquitination. Biochemistry 2020, 59, 1309–1313. 10.1021/acs.biochem.0c00067. 32207972 PMC7294441

