# Supplementary material for: Arginine ADP-Ribosylation: Chemical Synthesis of Post-Translationally Modified Ubiquitin Proteins
Source: J Am Chem Soc. 2022 Nov 1;144(45):20582–9. doi: 10.1021/jacs.2c06249 (PMC9673145; doi:10.1021/jacs.2c06249)
Supplement: Supplementary file 1 — ja2c06249_si_001.pdf [file ja2c06249_si_001.pdf]

Supporting information

Arginine ADP-ribosylation: Chemical Synthesis of Post-Translationally Modified Ubiquitin Proteins

Jim Voorneveld[a,1], Max S. Kloet[b,1], Sven Wijngaarden[a] Robbert Q. Kim[b], Angeliki Moutsiopoulou[b], Marnix Verdegaal[b], Mohit Misra[c], Ivan Đikić[c], Gijsbert A. van der Marel, Herman S. Overkleeft, Dmitri V. Filippov[a] and Gerbrand J. van der Heden van Noort[b*]*

Page

Table S1. Screening of the reaction conditions of the phosphorylation reaction from peptide 7 to 9……………………………………………………………S2

Scheme S1. Synthetic scheme towards arginine linked Ub^{ADPr}'s 18-21 ……………………S3

Figure S1-3. HRMS spectra of intermediates in the synthesis of $^{R42}Ub^{ADPr}$ (18) ……………S4-S6

Figure S4. LC-MS of heptamer (14) treated with TFA …………………………………… S7

Figure S5. Acid stability of Arg-ADPr compounds……………………………………… S7

Figure S6-S9 HRMS spectra of purified $^{Rx}Ub^{ADPr}$(18-21)……………………………S8-S11

Figure S10. SDS-PAGE analysis of $^{Rx}Ub^{ADPr}$ (18-21)……………………………………S12

Figure S11. HRMS spectra enzymatically prepared $^{R42}Ub^{ADPr}$……………………………S13

Figure S12. HRMS spectra of the DupA mediated hydrolysis of enzym. $^{R42}Ub^{ADPr}$……………S14

Figure S13. Hydrolysis of $^{R42}Ub^{ADPr}$'s synthesized via 1α (18) or 1β (22) by DupA…………S15

Figure S14. HRMS of the SdeA-mediated ligation of enzym. $^{R42}Ub^{ADPr}$ and RTN4B (23)…………S16

Figure S15.HRMS of the SdeA-mediated ligation of synth. $^{R42}Ub^{ADPr}$ (16) and RTN4b (23)…………S17

Figure S16. SDS-PAGE analysis SdeA-mediated ligation of enzym. $^{R42}Ub^{ADPr}$ or synth. $^{Rx}Ub^{ADPr}$ (18-21) and RTN4B peptide fragment (23)……………………………………… S12

General synthetic procedures……………………………………………………S19-S23

Synthesis of peptides (14-17)……………………………………………………S23-26

Synthesis of full-length $^{Rx}Ub^{ADPr}$'s (18-22)……………………………………S26-29

Synthesis RTN4B fragment (23) ……………………………………………………… S29

Procedures DupA-mediated hydrolysis assays and SdeA-mediated ligation…………………S30-31

References………………………………………………………………………………S31

^{1}H-, ^{13}C-, COSY-, HSQC- ^{31}P-NMR spectra of riboses (1-4) and NMR and HRMS spectra of peptides (14-17)……………………………………………………………………S32-S54

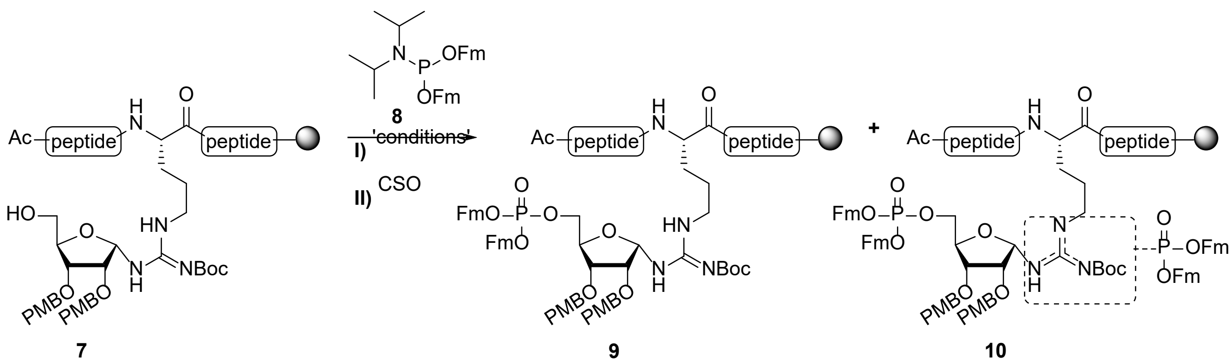

entry	activator	Eq. of 8	Conc. (mM)	Product 9 (%)	side-product 10 (%)
**1**	ETT	5	88	36	64
**2**	ETT	2.5	44	60	40
**3**	Tetrazole	5	88	35	65
**4**	Tetrazole	2.5	44	60	40
**5**	DCI	5	88	44	56
**6**	DCI	2.5	44	84	16

Table S1. Screening of the reaction conditions of the phosphorylation reaction from peptide 7 to 9. Product ratios were calculated by integration of the peak area of UV absorptions in their LC-MS trace. Every reaction was carried out on a 10 µmol scale with peptide **7**. The equivalents listed in the table are the equivalents of Fm-protected amidite **8** relative to **7**. The concentration is calculated relative to the phosphorylating reagent, 2.0 equivalents of activator was used relative to the phosphor amidite and all reactions were carried out for 30 minutes. After the phosphitylation reaction, all reactions were treated with CSO for 30 minutes to oxidize the phosphite intermediate to phosphotriester peptide **9**. The resin was then treated with DCM containing 10% TFA and 2.5% TIS for the duration of 120 minutes to cleave the peptide from the resin. From the crude reaction mixture, an LC-MS sample was prepared for analysis.

Scheme S1. Synthetic scheme towards arginine linked Ub^{ADPr}'s 18-21.

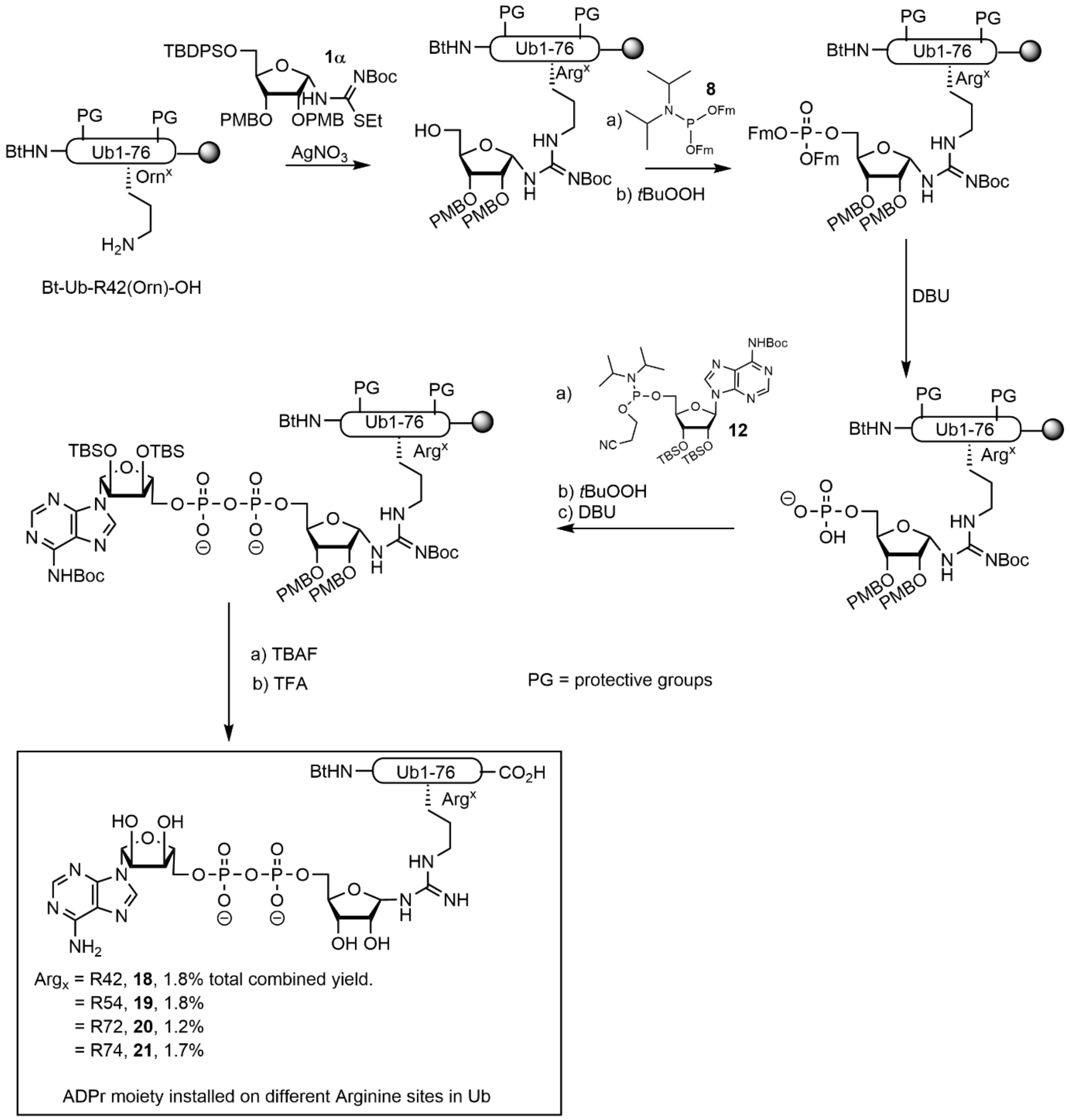

HRMS spectra of intermediates in the synthesis of ^{R42}Ub^{ADPr} (18)

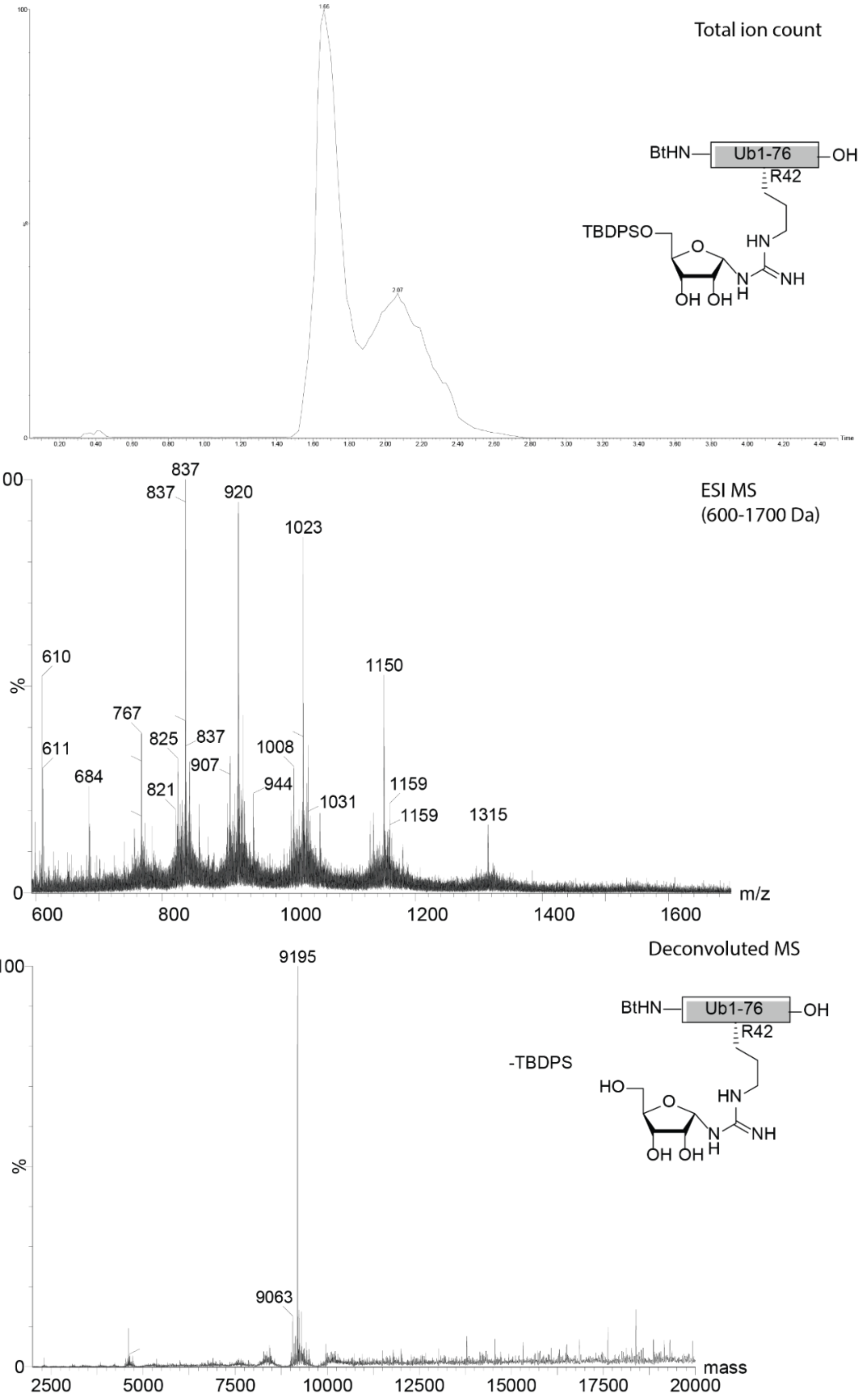

Figure S1. HRMS spectra of Ribosylated $Ub_{1\text{-}76}$ (R42 → NH_2 ornithine) using **1α**. During the test cleavage conditions: TFA/TIS/H_2O/Phenol (90.5/2/5/2.5), to release Ubiquitin from the resin the TBDPS group was deprotected (lower panel, deconvoluted mass = 9195).

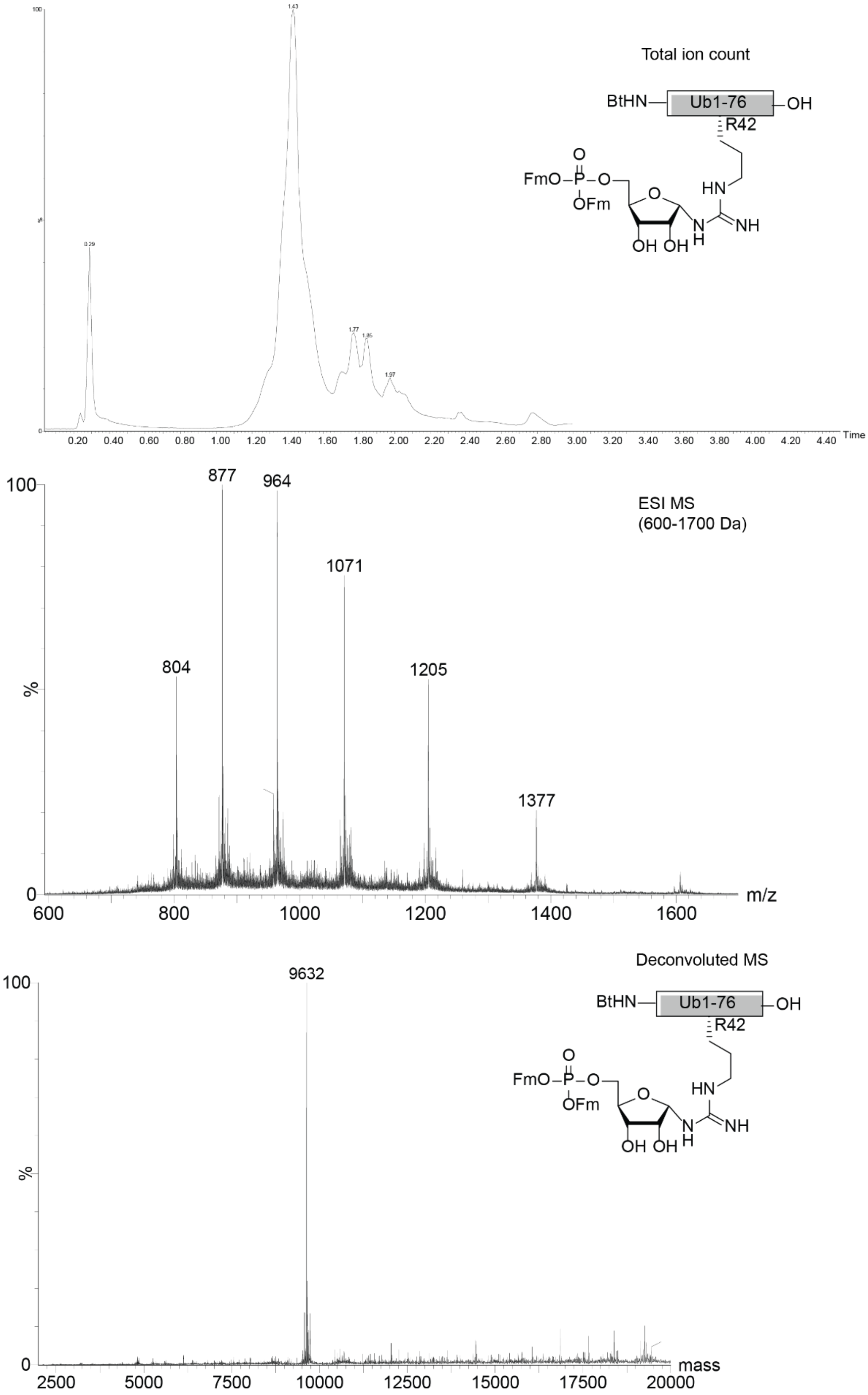

Figure S2. HRMS spectra of the phosphitylation reaction and subsequent oxidation of $Ub_{1\text{-}76}$ using phosphoramidite **8** (lower panel, deconvoluted mass = 9632).

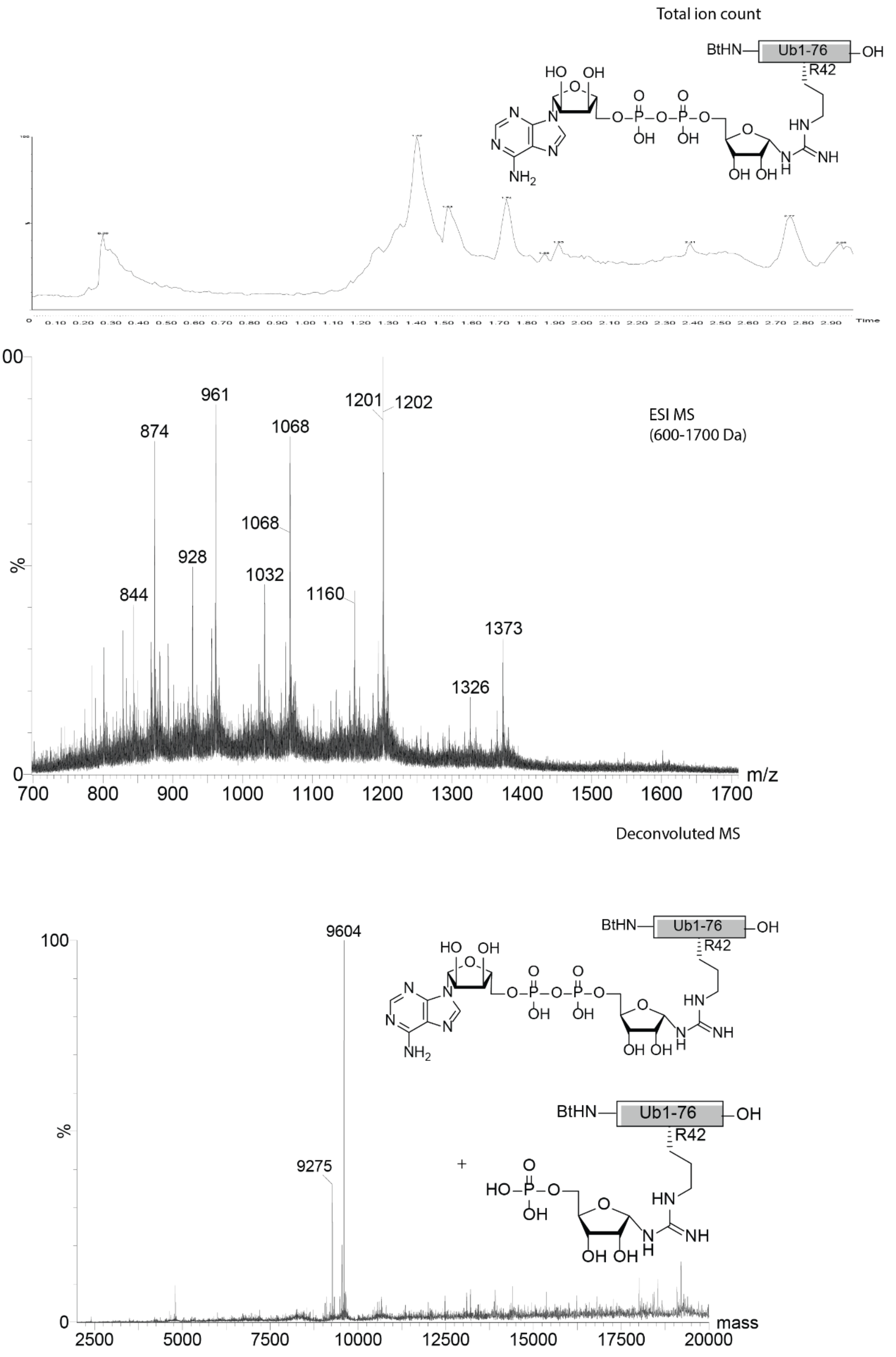

Figure S3. HRMS spectra of the ADPr formation reaction and subsequent oxidation of $Ub_{1\text{-}76}$ using nucleoside amidite **12**. The coupling reaction did not go to full conversion leaving uncoupled Ub^{Pr} (deconvoluted mass = 9275) in the mixture. $^{R42}Ub^{ADPr}$ (deconvoluted mass = 9604) was formed.

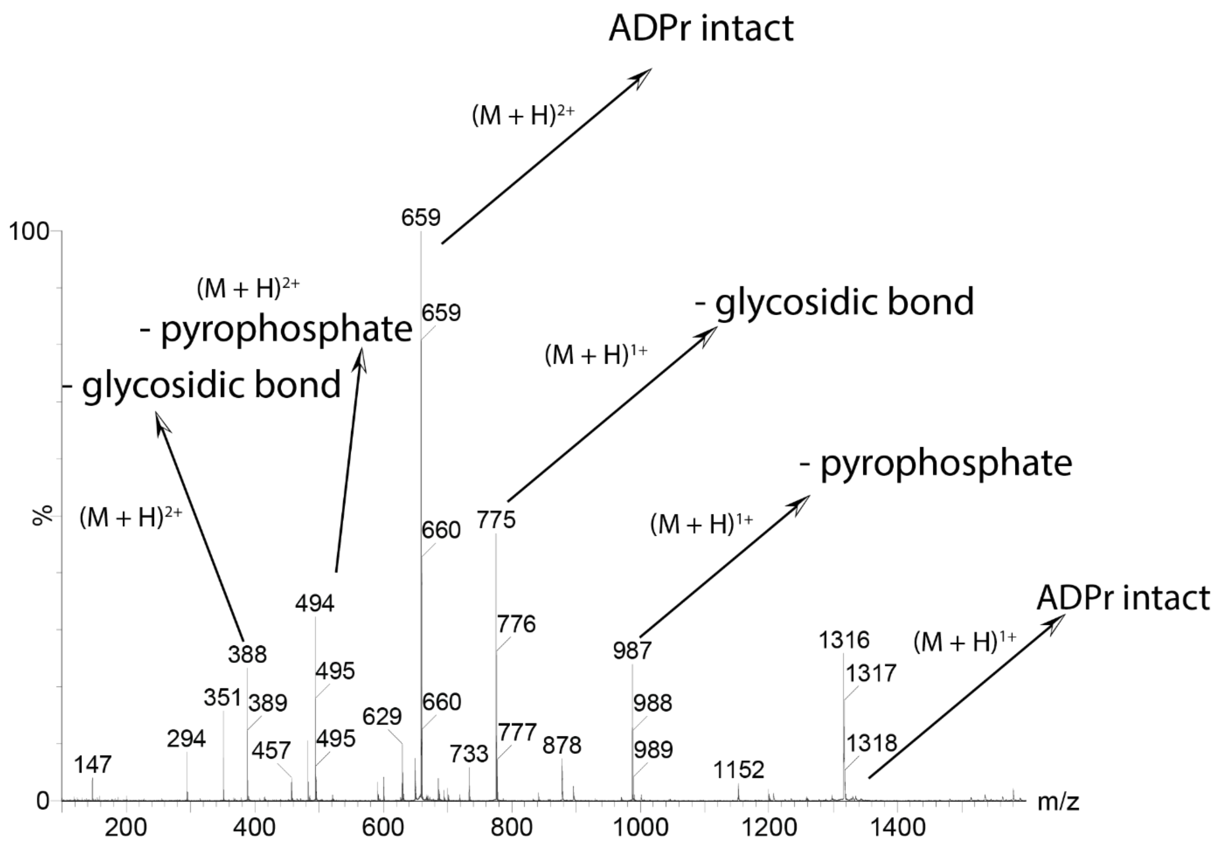

Figure S4. LC-MS of heptamer 14 treated for 90 min with TFA/TIS/H_2O/Phenol (90.5/2/5/2.5).

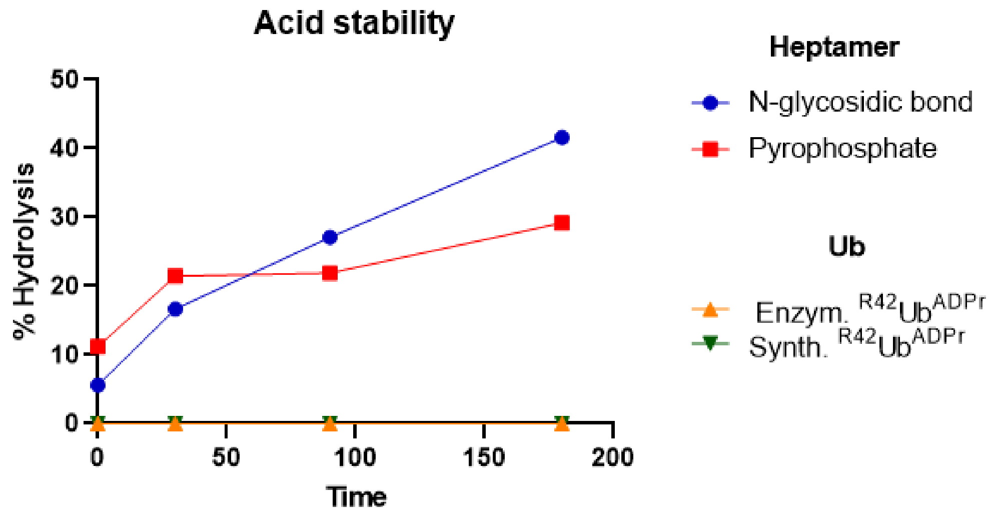

Figure S5. Acid stability of Arg-ADPr compounds. Heptapeptide **14** (1.66 µM), synth. $^{Arg42}Ub^{ADPr}$ **18** (1.66 µM), or enzym. $^{Arg42}Ub^{ADPr}$ (1.66 µM), was stirred in 100 µL TFA/TIS/H_2O/Phenol (90.5/2/5/2.5) and analyzed by LC-MS at the indicated time points. The glycosidic bond cleavage and pyrophosphate hydrolysis were determined as ratio of product versus starting material and plotted.

HRMS spectra of purified $^{R42}Ub^{ADPr}$(18)

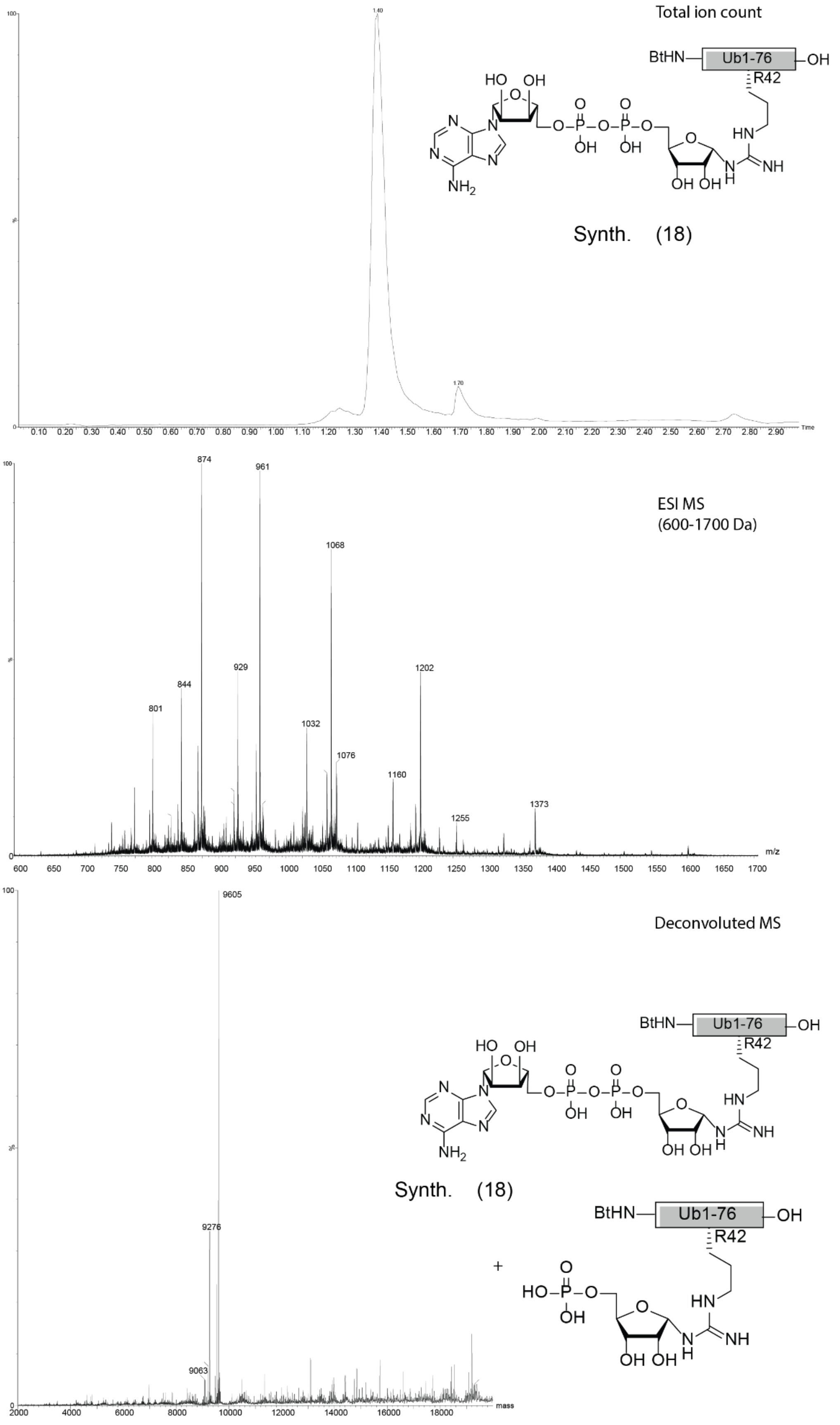

Figure S6. HRMS spectra of $^{R42}Ub^{ADPr}$ after purification.

HRMS spectra of purified $^{R54}Ub^{ADPr}$(19)

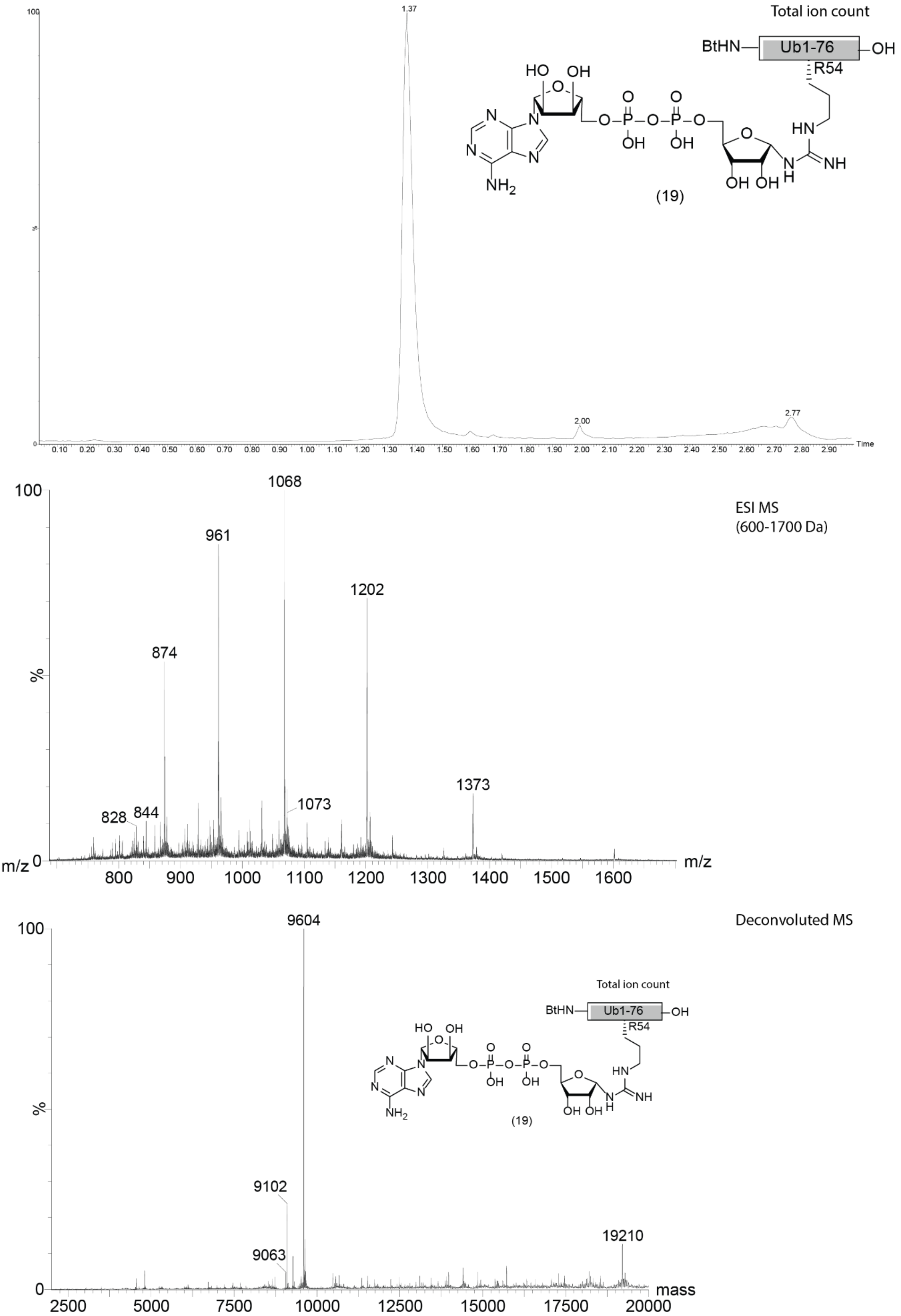

Figure S7. HRMS spectra of $^{R54}Ub^{ADPr}$ after purification.

HRMS spectra of purified $^{R72}Ub^{ADPr}$ (20)

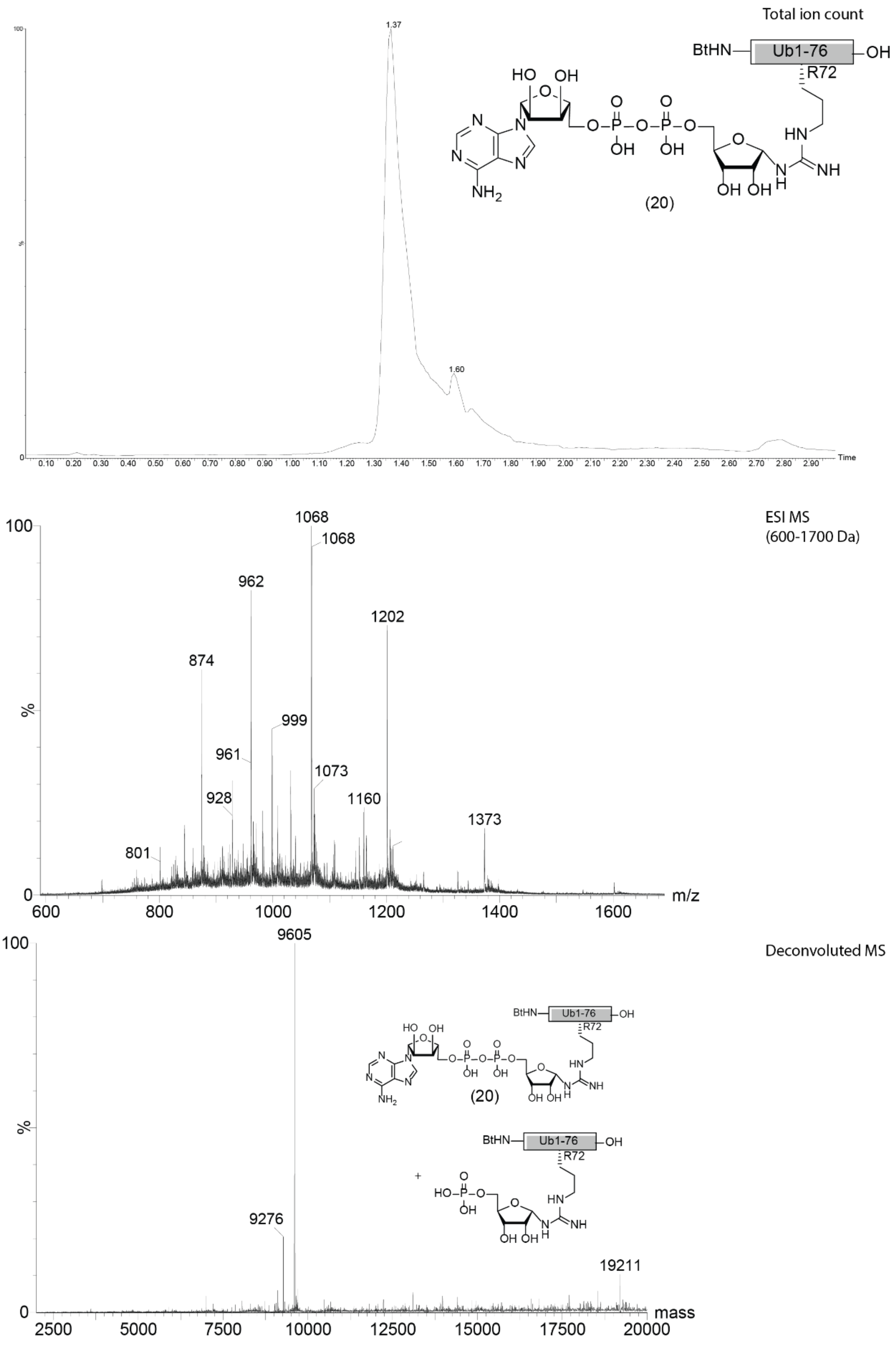

Figure S8. HRMS spectra of $^{R72}Ub^{ADPr}$ after purification.

HRMS spectra of purified $^{R74}Ub^{ADPr}$ (21)

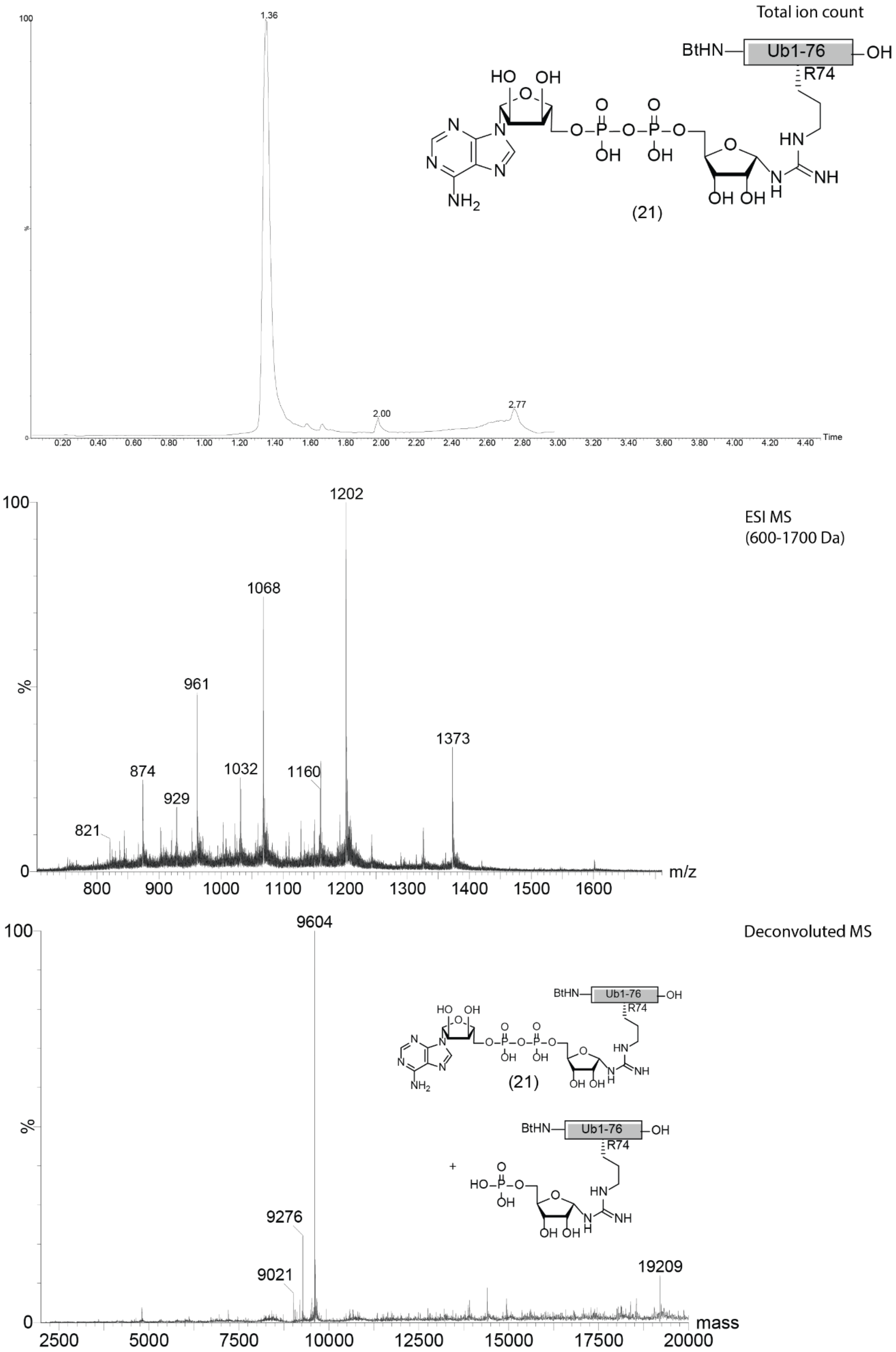

Figure S9. HRMS spectra of $^{R74}Ub^{ADPr}$ after purification.

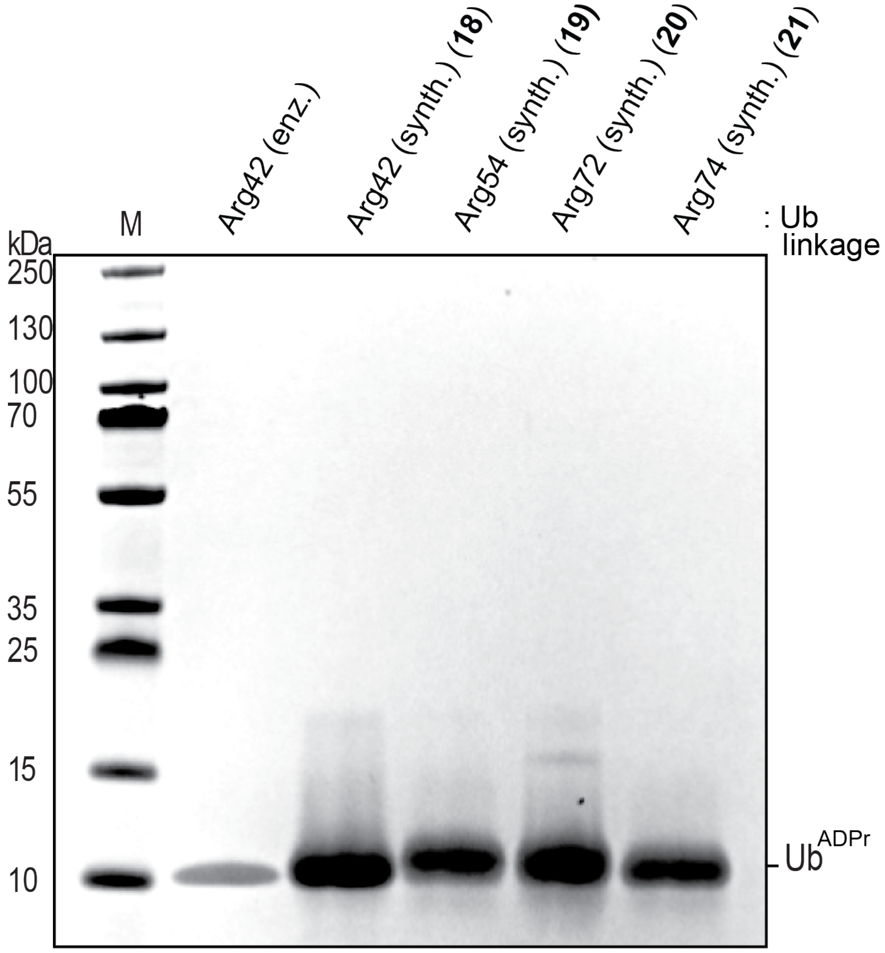

Figure S10. SDS-PAGE analysis of synthetic UbADPr's (**18**-**21**).

HRMS of enzymatically prepared $^{R42}Ub^{ADPr}$

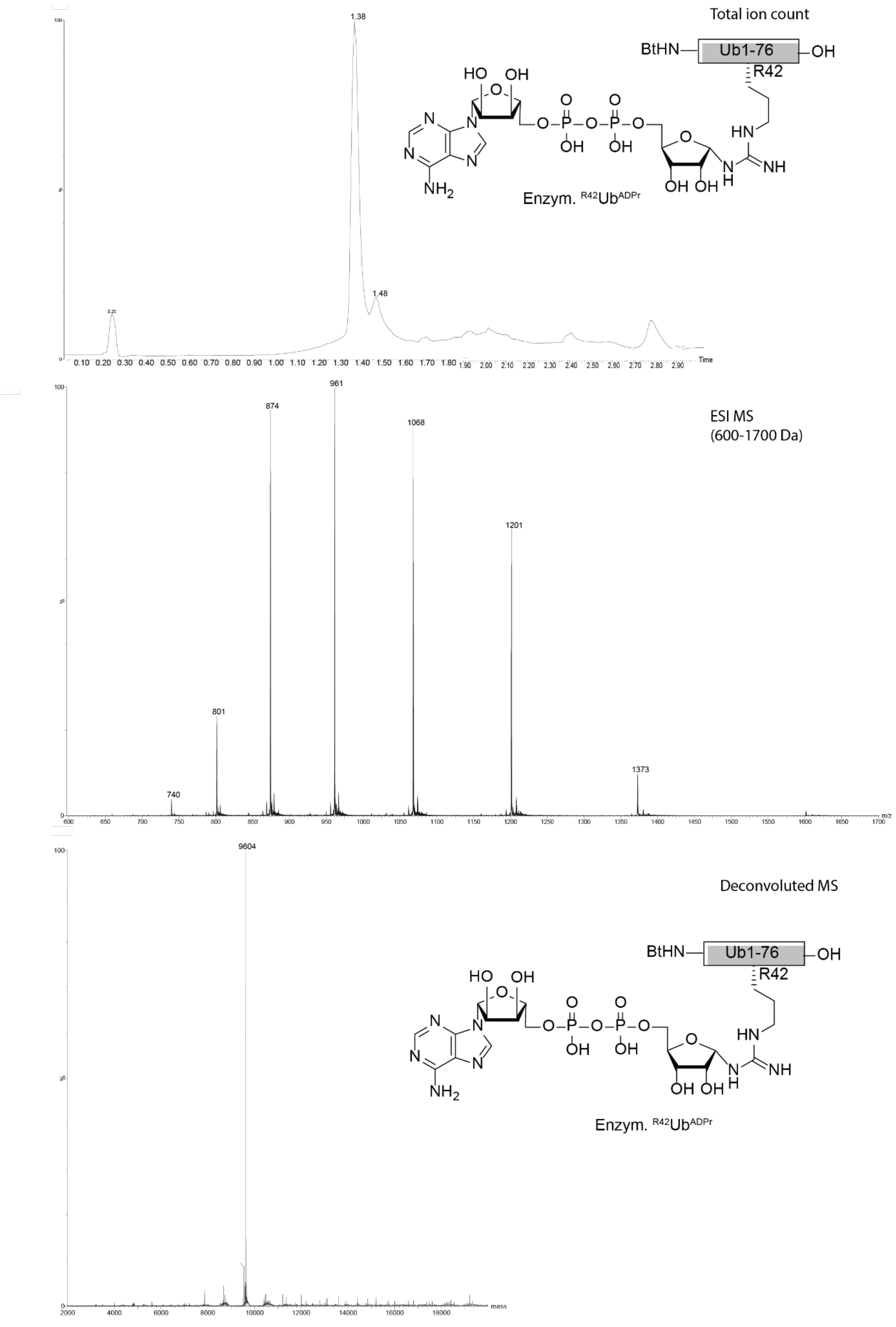

Figure S11. HRMS spectra enzymatically produced $^{R42}Ub^{ADPr}$.

HRMS of the DupA-mediated hydrolysis of enzymatically prepared $^{R42}Ub^{ADPr}$

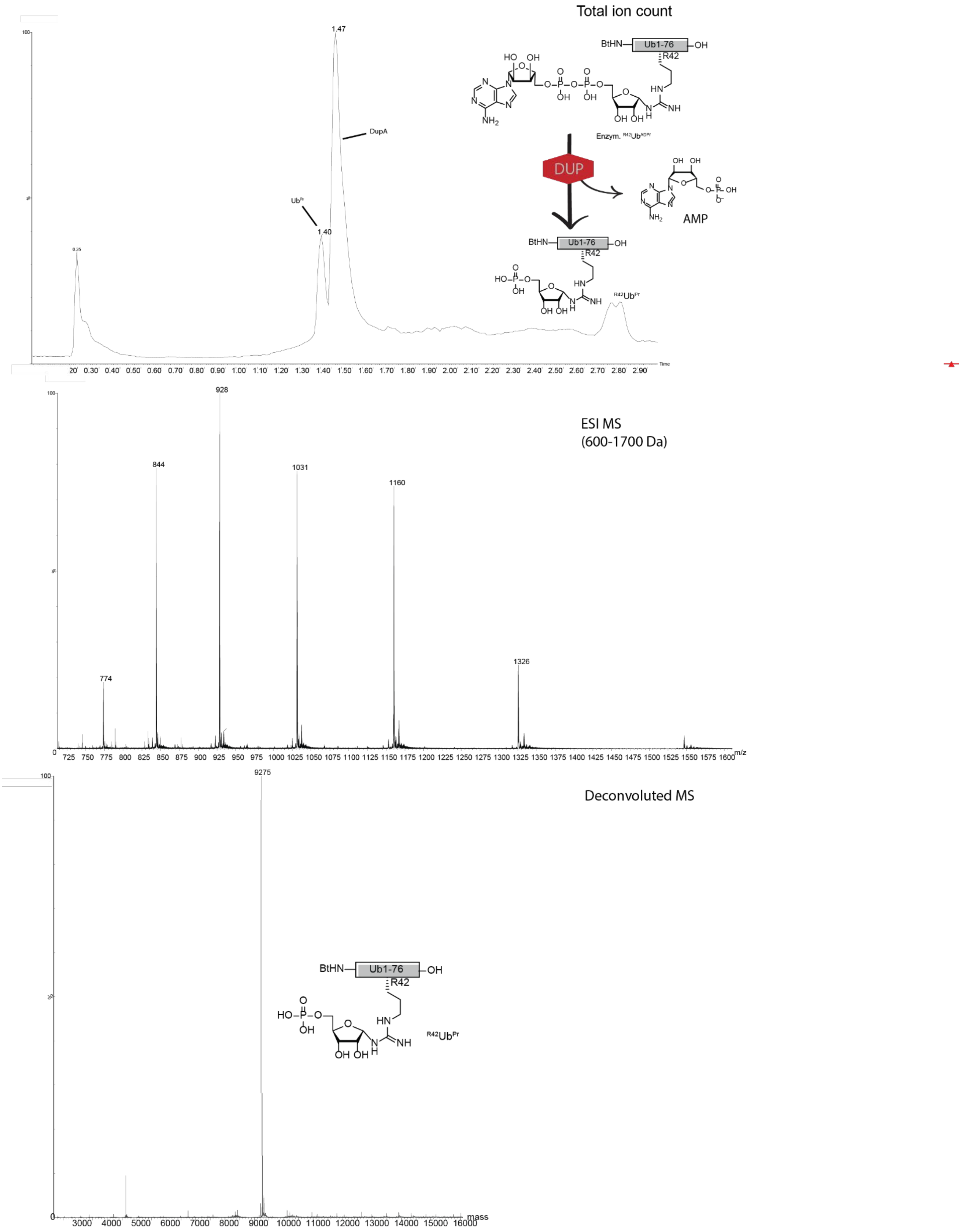

Figure S12. HRMS spectra of the DupA mediated hydrolysis reaction of enzymatically produced $^{R42}Ub^{ADPr}$ to form $^{R42}Ub^{Pr}$.

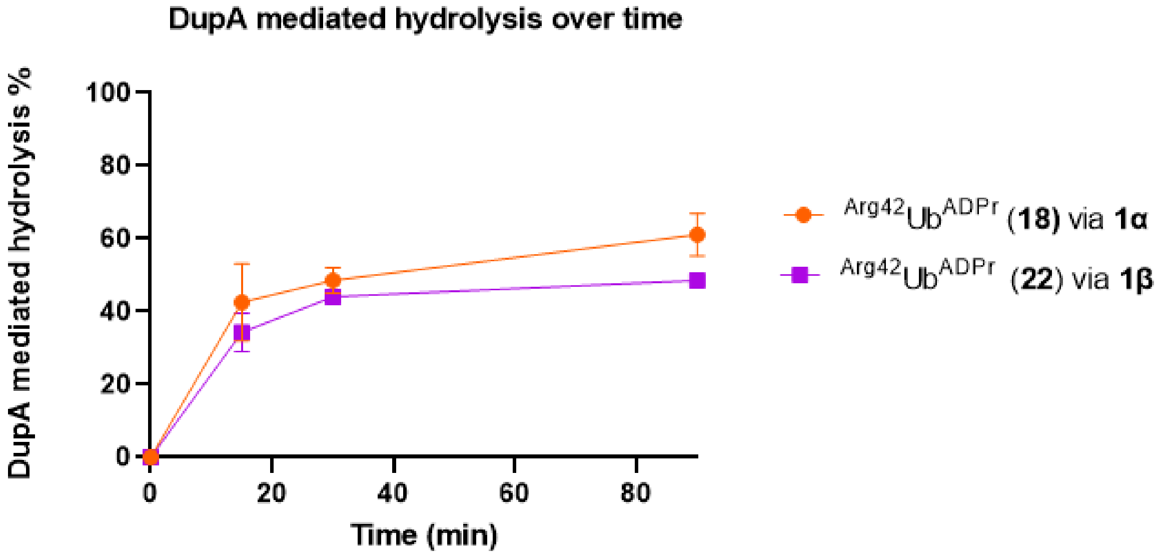

Figure S13. Hydrolysis of $^{Arg42}Ub^{ADPr}$ (**18**) synthesized via **1α** or $^{Arg42}Ub^{ADPr}$ (**22**) via **1β** by DupA followed over a time course of 0 - 90 min. Both graphs are analyzed with HRMS. The measurements in both graphs are normalized for background Ub^{Pr} present as impurity associated with the synthesis.

HRMS of the SdeA-mediated ligation of enzym. $^{R42}Ub^{ADPr}$ and RTN4b (23)

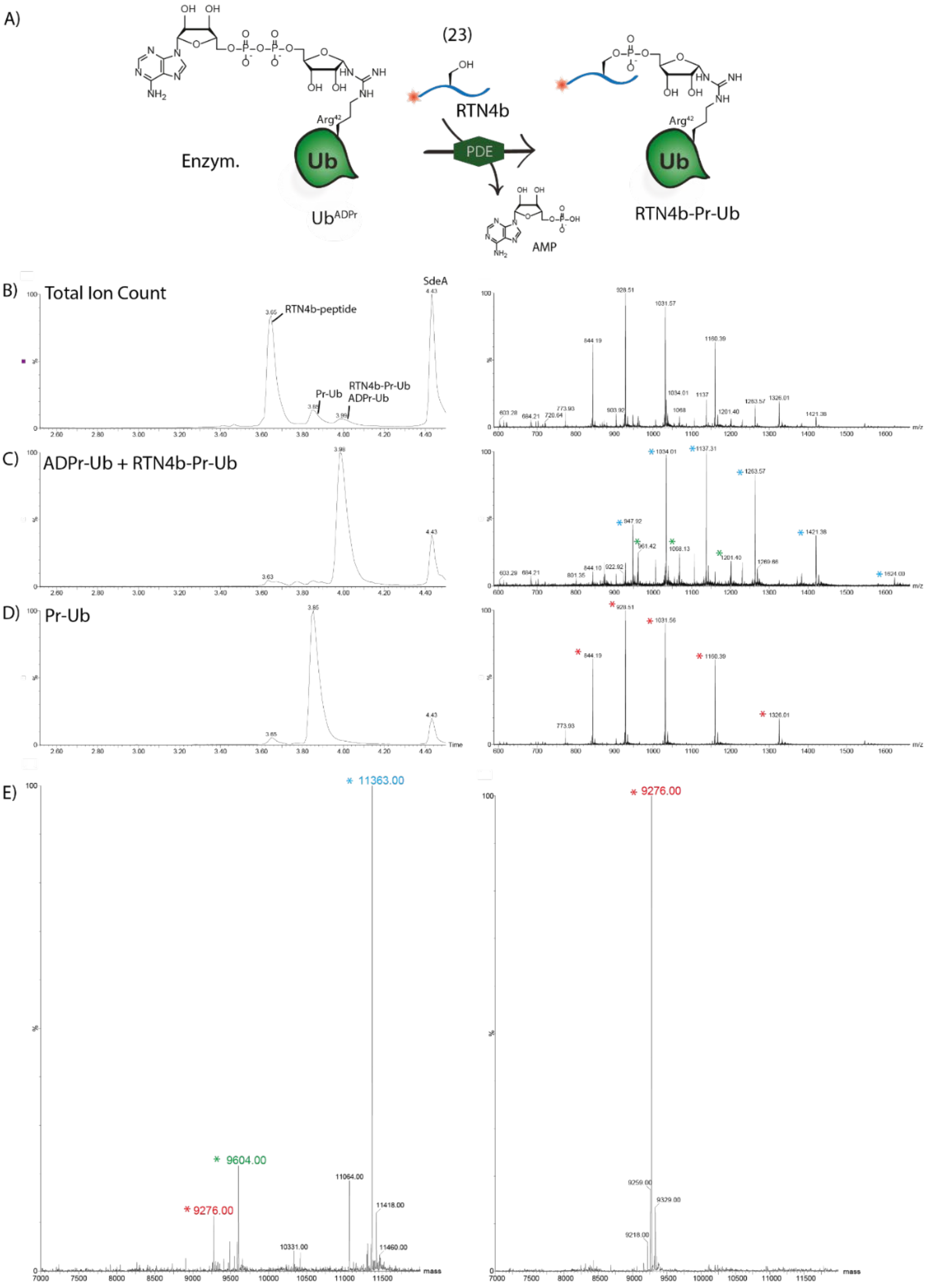

Figure S14. HRMS spectra of the SdeA mediated ligation reaction of enzymatically produced $^{R42}Ub^{ADPr}$ and RTN4b fragment (**23**). A) The SdeA mediated ligation of enzym. $^{R42}Ub^{ADPr}$ and fluorogenic RTN4b derived peptide (**23**). Ligation of serine in RTN4b to Ub^{ADPr} forms RTN4b-Pr-Ub as product. B) total ion count (left) and ESI-MS (right). ESI-MS corresponds to the total region of Pr-Ub, ADPr-Ub and RTN4b-Pr-Ub (retention time: 3.80-4.20). C) Total ion count (left) ADPr-Ub and RTN4b-Pr-Ub and corresponding ESI MS (right) (retention time 3.95-4.20). D) Total ion count (left) Pr-Ub and corresponding ESI MS (right) (retention time 3.80-3.95). E) Deconvoluted mass of C (left) and D (right).

HRMS of the SdeA-mediated ligation of synth. $^{R42}Ub^{ADPr}$ (18) and RTN4b (23)

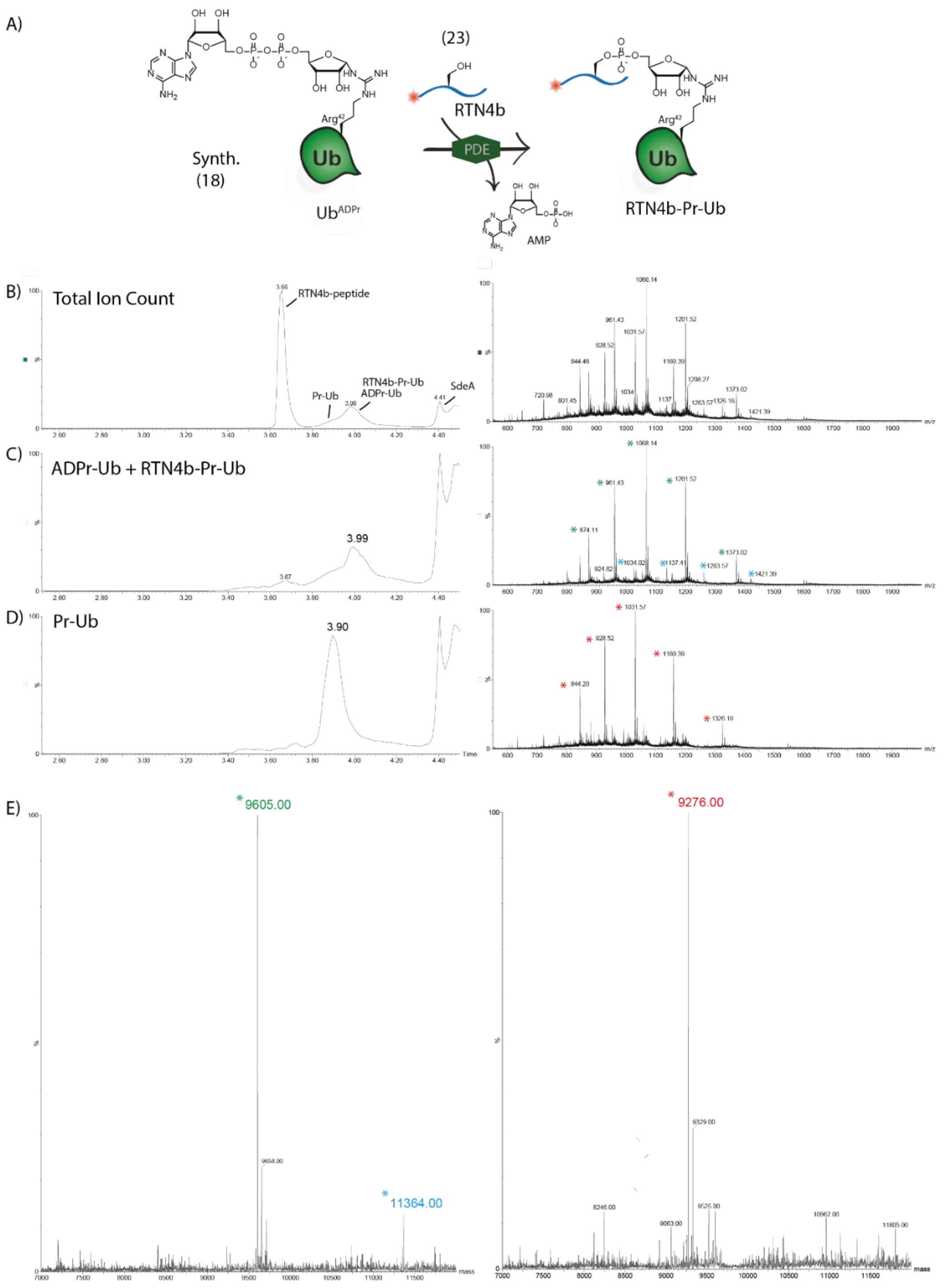

Figure S15. HRMS spectra of the SdeA mediated ligation reaction of synthesized $^{R42}Ub^{ADPr}$ (**18**) and RTN4b fragment (**23**). A) The SdeA mediated ligation of synth. $^{R42}Ub^{ADPr}$ (**18**) and fluorogenic RTN4b derived peptide (**23**). Ligation of serine in RTN4b to Ub^{ADPr} forms RTN4b-Pr-Ub as product. B) total ion count (left) and ESI-MS (right). ESI-MS corresponds to the total region of Pr-Ub, ADPr-Ub and RTN4b-Pr-Ub (retention time: 3.80-4.20). C) Total ion count (left) ADPr-Ub and RTN4b-Pr-Ub and corresponding ESI MS (right) (retention time 3.99-4.15). D) Total ion count (left) Pr-Ub and corresponding ESI MS (right) (retention time 3.80-3.95). E) Deconvoluted mass of C (left) and D (right).

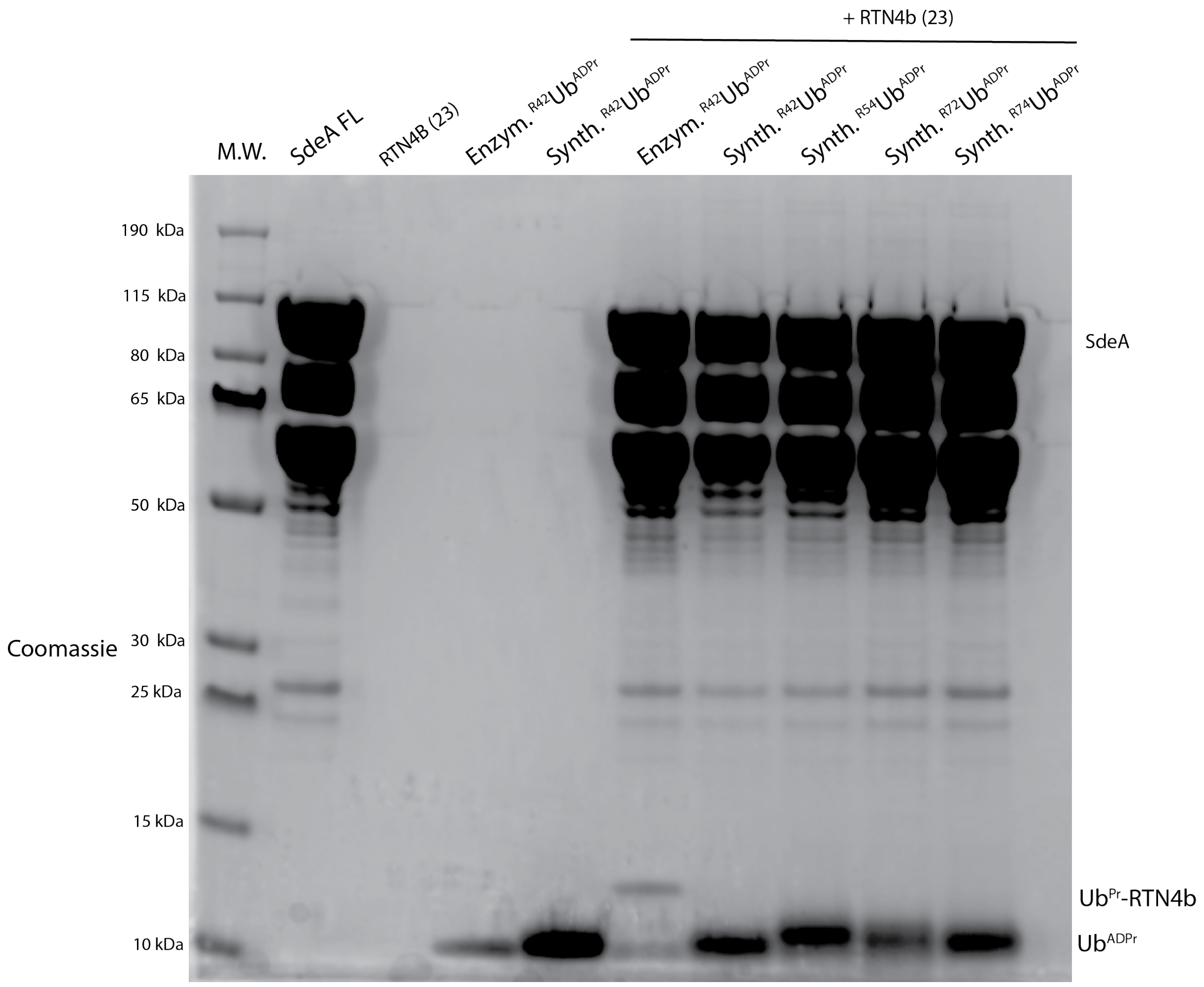

Figure S16. Uncropped SDS-PAGE gel of the SdeA-mediated ligation of enzym. $^{R42}Ub^{ADPr}$ or synth. $^{Rxx}Ub^{ADPr}$ **(18-21)** and RTN4B peptide fragment (**23**).

General synthetic procedures

All reagents were used as received unless stated otherwise. Solvents used in synthesis were dried and stored over 4Å molecular sieves, except for MeOH and MeCN which were stored over 3Å molecular sieves. Triethylamine (TEA) and diisopropylethylamine (DIPEA) were stored over KOH pellets. Column chromatography was performed on silica gel 60 Å (40-63 µm, Macherey-Nagel). TLC analysis was performed on Macherey-Nagel aluminium sheets (silica gel 60 F_{254}). TLC was used to visualize compounds by UV at wavelength 254 nm and by spraying with either cerium molybdate spray (25 g/L $(NH_4)_6Mo_7O_{24}$, 10 g/L $(NH_4)_4Ce(SO_4)_4{\cdot}H_2O$ in 10% H_2SO_4 water solution) or $KMnO_4$ spray (20 g/L $KMnO_4$ and 10 g/L K_2CO_3 in water) followed by charring at c.a. 250 °C. LC-MS analysis was performed on a Finnigan Surveyor HPLC system with a Nucleodur C18 Gravity 3 µm 50 x 4.60 mm column (detection at 200-600 nm) coupled to a Finnigan LCQ Advantage Max mass spectrometer with ESI or coupled to a Thermo LCQ Fleet Ion mass spectrometer with ESI. The method used was 10→90% 13.5 min (0→0.5 min: 10% MeCN; 0.5→8.5 min: 10% to 90% MeCN; 8.5→ 11 min: 90% MeCN; 11→13.5 min: 10% MeCN) or 0→50% 13.5 min. NMR spectra were recorded on a Bruker AV-400, AV-500 or AV-600 NMR. Chemical shifts (δ) are given in ppm relative to tetramethyl silane. Coupling constants (*J*) are given in Hz. All given ^{13}C-APT spectra are proton decoupled. In case of synthetic Ub-ADPr, HPLC purification was performed on a Shimadzu semi-preparative RP-HPLC system, equipped with a Waters C18-Xbridge 5 µm OBD (10 x 150 mm) column at a flowrate of 6.5 mL/min. using 2 mobile phases: A: MQ + 0.05% FA, B: MeCN + 0.05 % FA. Gradient: 10 -> 70% B. High resolution mass spectra were recorded on a Waters XEVO-G2 XS Q-TOF mass spectrometer equipped with an electrospray ion source in positive mode (source voltage 3.0 kV, desolvation gas flow 900 L/hr, temperature 250 °C) with resolution R = 22000 (mass range m/z = 50-2000) and 200 pg/uL Leu-Enk (m/z = 556.2771) as a “lock mass”.

2,3,5-tri-*O*-acetyl-β-D-ribofuranosyl azide

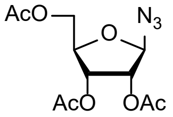

2,3,5-tri-*O*-acetyl-β-D-ribofuranosyl azide was prepared according to a modified, literature procedure.[31] Commercially available 1,2,3,5-tetra-*O*-acetyl-β-ribofuranose (7.96 g, 25 mmol) was dissolved in DCM (225 mL, 0.1 M). TMS-N_3 (3.65 mL, 27.5 mmol, 1.1 eq.) and a 1.0 M $SnCl_4$ solution in DCM (25 mL, 25 mmol, 1.0 eq.) were added to the reaction. The reaction was stirred for 2 hours after which TLC indicated full conversion. The reaction was carefully quenched with sat. aq. $NaHCO_3$ and transferred into a separatory funnel. The water layer was extracted with DCM and the combined organic layers were washed with brine, dried over $MgSO_4$, filtered and concentrated *in vacuo*. Flash column chromatography (30% EtOAc in pentane) furnished the title compound as a colorless oil in quantitative yield. Spectral data was in accordance with literature.[36] **Rf**: 0.43 (30% EtOAc in pentane). **^{1}H NMR:** (400 MHz, $CDCl_3$) δ 5.38 (d, *J* = 2.0 Hz, 1H, H-1), 5.34 (dd, *J* = 6.8, 4.8 Hz, 1H, H-3), 5.14 (dd, *J* = 4.8, 2.0 Hz, 1H, H-2), 4.42 (dd, *J* = 12.1, 3.2 Hz, 1H, H-5$_a$), 4.39 – 4.33 (m, 1H, H-4), 4.15 (dd, *J* = 12.1, 4.2 Hz, 1H, H-5$_b$), 2.13 (s, 6H, 2x Ac), 2.08 (s, 3H, Ac). **^{13}C NMR:** (101 MHz, $CDCl_3$) δ 170.5, 169.5, 169.4 (C=O Ac), 92.6 (C-1), 79.3 (C-4), 74.4 (C-2), 70.4 (C-3), 62.9 (C-5), 20.6, 20.5, 20.4 (CH_3 Ac).

5-*O*-*tert*-butyl-diphenylsilyl -β-D-ribofuranosyl azide (2)

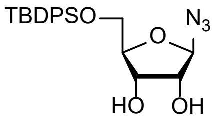

2,3,5-tri-*O*-acetyl-β-D-ribofuranosyl azide (2.99 g, 9.92 mmol was dissolved in MeOH (50 mL, 0.2 M). A 25 wt% solution of NaOMe in MeOH (0.23 mL, 0.99 mmol, 0.1 eq.) was added and the solution was stirred for 1 hour. The reaction was quenched with Amberlite H^+ resin, filtered and concentrated *in vacuo*. The resulting residue was co-evaporated extensively with pyridine and dissolved in pyridine (100 mL, 0.1 M). TBDPS-Cl (2.8 mL, 11 mmol, 1.1 eq.) was added and the reaction was stirred overnight. The reaction was quenched by the addition of MeOH and the reaction was concentrated *in vacuo*. The residue was taken up in EtOAc and the resulting solution was washed with 1 M HCl, sat. aq. $NaHCO_3$ and brine consecutively. The organic layer was dried over $MgSO_4$, filtered and concentrated *in vacuo*. Flash column chromatography (20 -> 40% EtOAc in pentane) yielded the title compound as a colorless oil (3.65 g, 8.83 mmol, 89%). **Rf:** 0.54 in 40% EtOAc in pentane. **^{1}H NMR:** (400 MHz, $CDCl_3$) δ 7.77 – 7.62 (m, 4H, TBDPS arom.), 7.51 – 7.32 (m, 6H, TBDPS arom.), 5.31 (d, J = 1.9 Hz, 1H, H-1), 4.36 (t, J = 6.3, 4.8 Hz, 1H, H-3), 4.05 (dt, J = 6.3, 4.2 Hz, 1H, H-4), 3.96 (dd, J = 4.8, 2.0 Hz, 1H, H-2), 3.83 (qd, J = 11.2, 4.2 Hz, 2H, H-5), 3.06 (bs, 1H, OH), 2.60 (bs, 1H, OH), 1.08 (s, 9H, *t*Bu TBDPS). **^{13}C NMR:** (101 MHz, $CDCl_3$) δ 135.7 (CH arom. TBDPS), 133.1, 133.0 (Cq TBDPS), 130.0, 130.0, 128.0, 127.9 (CH arom. TBDPS), 94.9 (C-1), 83.9 (C-4), 75.6 (C-2), 71.5 (C-3), 63.9 (C-5), 26.9 (CH_3 *t*Bu TBDPS), 19.3 (Cq *t*Bu TBDPS). **HRMS:** $[C_{21}H_{37}N_3O_4Si + Na]^+$ found: 436.1664, calculated: 436.1663.

5-*O*-((*tert*-butyl)-diphenylsilyl)-2,3-di-*O*-(4-methoxybenzyl)-β-D-ribofuranosyl azide (3)

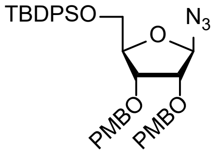

5-*O*-((*tert*-butyl)-diphenylsilyl)-β-D-ribofuranosyl azide **2** (3.65 g, 8.83 mmol) and TBABr (570 mg, 1.77 mmol, 0.2 eq.) were co-evaporated in toluene before they were dissolved in DMF (44 mL, 0.2 M). PMB-Cl (4.80 mL, 35.3 mmol, 4.0 eq.) was added and the solution was cooled to 0 °C. NaH (60 wt% dispersion in oil, 1.06 g, 26.5 mmol, 3.0 eq.) was added and the reaction was stirred for 15 minutes before the ice bath was removed. The reaction was stirred for an additional hour before TLC showed full conversion of the starting material. The reaction was cooled to 0 °C and carefully quenched by the addition of sat. aq. $NaHCO_3$. After bubbling seized, the emulsion was transferred into a separatory funnel and diluted with sat. aq. $NaHCO_3$. The water layer was extracted thrice with Et_2O and the combined organic layers were dried over $MgSO_4$, filtered and concentrated *in vacuo*. Flash column chromatography (20 -> 30% Et_2O in pentane) furnished the title compound (3.91 g, 5.98 mmol, 68%) as a clear oil. **Rf:** 0.56 in 30% Et_2O in pentane. **^{1}H NMR:** (400 MHz, $CDCl_3$) δ 7.67 (ddt, J = 8.1, 6.5, 1.6 Hz, 4H, TBDPS arom.), 7.45 – 7.33 (m, 6H, TBDPS arom.), 7.32 – 7.25 (m, 2H, PMB arom.), 7.22 – 7.14 (m, 2H, PMB arom.), 6.92 – 6.78 (m, 4H, PMB arom.), 5.37 (d, J = 2.2 Hz, 1H, H-1), 4.55 (q, J = 11.8 Hz, 2H, CH_2 PMB), 4.49 – 4.34 (m, 2H, CH_2 PMB), 4.22 (dt, J = 6.6, 3.3 Hz, 1H, H-4), 4.16 (dd, J = 6.7, 4.5 Hz, 1H, H-3), 3.84 (dd, J = 11.5, 3.1 Hz, 1H, H-5_a), 3.78 (s, 3H, CH_3 PMB), 3.77 (s, 3H, CH_3 PMB), 3.73 (dd, J = 4.6, 2.2 Hz, 1H, H-2), 3.69 (dd, J = 11.5, 3.5 Hz, 1H, H-5_b), 1.04 (s, 9H, CH_3, *t*Bu TBDPS). **^{13}C NMR:** (101 MHz, CDCl3) δ 159.5, 159.4 (Cq PMB), 135.7, 135.7, 134.9 (CH arom. TBDPS), 133.2, 133.1 (Cq TBDPS), 129.8, 129.8, 129.8 (CH arom. TBDPS/PMB), 129.7 (Cq PMB), 129.7, 129.6 (CH arom. TBDPS/PMB), 129.5 (Cq PMB), 127.8, 127.8, 114.0, 113.9, 113.8 (CH arom. PMB), 93.2 (C-1), 83.0 (C-4), 79.7 (C-2), 76.0 (C-3), 72.2, 72.1 (CH_2 PMB), 63.1 (C-5), 55.3, 55.3 (CH_3 PMB), 26.8 (CH_3 TBDPS), 19.3 (Cq *t*Bu TBDPS). **HRMS:** $[C_{37}H_{43}N_3O_6Si + Na]^+$ found: 676.2812, calculated: 676.2813.

5-*O*-((*tert*-butyl)-diphenylsilyl)-2,3-di-*O*-(4-methoxybenzyl)-α,β-D-ribofuranosyl isothiocyanate (4)

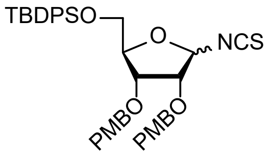

5-*O*-((*tert*-butyl)-diphenylsilyl)-2,3-di-*O*-(4-methoxybenzyl)-β-D-ribofuranosyl azide **3** (1.34 g, 2.04 mmol) was dissolved in EtOAc (20 mL. 0.1 M) and the reaction was purged with nitrogen whilst sonicating for 10 minutes. To the stirred solution PtO_2 (93 mg, 0.41 mmol, 0.2 eq.) was added. The solution was purged with H_2 for 2 hours after which the reaction was filtered over a pad of Celite. To the filtrate water was added (20 mL) followed by K_2CO_3 (11.3 g, 8.16 mmol, 4.0 eq.) and thiophosgene (313 µL, 4.08 mmol, 2.0 eq.). The suspension was vigorously stirred overnight after which it was transferred into a separatory funnel and sat. aq. $NaHCO_3$ was added. The water layer was extracted thrice with EtOAc and the combined organic layers were dried over $MgSO_4$, filtered and concentrated *in vacuo*. Flash column chromatography (10 -> 15% Et_2O in pentane) yielded the title compound as a colorless oil (α-anomer 670 mg, 1.00 mmol. β-anomer 378 mg, 0.56 mmol. Combined total yield of 76%). *α-anomer:* **Rf:** 0.36 in 20% Et_2O in pentane. **^{1}H NMR:** (400 MHz, CDCl3) δ 7.57 (ddt, J = 8.3, 6.6, 1.5 Hz, 4H, TBDPS arom.), 7.47 – 7.33 (m, 6H, TBDPS arom.), 7.33 – 7.26 (m, 4H, PMB arom.), 6.90 – 6.81 (m, 4H, PMB arom.), 5.31 (d, J = 4.5 Hz, 1H, H-1), 4.74 (d, J = 11.7 Hz, 1H, CH_{2a} PMB), 4.61 (s, 2H, CH_2 PMB), 4.49 (d, J = 11.7 Hz, 1H, CH_{2b} PMB), 4.25 (q, J = 2.7 Hz, 1H, H-4), 4.09 – 4.00 (m, 2H, H-2 + H-3), 3.79 (s, 3H, CH_3 PMB), 3.78 (s, 3H, CH_3 PMB), 3.64 (dd, J = 11.5, 3.3 Hz, 1H, H-5_a), 3.55 (dd, J = 11.4, 2.8 Hz, 1H, H-5_b), 0.97 (s, 9H, *t*Bu TBPDS). **^{13}C NMR:** (101 MHz, $CDCl_3$) δ 159.6, 159.3 (Cq PMB), 139.9 (NCS), 135.6, 135.6 (CH arom. TBDPS), 133.0, 132.8 (Cq TBDPS), 130.2 (Cq PMB), 130.0, 130.0, 129.7, 129.6 (CH arom TBDPS/PMB), 129.2 (Cq PMB), 127.9, 127.9, 114.1, 113.8 (CH arom. PMB), 86.1 (C-1), 85.7 (C-4), 79.6, 75.5 (C-2 + C-3), 72.8, 72.6 (CH_2 PMB), 63.7 (C-5), 55.3 (CH_3 PMB), 26.9 (CH_3 TBPDS), 19.3 (Cq *t*Bu TBDPS). *β-anomer:* **Rf:** 0.27 in 20% Et_2O in pentane. **^{1}H NMR:** (400 MHz, $CDCl_3$) δ 7.66 (ddt, J = 8.0, 6.3, 1.7 Hz, 4H, TBDPS arom.), 7.45 – 7.33 (m, 6H, TBDPS arom.), 7.30 – 7.16 (m, 4H, PMB arom.), 6.92 – 6.79 (m, 4H, PMB arom.), 5.42 (d, J = 2.7 Hz, 1H, H-1), 4.55 (s, 2H, CH_2 PMB), 4.44 (q, J = 11.4 Hz, 2H, CH_2 PMB), 4.20 – 4.11 (m, 2H, H-3 + H-4), 3.96 (dd, J = 4.2, 2.8 Hz, 1H, H-2), 3.80 (dd, J = 11.5, 2.8 Hz, 1H, H-5_a), 3.77 (s, 3H, CH_3 PMB), 3.76 (s, 3H, CH_3 PMB), 3.68 (dd, J = 11.6, 3.2 Hz, 1H, H-5_b), 1.04 (s, 9H, *t*Bu TBDPS). **^{13}C NMR:** (101 MHz, $CDCl_3$) δ 159.6, 159.5 (Cq PMB), 140.7 (NCS), 135.7, 135.6 (CH arom. TBDPS), 133.2, 132.9 (Cq TBDPS), 129.8, 129.8, 129.6 (CH arom. TBDPS/PMB), 129.5, 129.1 (Cq PMB), 127.9, 127.8, 127.8 (CH arom. PMB), 114.1, 114.0, 113.9 (CH arom. PMB), 88.4 (C-1), 83.4 (C-4), 81.3 (C-2), 76.0 (C-3), 72.5, 72.3 (CH_2 PMB), 63.2 (C-5), 55.3, 55.3 (CH_3 PMB), 26.9 (CH_3 TBDPS), 19.3 (Cq *t*Bu TBDPS). **HRMS:** $[C_{38}H_{43}NO_6Si + Na]^+$ found: 692.2464, calculated: 692.2473.

1-(*tert*-butoxycarbonyl)-3-(5-*O*-((*tert*-butyl)-diphenylsilyl)-2,3-di-*O*-(4-methoxybenzyl)-α-D-ribofuranos-1-yl)-2-ethylisothiourea (1α)

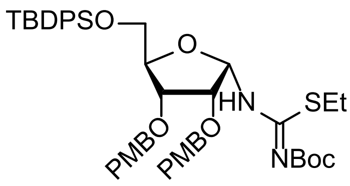

5-*O*-((*tert*-butyl)-diphenylsilyl)-2,3-di-*O*-(4-methoxybenzyl)-α-D-ribofuranosyl isothiocyanate **4α** (alpha anomer only, 4.08 g, 6.10 mmol) was dissolved in THF (31 mL, 0.2 M). The solution was purged with NH_3 for 1 hour after which the reaction was purged with N_2 for 1 minute. The crude thiourea was concentrated *in vacuo* till a white foam. The crude product was dissolved in DCM (61 mL, 0.1 M) and activated mol sieves were added. DMAP (75 mg, 0.61 mmol, 0.1 eq.) and Boc_2O (1.5 mL, 6.53 mmol, 1.1 eq.) were added and the reaction was stirred for 2 hours. The reaction was diluted with DCM and the resulting solution was washed with brine. The organic layer was dried over $MgSO_4$, filtered and concentrated *in vacuo*. The crude product was dissolved in MeCN (61 mL, 0.1 M) and K_2CO_3 (8.45 g, 69.1 mmol, 11.3 eq.) and EtI (1.75 mL, 21.88 mmol, 3.6 eq.) were added to the solution. The suspension was stirred overnight and taken up in EtOAc. The organic layer was washed with brine, dried over $MgSO_4$, filtered and concentrated *in vacuo*. Flash column chromatography (0 -> 45% EtOAc in heptane) furnished the title compound as a white foam (3.16 g, 3.88 mmol, 63.6%). **Rf:** 0.42 in 30% Et_2O in pentane. **^{1}H NMR:** (500 MHz, $CDCl_3$) δ 7.67 – 7.57 (m, 4H, TBDPS arom.), 7.43 – 7.39 (m, 2H, TBDPS arom.), 7.39 – 7.33 (m, 4H, TBDPS arom.), 7.30 – 7.23 (m, 4H, PMB arom.), 6.86 – 6.79 (m, 4H, PMB arom.), 5.68 (bs, 1H, H-1), 4.65 (d, J = 11.3 Hz, 1H, CH_{2a} PMB), 4.56 – 4.45 (m, 3H, CH_2 PMB + CH_{2b} PMB), 4.18 (ddd, J = 4.4, 3.3, 2.4 Hz, 1H, H-4), 4.12 (dd, J = 5.0, 2.5 Hz, 1H, H-3), 4.09 (t, J = 5.1 Hz, 1H, H-2), 3.77 (s, 3H, CH_3 PMB), 3.76 (s, 3H, CH_3 PMB), 3.70 – 3.62 (m, 2H, H-5), 2.90 (bs, 2H, CH_2 Et), 1.49 (s, 9H, CH_3 Boc), 1.21 (t, J = 7.4 Hz, 3H, CH_3 Et), 1.03 (s, 9H, CH_3 TBDPS). **^{13}C NMR:** (126 MHz, $CDCl_3$) δ 161.1 (C=O Boc), 159.7, 159.5 (Cq PMB), 135.7, 135.6 (CH arom. TBDPS), 133.4, 133.3 (Cq TBDPS), 130.3 (Cq PMB), 129.9, 129.9 (CH arom. TBDPS/PMB), 129.8 (Cq PMB), 129.6 (CH arom. TBDPS/PMB), 127.8, 127.8, 114.1, 114.0 (CH arom. PMB), 83.0 (C-4), 82.1 (C-1), 79.1 (Cq *t*Bu Boc), 78.2 (C-3), 77.4 (C-2), 73.0, 72.6 (CH_2 PMB), 64.1 (C-5), 55.3 (CH_3 PMB), 28.3 (*t*Bu CH_3 Boc), 27.0 (*t*Bu CH_3 TBDPS), 25.2 (CH_2 Et), 19.3 (Cq *t*Bu TBDPS), 13.8 (CH_3 Et). **HRMS:** $[C_{45}H_{58}N_2O_8SSi + H]^+$ found: 815.3745, calculated: 815.3756.

1-(*tert*-butoxycarbonyl)-3-(5-*O*-((*tert*-butyl) diphenylsilyl)-2,3-di-*O*-(4-methoxybenzyl)-β-D-ribofuranos-1-yl)-2-ethylisothiourea (1β)

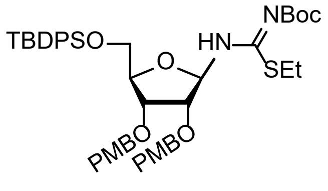

1-(*tert*-butoxycarbonyl)-3-(5-*O*-((*tert*-butyl) diphenylsilyl)-2,3-di-*O*-(4-methoxybenzyl)-β-D-ribofuranos-1-yl) isothiocyanate **4β** (beta anomer only, 2.36 g, 3.52 mmol) was dissolved in THF (18 mL, 0,2M). The solution was purged with NH_3 for 1 hour after which the reaction was purged with N_2 for 1 minute. The crude thiourea was concentrated *in vacuo* till a yellow foam. The crude product was dissolved in DCM (35 mL, 0.1M). DMAP (46.6 mg, 0.38 mmol, 0.11 eq) and Boc_2O (560 µL, 2.43 mmol, 1.1 eq) were added and the reaction was stirred for 2 hours. The reaction was diluted with DCM (100 mL) and washed with brine (100 mL). The organic layer was dried over $MgSO_4$, filtered and concentrated *in vacuo*. The crude product was dissolved in MeCN (61 mL, 0.1M) and K_2CO_3 (8.45, 69.1 mmol, 11.3 eq) and EtI (1.75 mL, 21.88 mmol, 3.6 eq) were added under vigorous stirring. The suspension was stirred overnight and diluted in EtOAc (100 mL). The organic layer was washed with brine, dried over $MgSO_4$, filtered and concentrated *in vacuo*.

Flash column chromatography (0 -> 45% EtOAC in heptane) obtained the title compound as a colorless oil (353 mg, 0.43 mmol, 12.3%). **Rf:** 0.45 in 30% Et_2O in pentane. **^{1}H-NMR (300 MHz, $CDCl_3$):** δ 7.67-7.59 (m, 4H, TBDPS arom.), 7.46-7.31 (m, 6H, TBDPS arom.), 7.29-7.18 (m, 4H, PMB arom.), 6.88-6.80 (m, 4H, PMB arom.), 5.58 (t, J = 6.4 Hz, 1H, H-1), 4.59 (s, 2H, CH_{2a} PMB), 4.54-4.41 (m, 2H, CH_{2b} PMB), 4.13 (q, J = 3.6 Hz, 1H, H-4), 4.02 (t, J = 6.0 Hz, 1H, H-3), 3.89 (t, J = 6.0 Hz, 1H, H-2), 3.80 (s, 3H, CH_3 PMB), 3.79 (s, 3H, CH_3 PMB), 3.75-3.58 (m, 2H, H-5), 3.04 (d, J = 7.4, 2H, CH_2Et), 1.48 (s, 9H, CH_3 Boc), 1.26 (t, J = 7.5 Hz, 3H, CH_3 Et), 1.01 (s, 9H, CH_3 TBDPS). **^{13}C-NMR (75,5 MHz, $CDCl_3$):** δ 161.7 (C=O Boc), 159.5, 159.5 (Cq PMB), 135.8, 135.7 (CH arom. TBDPS), 133.4, 133.0 (Cq TBDPS), 129.9, 129.9, 129.7, 129.7 (CH arom. TBDPS/PMB), 127.9, 127.9, 114.0, 114.0 (CH arom. PMB), 86.3 (C-4), 82.8 (C-1), 80.7(C-3), 79.6 (Cq *t*Bu Boc), 76.0 (C-2), 72.1, 71.9 (CH_2 PMB), 63.7 (C-3), 55.4, 55.4 (CH_3 PMB), 28.30 (CH_3 Boc), 27.1 (CH_3 TBDPS), 25.4 (CH_2 Et), 19.4 (Cq *t*Bu TBDPS), 13.9 (CH_3 Et). **HRMS:** $[C_{45}H_{58}N_2O_8SSi + H]^+$ found: 815.3863, calculated: 815.3756.

Synthesis of peptides 14-17 (general procedure synthesis Arg-ADPr)

Peptide synthesis (<u>Protocol A</u>, peptides **14**, **15**, **17**)
The intermediate peptides (generalized as **5**, Scheme 1) were synthesized using standard, Fmoc-based solid phase peptide synthesis utilizing (pre-loaded) Tentagel® S AC purchased from Rapp Polymer GmbH on a Syro II MultiSyntech Automated or a CEM Liberty Blue Automated Microwave Peptide Synthesizer Peptide synthesizer. Coupling cycles were as followed: Fmoc deprotection: 2x2 min, 1x5 min treatment with 20% piperidine in DMF. Coupling: treatment of 6 eq. amino acid, 6 eq. HCTU (0.25M in DMF) and 12 eq. DIPEA (1 M in DMF) for 30 minutes. Capping: 2x2 min treatment of the resin with a 10% Ac_2O solution in DMF and catalytic DIPEA. Washing between the steps was done with DMF. For the prospected Arg-ADPr site, commercially available Fmoc-Orn(OAll)-OH was used in the coupling cycle.

Peptide synthesis (<u>Protocol B</u>, peptide **16**)
The intermediate peptides (generalized as **5**, Scheme 1) were synthesized on a CEM Liberty Blue Automated Microwave Peptide Synthesizer. The resin was first swollen for 5 minutes in DMF prior to amino acid coupling. Activation was achieved using DIC/Oxyma. Standard coupling was achieved using 5 eq. amino acid as a 0.2 M amino acid/DMF solution, 5 eq. DIC as a 0.5 M of DIC/DMF solution and 5 eq. Oxyma as a 1M Oxyma/DMF solution which was buffered by DIPEA (0.1M) at 90˚C for 2 minutes. Standard Fmoc deprotection was achieved by 20% $^v/_v$ piperidine/DMF at 90˚C for 1 minute (2 cycles). Washing between the steps was done with DMF. For the prospected Arg-ADPr site, commercially available Fmoc-Orn(OAll)-OH was used in the coupling cycle. Synthesis quality could be monitored by UV absorption of dibenzofulvene released during Fmoc deprotection.

Deprotection/building block coupling for Arg-ADPr peptides
The Alloc protecting group was removed by treating the resin with a freshly prepared solution of 10 mg $Pd(PPh_3)_4$ and 23 mg 1,3-dimethylbarbituric acid in 1 mL DCM (purged with nitrogen prior to use) for 15 minutes. This procedure was then repeated twice to ensure full deprotection. The resin was washed extensively with DCM and DMF. Coupling of the ribosyl building block was performed as follows: Ribosyl building block **1α** (or **1β**) (3 eq.) was dissolved in DMF (0.1 M) and added to the resin. TEA (30 eq.) followed by $AgNO_3$ (3 eq.) were

added to the reaction and the syringe was wrapped in aluminum foil to protect it from light and shaken overnight. The resin was then extensively washed with DCM and DMF.

Deprotection and phosphorylation
The resin was washed with THF and treated with TBAF (1 M) in THF for 30 minutes. The resin was thoroughly washed with DCM and DMF before the treatment was repeated once, furnishing the desilylated intermediate. The resin was then extensively washed with dry MeCN and flushed with nitrogen to remove traces of water before the resin was subjected to a solution of $(FmO)_2PN(iPr)_2$ **8** (2.5 eq., (0.13 M in MeCN)) and DCI (5.0 eq. (0.25M in MeCN) was added. The resin was shaken for 30 minutes after which the resin was washed with MeCN. The resin was then treated with a 0.5 M CSO solution in MeCN for 30 minutes and treated with a 10% DBU solution in DMF (2x 15 minutes) to furnish the crude, immobilized and partially deprotected phosphoribosyl peptide.

Pyrophosphate synthesis
The resin was extensively washed with MeCN and flushed with nitrogen to remove traces of water. The resin was then treated with a solution of adenosine amidite **12** (3 eq., 0.13 M in MeCN) and DCI (6 eq., 0.25 M in MeCN) for 30 minutes. The resin was thoroughly washed with MeCN before a CSO solution (0.5 M in MeCN) was added to the resin and shaken for 30 minutes.

Final deprotection and cleavage
The resin was then treated with a 10% DBU solution in DMF (2x 10 minutes) to remove the cyano-ethyl protecting group. The resin was then treated with aTBAF (1 M) solution in THF (2x 45 minutes) and washed with DMF followed by DCM. Final cleavage/deprotection occurred by treating the resin with a cleavage cocktail (2.5/10/87.5 v/v/v TIS/TFA/DCM) for 4 hours. The crude peptide was precipitated by flushing the cleavage cocktail in an ice-cold 1/1 mixture of Et_2O/pentane. The resin was washed twice with cleavage cocktail. The crudes were stored at -20 °C overnight to induce as much precipitation as possible before the crudes were centrifuged. The liquids were decanted obtaining the solid crude peptide as precipitate.

Ac-Gly-Arg(5-*O*-adenosine-diphosphate-α,β-D-ribosyl)-Leu-Ile-Phe-Ala-Gly-OH (14)

Via ribosyl building block 1α
The general procedures described above were applied to 25 µmol TentaGel® S AC resin preloaded with Gly. General protocol A was performed for the peptide synthesis. The amino acids used were Fmoc-Ala-OH, Fmoc-Phe-OH, Fmoc-Ile-OH, Fmoc-Leu-OH, Fmoc-Orn(Alloc)-OH and Fmoc-Gly-OH. The crude peptide was purified by RP-HPLC in NH_4OAc buffer. The pure fractions were concentrated, co-evaporated extensively with a 1:1 mixture of MeCN:Milli-Q water, redissolved in Milli-Q water and lyophilized to obtain the title compound as a white solid (6.09 mg, 4.51 µmol, 9.0%). **^{1}H NMR:** (850 MHz, D_2O) δ 8.54 – 8.43 (m, 2H, H-2 adenine α/β), 8.24 (s, 2H, H-8 adenine α/β), 7.28 – 7.25 (m, 4H, Phe arom. α/β), 7.25 – 7.20 (m, 2H, Phe arom. α/β), 7.20 – 7.15 (m, 4H, Phe arom. α/β), 6.09 (d, J = 5.8 Hz, 2H, H-1' adenosine), 5.31 (d, J = 4.3 Hz, 1H, H-1' ribosyl α or β), 5.11 (d, J = 5.8 Hz, 1H, H-1' ribosyl α or β). **^{31}P NMR:** (202 MHz, D_2O) δ -10.2, -10.3, -10.3, -10.4, -10.7, -10.7, -10.8, -10.8. **LC-MS:** (10 -> 90% B in A): Rt = 4.23. **HRMS:** $[C_{51}H_{79}N_{15}O_{22}P_2 + H]^+$ found: 1316.5073, calculated: 1316.5072

Ac-Gly-Arg(5-*O*-adenosine-diphosphate-α,β-D-ribosyl)-Leu-Ile-Phe-Ala-Gly-OH (15)

Via ribosyl building block 1β

The general procedures described above were applied to 15 µmol TentaGel® S AC resin preloaded with Gly on the automated SYRO synthesizer. General protocol A was performed for the peptide synthesis. Fmoc-Ala-OH, Fmoc-Phe-OH, Fmoc-Ile-OH, Fmoc-Leu-OH, Fmoc-Orn(Alloc)-OH and Fmoc-Gly-OH. TBDPS was deprotected using 1ml of 8.8M HF/Pyr solution in 2 cycles of 30 minutes each. For pyrophosphorylation, a in literature known Boc-protected adenosine amidite building block[28] was used, using the same conditions as stated in the general procedures. Final deprotection was performed as described in the general procedures, with the exemption of sillyl deprotection using TBAF. The crude peptide was purified by RP-HPLC in NH_4OAc buffer. The pure fractions were concentrated, co-evaporated extensively with a 1:1 mixture of MeCN:Milli-Q water, redissolved in Milli-Q water and lyophilized to obtain the title compound as a white solid (0.79 mg, 0.59 µmol, 3.9%)(**^{1}H NMR:** (600MHz, D_2O) δ 8.48 – 8.46 (m, 1.5H, H-2 adenine α/β), 8.23-8.22 (2, 1H, H-8 adenine α/β), 7.35 – 7.20 (m, 9H, Phe arom. α/β), 6.10 (d, J = 5.9 Hz, 1.4H, H-1' adenosine), 5.31 (d, J = 4.3 Hz, 1H, H-1' ribosyl α or β), 5.12 (d, J = 5.8 Hz, 0.5H, H-1' ribosyl α or β). **^{31}P NMR:** (162 MHz, D_2O) δ -10.2, -10.2, -10.3, -10.4, -10.6, -10.7, -10.7, -10.8. **LC-MS:** (10 -> 90% B in A): Rt = 3.40. **HRMS: HRMS:** $[C_{51}H_{79}N_{15}O_{22}P_2 + H]^+$ found: 1316.5187, calculated: 1316.5072

Ac-Gly-Arg(5-*O*-adenosine-diphosphate-α,β-D-ribosyl)-Phe-Gly-Ala-Ile-Leu-OH (16)

Via ribosyl building block 1α

The general procedures described above were applied to 25 µmol TentaGel® CTC resin preloaded with Leu on the automated Liberty Blue synthesizer. General protocol B was performed for the peptide synthesis. The amino acids used were Fmoc-Ile-OH, Fmoc-Ala-OH, Fmoc-Gly-OH, Fmoc-Phe-OH, and Fmoc-Orn(Alloc)-OH. TBDPS was deprotected using 1ml of 8.8M HF/Pyr solution in 2 cycles of 30 minutes each. For pyrophosphorylation, a in literature known Boc-protected adenosine amidite building block[28] was used, using the same conditions as stated in the general procedures. Final deprotection was performed as described in the general procedures, with the exemption of sillyl deprotection using TBAF. The crude peptide was purified by RP-HPLC in NH_4OAc buffer. The pure fractions were concentrated, co-evaporated extensively with a 1:1 mixture of MeCN:Milli-Q water, redissolved in Milli-Q water and lyophilized to obtain the title compound as a white solid 7.33 mg, 5.4 µmol, 22%). **^{1}H NMR:** (600MHz, D_2O) δ 8.49 – 8.46 (m, 2H, H-2 adenine α/β), 8.22 (s, 1H, H-8 adenine α/β), 7.43 – 7.02 (m, 10H, Phe arom. α/β), 6.09 (m, 1.5H, H-1' adenosine), 5.35 (d, J = 4.3 Hz, 1H, H-1' ribosyl α or β), 5.14 (d, J = 5.8 Hz, 0.6H, H-1' ribosyl α or β). **^{31}P NMR:** (162 MHz, D_2O) δ -10.1, -10.2, -10.3, -10.3, -10.6, -10.6, -10.7, -10.8. **LC-MS:** (10 -> 90% B in A): Rt = 3.53. **HRMS: HRMS:** $[C_{51}H_{79}N_{15}O_{22}P_2 + H]^+$ found: 1316.5673, calculated: 1316.5072

Ac-Gly-Arg(5-*O*-adenosine-diphosphate-α,β-D-ribosyl)-Thr-Phe-OH (17)

Via ribosyl building block 1α

General procedures described above were applied to 25 µmol Tentagel® S AC resin preloaded with phenylalanine. General protocol A was performed for the peptide synthesis. The amino acids used were Fmoc-Thr(Trt)-OH, Fmoc-Orn(Alloc)-OH and Fmoc-Gly-OH. The crude peptide was purified by RP-HPLC in NH_4OAc buffer. The pure fractions were concentrated, co-evaporated extensively with a 1:1 mixture of MeCN:Milli-Q water, redissolved in MilliQ water and lyophilized to obtain compound **17** as a white solid (2.71 mg, 2.47 µmol, 9.9%). **^{1}H NMR** (500 MHz, D_2O) δ 8.45 – 8.38 (m, 2H, H-2 adenine α/β), 8.20 – 8.10 (m, 2H, H-8 adenine α/β), 7.21 – 7.03 (m, 10H, Phe α/β), 6.02 (d, J = 5.8 Hz, 1H, H-1' adenosine α or β), 5.93 (d, J = 5.4 Hz, 1H, H-1' adenosine α or β), 5.25 (d, J = 4.3 Hz, 1H, H-1' ribosyl α or β), 5.05 (d, J = 5.8 Hz, 1H, H-1' adenosine α or β). **^{31}P NMR** (202 MHz, D_2O) δ -10.2, -10.3, -10.3, -10.4, -10.7, -10.7, -10.8, -10.8. **LC-MS:** (0 -> 20% B in A): Rt = 7.15. **HRMS:** $[C_{38}H_{56}N_{12}O_{20}P_2 + H]^+$ found: 1063.3277, calculated: 1063.3282.

Synthesis of full-length ^{R42}Ub^{ADPr} (18) on wang resin

Synthesis was performed using the above mentioned protocol (for the synthesis of peptides **14**-**17**) with exception of the following conditions:

- Alloc deprotection was performed using ($Pd(PPH_3)_4$ (0.2 eq) and PhSiH (20 eq).
- Equivalents were varied in the crucial steps of synthesis (ribosylation (20 eq), phosphitylation (11.2 eq) and ADPr formation (11.2 eq)).
- Instead of CSO a *t*BuOOH solution (0.55 M in MeCN) was used for oxidation. A *t*BuOOH solution of 5.5M in nonane was diluted ten times in MeCN to obtain the solution.
- TFA/TIS/H_2O/Phenol (90.5/2/5/2.5) was used for final resin cleavage/deprotection of ubiquitin.

Solid Phase Peptide Synthesis, biotin-PEG$_2$ coupling

SPPS was performed according to literature procedure[42] on a Syro II MultiSyntech Automated Peptide synthesizer using standard 9-fluorenylmethoxycarbonyl (Fmoc) based solid phase peptide chemistry at 20 µmol scale, using fourfold excess of amino acids relative to pre-loaded preloaded Fmoc-Gly wang resin (0.2 mmol/g, Rapp Polymere GmbH). On position-42 in the peptide sequence arginine was replaced by Fmoc-Orn(Alloc)-OH. After SPPS, 5 µmol Ub_{1-76} (R42 → Alloc ornithine) on resin was treated with PyBOP (3.1 mg, 30 µmol, 5 eq) and Bt-PEG$_2$-COOH (16.1 mg, 30 µmol, 5 eq) in DMF (2 ml). After 5 min of shaking, DIPEA (16 µL, 90 µmol, 15 eq) was added. The reaction mixture was shaken overnight, after which a test cleavage confirmed full conversion of the conjugation. The resin was then washed with DMF and DCM before resuspension in DCM.

*ADPr synthesis (**R42**)*

Deprotection conditions (desilylations, Fm and cyanoethyl deprotections) were performed identical to the synthesis of peptides **14**-**17** described above, however the amounts of equivalents used in the crucial steps of the synthesis were varied (ribosylation, phosphitylation and ADPr formation) as well as the oxidations and final resin release/deprotection. The synthesis was performed on 2.5 µmol wang resin containing Ub_{1-76}

(R42 → Alloc ornithine. Alloc deprotection was performed by treating the resin with a solution of (Pd(PPh_3)$_4$ (1.4 mg, 1.2 µmol, 0.2 eq) and PhSiH (15 µL, 120 µmol, 20 eq) in anhydrous DCM. This was repeated once more and a test cleavage confirmed complete deprotection of the Alloc-group.

The ribosylation was performed using **1α** (40.7 mg, 50 µmol, 20 eq) and $AgNO_3$ (8.5 mg, 50 µmol, 20 eq). After desilylation, the phosphitylation was performed using $(FmO)_2PN(iPr)_2$ **8** (11.2 eq, 0.13 M in MeCN) and DCI (22.4 eq, 0.25 M in MeCN) and full conversion was confirmed by a test cleavage. Oxidation was performed using a 0.55 M solution of *t*BuOOH in MeCN for 30 minutes. In the final ADPr formation step TBS-protected adenosine amidite **12** (11.2 eq, 0.13 M in MeCN) was used and DCI (22.4 eq, 0.25 M in MeCN). The resin was thoroughly washed with MeCN before a *t*BuOOH solution (0.55 M in MeCN) was added to the resin and shaken for 30 minutes.

Final deprotection, cleavage and purification

After deprotection of the ADPr moiety (cyanoethyl with DBU and TBS with TBAF) identically done as for peptides **14** and **15** the resin was treated with TFA/TIS/H_2O/Phenol (90.5/2/5/2.5) for 1.5 hours before filtrated in an ice-cold solution of Et_2O:pentane (1:1). The precipitate formed was centrifuged (5 min, 3500 rpm) and the supernatant decanted. The pellet was subsequently dried with N_2, taken up in warm DMSO and diluted in warm water before purification by RP-HPLC. Pure fractions were pooled and lyophilized affording $^{R42}Ub^{ADPr}$ **18** (421 µg, 0.044 µmol, 1.75% total yield as a 75.8:24.2 mixture of (Ub^{ADPr}:Ub^{Pr}) as a white powder. **LC-MS**: Rt = 1.47 min. Deconvoluted mass = 9604. **HRMS:** $[C_{416}H_{688}N_{114}O_{139}P_2S + 7H]^{7+}$ found: 1373.3523, calculated: 1373.1071. $[C_{416}H_{688}N_{114}O_{139}P_2S + 8H]^{8+}$ found: 1201.8162, calculated: 1201.5938. $[C_{416}H_{688}N_{114}O_{139}P_2S + 9H]^{9+}$ found: 1068.3911, calculated: 1068.1944. $[C_{416}H_{688}N_{114}O_{139}P_2S + 10H]^{10+}$ found:961.6514, calculated: 961.4748. $[C_{416}H_{688}N_{114}O_{139}P_2S + 11H]^{11+}$ found: 874.3194, calculated: 874.1591.

Synthesis of full-length $^{R54}Ub^{ADPr}$ (19) on wang resin

On position 54 in the peptide sequence arginine was replaced by Fmoc-Orn(Alloc)-OH and the procedure described for R42 was followed.

*ADPr synthesis (***R54***)*

Synthetic procedure was identical to the synthesis described for **$^{R42}Ub^{ADPr}$ 18** with exception of the equivalents used in the following conditions: ribosylation, phosphitylation and ADPr formation. The synthesis was performed on 5 µmol wang resin. The ribosylation was performed using **1α** (61 mg, 75 µmol, 15 eq) and $AgNO_3$(12.7 mg, 75 µmol, 15 eq). Performing the phosphitylation amidite **8** $(FmO)_2PN(iPr)_2$ (15 eq., 0.13 M in MeCN) and DCI (30 eq, 0.25 M in MeCN) were used. In the final ADPr formation step TBS-protected nucleoside amidate **12** (22 eq., 0.13 M in MeCN) and DCI (44 eq., 0.25 M in MeCN) were used. After resin cleavage and precipitation the crude was purified by RP-HPLC. Pure fractions were pooled and lyophilized affording $^{R54}Ub^{ADPr}$ **19** (865 µg, 0.090 µmol, 1.75% total yield as a 85.3:14.7 mixture of (Ub^{ADPr}:Ub^{Pr}) as a white powder. **LC-MS**: Rt = 1.47 min. Deconvoluted mass = 9604. **HRMS:** $[C_{416}H_{688}N_{114}O_{139}P_2S + 7H]^{7+}$ found: 1373.0187, calculated: 1373.1071. $[C_{416}H_{688}N_{114}O_{139}P_2S + 8H]^{8+}$ found: 1201.5184, calculated: 1201.5938. $[C_{416}H_{688}N_{114}O_{139}P_2S + 9H]^{9+}$ found:

1068.1235, calculated: 1068.1944 [$C_{416}H_{688}N_{114}O_{139}P_2S$ + 10H]$^{10+}$ found: 961.4104, calculated: 961.4748. [$C_{416}H_{688}N_{114}O_{139}P_2S$ + 11H]$^{11+}$ found: 874.1077, calculated: 874.1591.

Synthesis of full-length ^{R72}Ub^{ADPr} (20) on wang resin

On position 72 in the peptide sequence arginine was replaced by Fmoc-Orn(Alloc)-OH.

*ADPr synthesis (***R72***)*
Synthetic procedure was identical to the synthesis described for **^{R42}Ub^{ADPr} 18** with exception of the equivalents used in the following conditions: ribosylation, phosphitylation and ADPr formation. The synthesis was performed on 5 µmol wang resin. The ribosylation was performed using **1α** (81.4 mg, 0.10 mmol, 20 eq) and $AgNO_3$(17.0 mg, 0.10 mmol, 20 eq). During the phosphitylation using amidate **8** $(FmO)_2PN(iPr)_2$ (25 eq, 0.13M in MeCN) and DCI (50 eq, 0.25M in MeCN) a product ratio of (55:45) between mono-phosphorylation (M + H)$^{1+}$ = 9632) and di-phosphorylation (M + H)$^{1+}$ = 10069) was observed. The synthesis was proceeded and in the final ADPr formation step using TBS-protected nucleoside amidate **12** (30 eq, 0.13M in MeCN) and DCI (30 eq, 0.25M in MeCN) we observed the mono- and di-ADPribosylated products. After ADPr-protective group deprotection and additional resin cleavage the mono- and di-ADPribosylated products could be separated by HPLC isolating ^{R72}Ub^{ADPr} **20** (650 µg, 0.058 µmol, 1.2% total yield as a 70.3:29.7 mixture of (Ub^{ADPr}:Ub^{Pr}) as a white powder.**LC-MS**: Rt = 1.47 min. Deconvoluted mass = 9605. **HRMS:** [$C_{416}H_{688}N_{114}O_{139}P_2S$ + 7H]$^{7+}$ found: 1373.0209, calculated: 1373.1071. [$C_{416}H_{688}N_{114}O_{139}P_2S$ + 8H]$^{8+}$ found: 1201.5221, calculated: 1201.5938. [$C_{416}H_{688}N_{114}O_{139}P_2S$ + 9H]$^{9+}$ found: 1068.1351, calculated: 1068.1944. [$C_{416}H_{688}N_{114}O_{139}P_2S$ + 10H]$^{10+}$ found: 961.4160, calculated: 961.4748. [$C_{416}H_{688}N_{114}O_{139}P_2S$ + 11H]$^{11+}$ found: 874.1078, calculated: 874.1591.

Synthesis of full-length ^{R74}Ub^{ADPr} (21) on wang resin

On position 74 in the peptide sequence arginine was replaced by Fmoc-Orn(Alloc)-OH.

*ADPr synthesis (***R74***)*
Synthetic procedure was identical to the synthesis described for **^{R42}Ub^{ADPr} 18** with exception of the equivalents used in the following conditions: ribosylation, phosphitylation and ADPr formation. The synthesis was performed on 5 µmol wang resin. The ribosylation was performed using **1α** (61 mg, 75 µmol, 15 eq) and $AgNO_3$(12.7 mg, 75 µmol, 15 eq). In the phosphitylation reaction amidate **8** $(FmO)_2PN(iPr)_2$ (15 eq, 0.13M in MeCN) and DCI (30 eq, 0.25M in MeCN) were used. In the final ADPr formation step TBS-protected nucleoside amidate **12** (30 eq, 0.13M in MeCN) and DCI (30 eq, 0.25M in MeCN) were used. After resin cleavage and precipitation, the crude was purified by RP-HPLC. Pure fractions were pooled and lyophilized affording ^{R74}Ub^{ADPr} **21** (820 µg, 0.085 µmol, 1.7% total yield as a 75.7:24.3 mixture of (Ub^{ADPr}:Ub^{Pr}) as a white powder. **LC-MS**: Rt = 1.47 min. Deconvoluted mass = 9604. **HRMS:** [$C_{416}H_{688}N_{114}O_{139}P_2S$ + 7H]$^{7+}$ found: 1372.9884, calculated: 1373.1071. [$C_{416}H_{688}N_{114}O_{139}P_2S$ + 8H]$^{8+}$ found: 1201.4900, calculated: 1201.5938. [$C_{416}H_{688}N_{114}O_{139}P_2S$ + 9H]$^{9+}$ found: 1068.1035, calculated: 1068.1944. [$C_{416}H_{688}N_{114}O_{139}P_2S$ + 10H]$^{10+}$ found:

961.4286, calculated: 961.4748.[$C_{416}H_{688}N_{114}O_{139}P_2S$ + 11H]$^{11+}$ found: 874.0895, calculated: 874.1591.

Synthesis of full-length ^{R42}Ub^{ADPr} (22) on wang resin via β-isothiourea 1β

ADPr synthesis ((**R42**) via β-isothiourea **1β**

Synthetic procedure was identical to the synthesis described for **^{R42}Ub^{ADPr} 18** with exception of the equivalents used in the following conditions: ribosylation, phosphitylation and ADPr formation. The synthesis was performed on 2 µmol wang resin. The ribosylation was performed using **1β** ribosyl isothiourea β-anomer (21.2 mg, 26 µmol, 13 eq) and $AgNO_3$(4.42 mg, 26 µmol, 13 eq). Performing the phosphitylation amidite **8** $(FmO)_2PN(i\text{Pr})_2$ (12 eq., 0.13 M in MeCN) and DCI (24 eq, 0.25 M in MeCN) were used. In the final ADPr formation step TBS-protected nucleoside amidate **12** (22 eq., 0.13 M in MeCN) and DCI (44 eq., 0.25 M in MeCN) were used. After resin cleavage and precipitation the crude was purified by RP-HPLC. Pure fractions were pooled and lyophilized affording ^{R42}Ub^{ADPr} **22** (151 µg, 0.0157 µmol, 0.79% total yield as a 55.3:44.7 mixture of Ub^{ADPr}:Ub^{Pr})) as a white powder. LC-MS: Rt = 1.47 min. Deconvoluted mass = 9604. HRMS: [$C_{416}H_{688}N_{114}O_{139}P_2S$ + 7H]$^{7+}$ found: 1373.0187, calculated: 1373.1071. [$C_{416}H_{688}N_{114}O_{139}P_2S$ + 8H]$^{8+}$ found: 1201.5184, calculated: 1201.5938. [$C_{416}H_{688}N_{114}O_{139}P_2S$ + 9H]$^{9+}$ found: 1068.1235, calculated: 1068.1944 [$C_{416}H_{688}N_{114}O_{139}P_2S$ + 10H]$^{10+}$ found: 961.4104, calculated: 961.4748. [$C_{416}H_{688}N_{114}O_{139}P_2S$ + 11H]$^{11+}$ found: 874.1077, calculated: 874.1591.

Synthesis RTN4B fragment: Rho-DPSPVSSTVPAPSPLSAAA **(23)** on rink amide resin

SPPS was performed on a Syro II MultiSyntech Automated Peptide synthesizer using standard 9-fluorenylmethoxycarbonyl (Fmoc) based solid phase peptide chemistry at 10 µmol scale, using fourfold excess of amino acids relative to pre-loaded preloaded Fmoc amino Rink amide resin (Rapp Polymere GmbH). After automated peptide synthesis, diBoc-rhodamine (29 mg, 50 µmol, 5 eq.), PyBOP (29 mg, 50 µmol, 5 eq.) and DIPEA (26 µL, 150 µmol, 15 eq.) were added and the mixture was shaken for 1 hour. A test cleavage confirmed conjugation of rhodamine to the N-terminus. The resin was treated with TFA/TIS/H_2O/Phenol (90.5/2/5/2.5) for 1.5 hours before filtrated in an ice-cold solution of Et_2O:pentane (1:1). The precipitate formed was centrifuged (5min, 3500 rpm) and the supernatant decanted. The pellet was subsequently dried with N_2, taken up in warm DMSO and diluted in warm water before purified by RP-HPLC. Pure fractions were pooled and lyophilized affording **23** (7.13 mg, 3.39 µmol, 33.9%) as an orange powder. **LC-MS:** (26 -> 100% B in A): Rt = 3.66. **HRMS:** [$C_{97}H_{136}N_{22}O_{31}$ + 2H]$^{+}$ found: 1053.9845, calculated: 1053.4849.

Procedures DupA-mediated hydrolysis assays and SdeA-mediated ligation

^{1}H-NMR kinetic DupA-mediated hydrolysis of Heptamer 14.

6 µL heptamer **14** (10 mM stock in H_2O) was added to a NMR tube containing 460 µL buffer (Tris 20 mM, NaCl 150 mM, pH 7.6) and 53 µL D_2O . A reference spectrum was measured on a Bruker 600 mHz in which the H_2O signal was suppressed. The contents of the NMR tube were transferred to an Eppendorf and DupA was added (20 µL of a 889 µM stock solution) to generate final concentrations of DupA (33 µM) and heptamer **14** (111.1 µM). After addition, the mixture was incubated at 37 °C and monitored by HRMS. After 2 hours HRMS indicated conversion and a ^{1}H-NMR was taken suppressing the H_2O signal. The anomeric protons could be visualized and conversion could be monitored as ratio between the integrals of the corresponding intact ADPr moiety protons or dupA-mediated hydrolysis of the pyrophosphate bond phosphoribosyl associated protons. Differences in the hydrolysis-kinetics of the alpha and beta anomer could be visualized in the NMR spectra. An additional ^{1}H NMR spectrum was measured after overnight incubation at 37°C .

DupA mediated hydrolysis of ADPribosylated peptides 14-17 (0-90min)

The peptides **14**-**17** (5 µM) in buffer (20 mM TRIS, 150 mM NaCl, pH 7.6) were incubated with DupA (3 µM) or without (background hydrolysis) at 37°C in a total volume of 50 µL. At the indicated time points 15 µL sample was 4 times diluted before measuring HRMS. The ratio of product versus starting material was determined, corrected for t = 0 min and plotted as increase in pyrophosphate cleavage over time. The means of two individual measurements is depicted with standard deviation and compared to enzym. $^{R42}Ub^{ADPr}$

DupA-mediated hydrolysis of synthetically prepared $^{Rx}Ub^{ADPr}$ (18-21) and heptamer 14, analyzed after overnight incubation

One of the ubiquitin's **18**-**21** (5 µM) or heptamer **14** were incubated with DupA (3 µM) or without (background hydrolysis) at 37°C in a total volume of 30 µL. After overnight incubation 15 µL sample was 4 times diluted before measuring HRMS. The ratio of product versus starting material was determined, corrected for t = 0 min and plotted as increase in pyrophosphate cleavage. The means of two individual measurements is depicted with standard deviation and compared to enzym. $^{R42}Ub^{ADPr}$

DupA-mediated hydrolysis of synthetically prepared $^{Rx}Ub^{ADPr}$ (18-21) (0-90min)

The ubiquitin's **18**-**21** (5 µM) in buffer (20 mM TRIS, 150 mM NaCl, pH 7.6) were incubated with DupA (3 µM) or without (background hydrolysis) at 37°C in a total volume of 50 µL. At the indicated time points 15 µL sample was 4 times diluted before measuring HRMS. The ratio of product versus starting material was determined, corrected for t = 0 min and plotted as increase in pyrophosphate cleavage over time and compared to enzym. $^{R42}Ub^{ADPr}$

SdeA-mediated ligation of $^{Rx}Ub^{ADPr}$(18-21) and RTN4b peptide 23.

The enzymatically prepared $^{R42}Ub^{ADPr}$ or synthetically prepared ubiquitin's **18**-**21** (67 µM) in buffer (20 mM TRIS, 150 mM NaCl, pH 7.5) were incubated with RTN4B fragment **23** (60 µM) and SdeA FL (20 µM), at 37°C in a total volume of 25 µL. The mixture was monitored by HRMS

and after 1 hour the enzymatic and synthetic $^{R42}Ub^{ADPr}$'s indicated conversion to the $^{R42}Ub^{Pr}$-RTN4b complex on mass spectrometry (deconvoluted mass = 11363). The ubiquitin's were analyzed by SDS PAGE adding 10 µL of each sample to 5 µL loading buffer (3X). The samples were run on a NuPAGE™ 12% Bis-Tris gel in MES buffer, 190 mV, for 45 minutes. A fluorescence scan on a Typhoon FLA 9500 (rhodamine channel, 473 nm) was performed to visualize the complex formed and additionally, the proteins were stained with Coomassie staining.

References

28 N. Hanaya, S. K. Daley, J. D. Bagert and T. W. Muir . Synthesis of ADP-Ribosylated Histones Reveals Site-Specific Impacts on Chromatin Structure and Function. *J. Am. Chem. Soc*. 2021, 143, 10847-10852.

36 A. Štimac and J. Kobe, *Carbohydr. Res.*, 1992, 232, 359–365.

42 F. El Oualid, R. Merkx, R. Ekkebus, D. S. Hameed, J. J. Smit, A. de Jong, H. Hilkmann, T. K. Sixma and H. Ovaa, *Angew. Chemie Int. Ed.*, 2010, 49, 10149–10153.

1H, 13C, COSY, HSQC, 31P-NMR-spectra of riboses (1-4) and NMR and HRMS of peptides 1-17

H-NMR compound 3

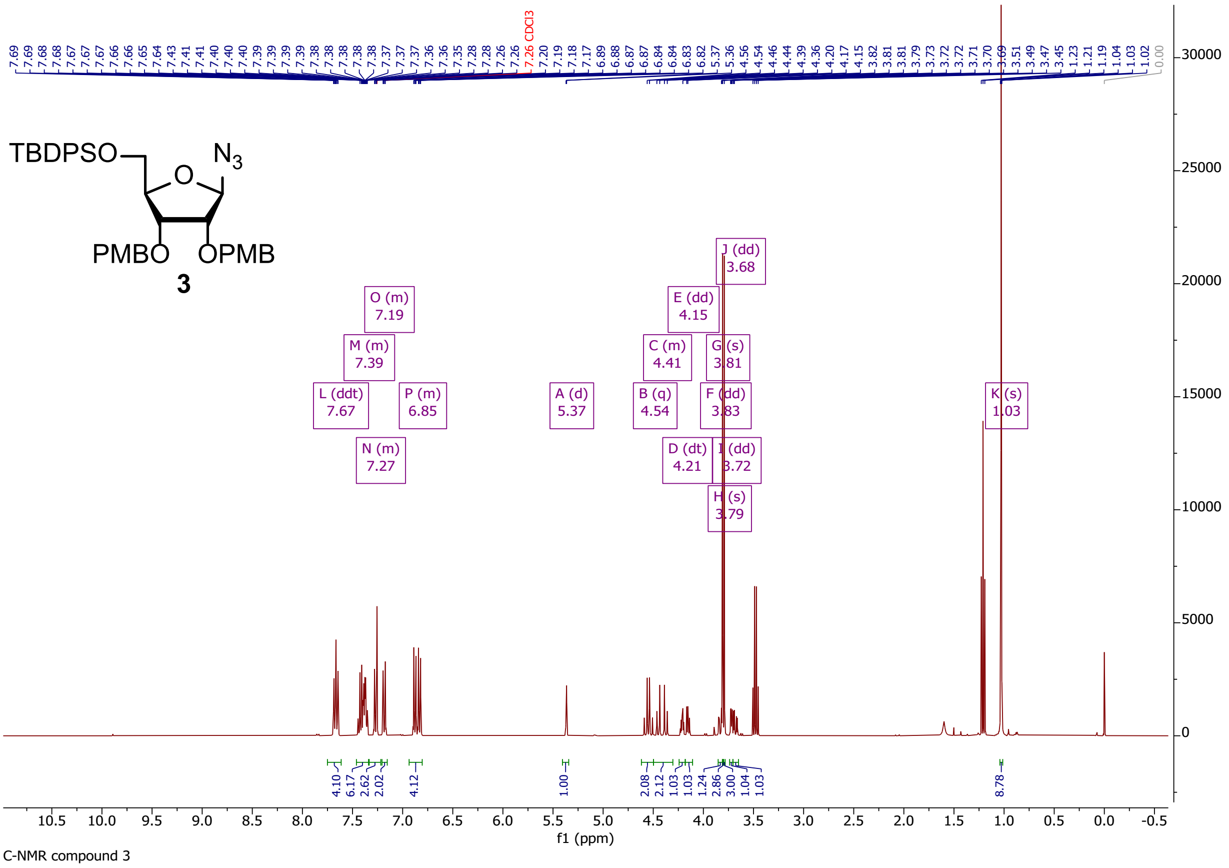

C-NMR compound 3

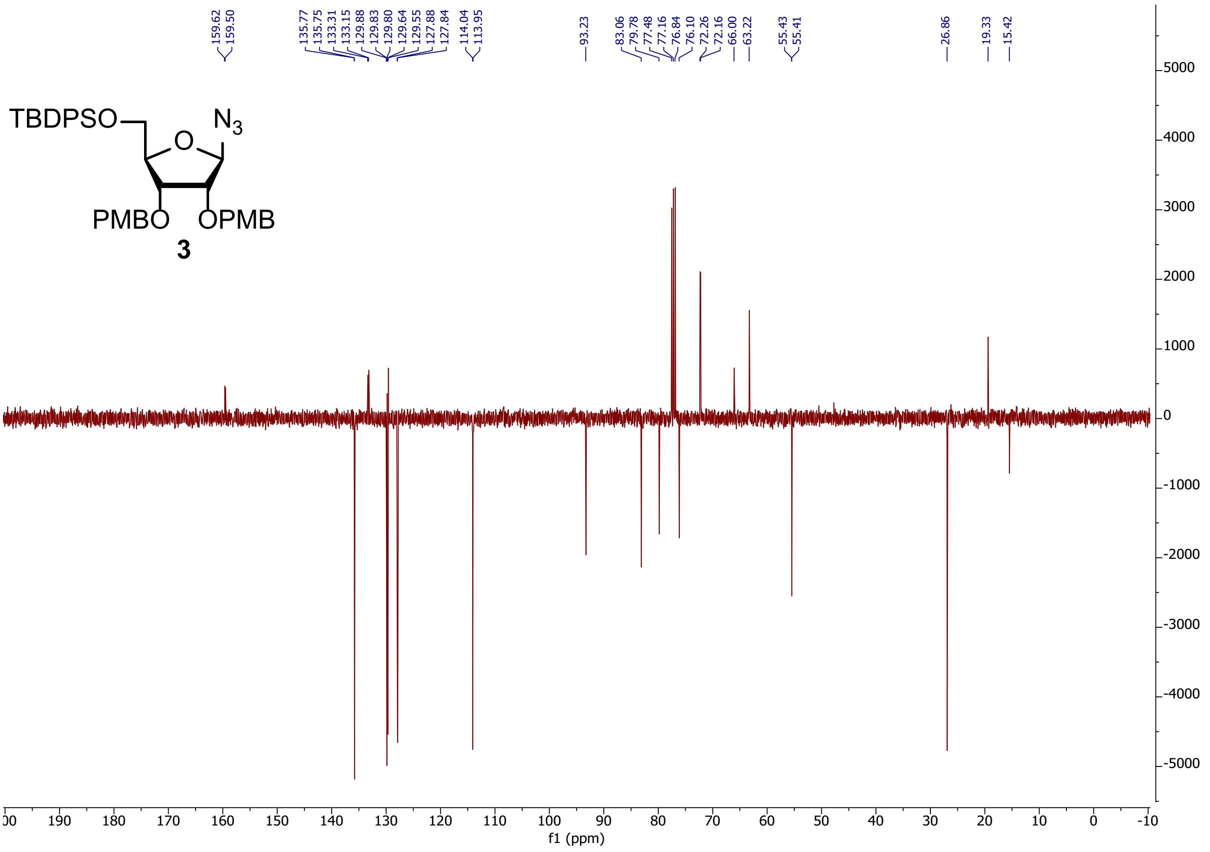

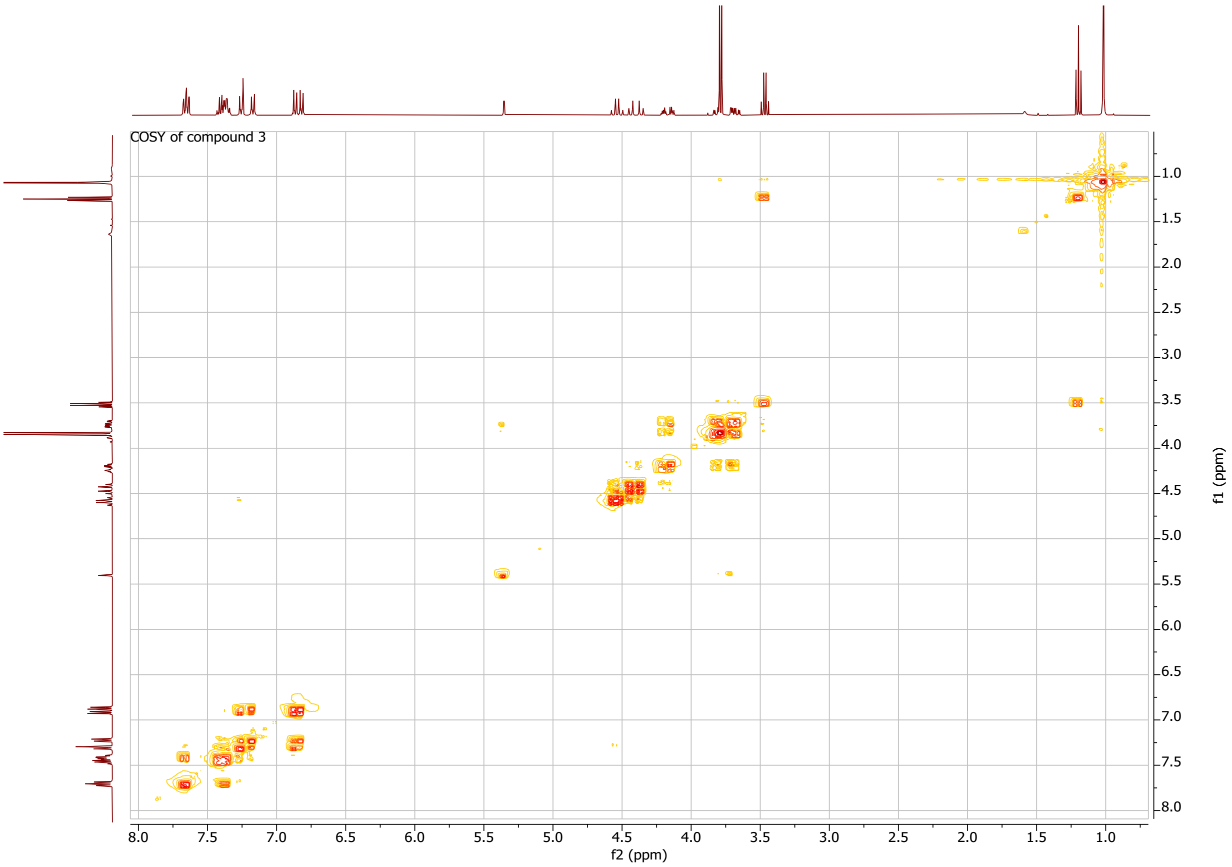
COSY of compound 3
f2 (ppm)
f1 (ppm)

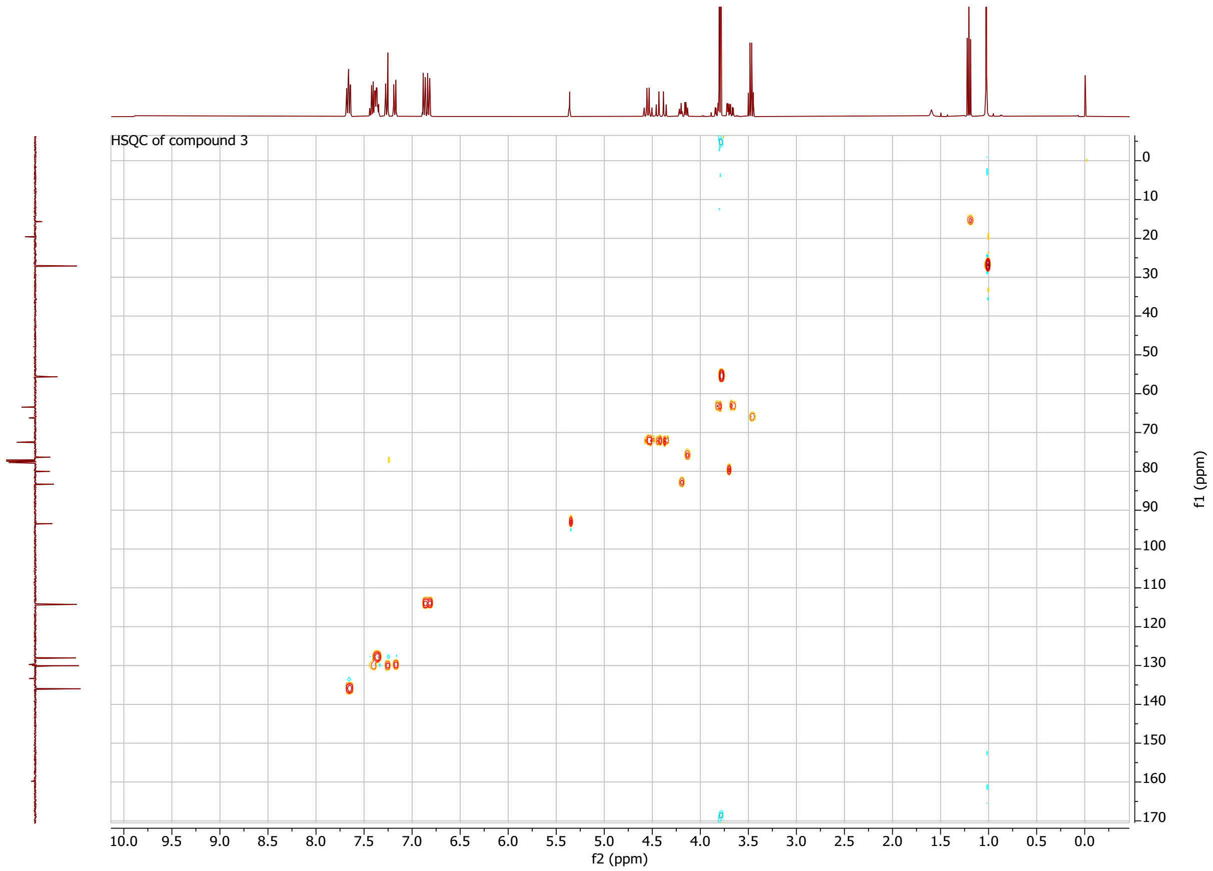
HSQC of compound 3
f2 (ppm)
f1 (ppm)

H-NMR compound 4 (alpha)

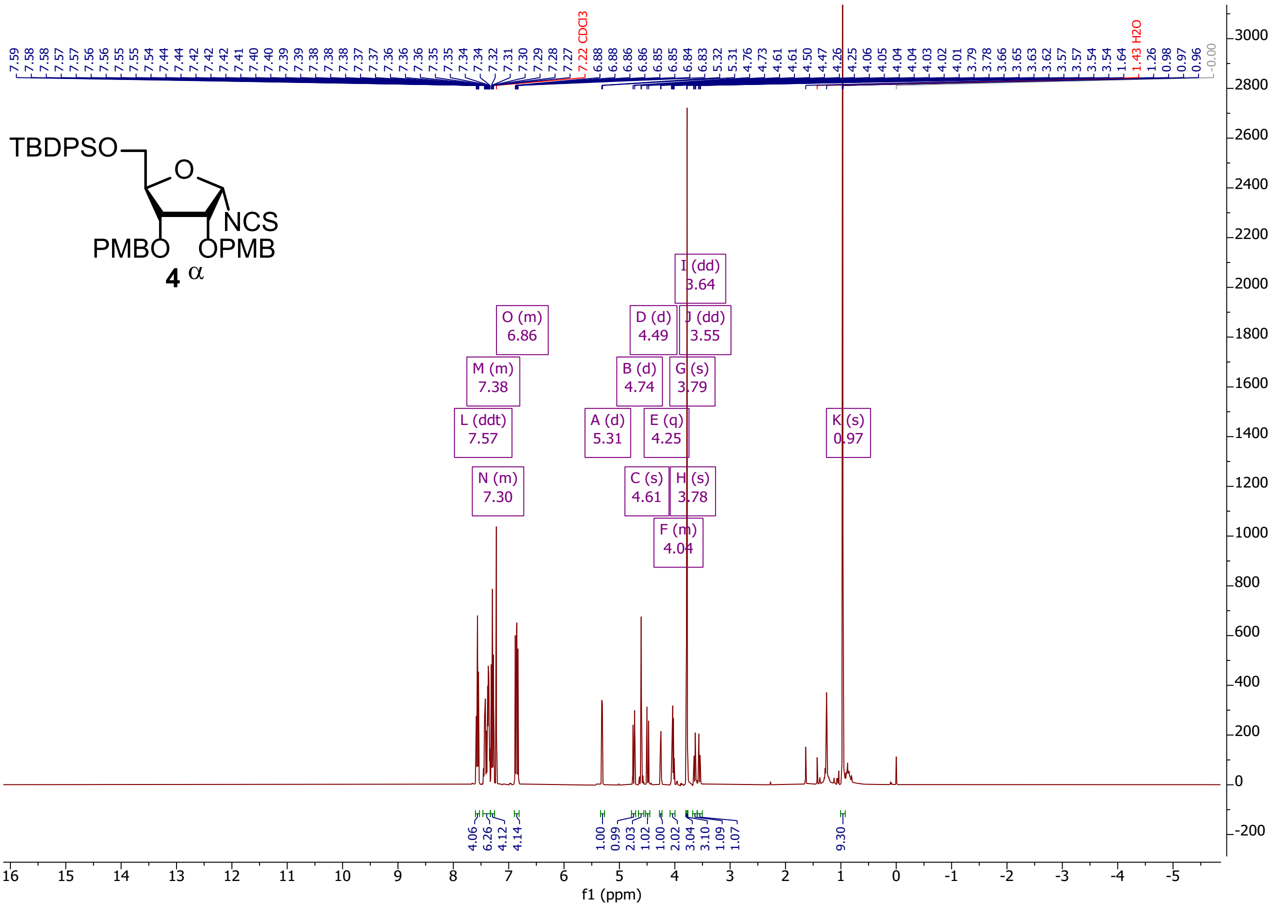

C-NMR compound 4 (alpha)

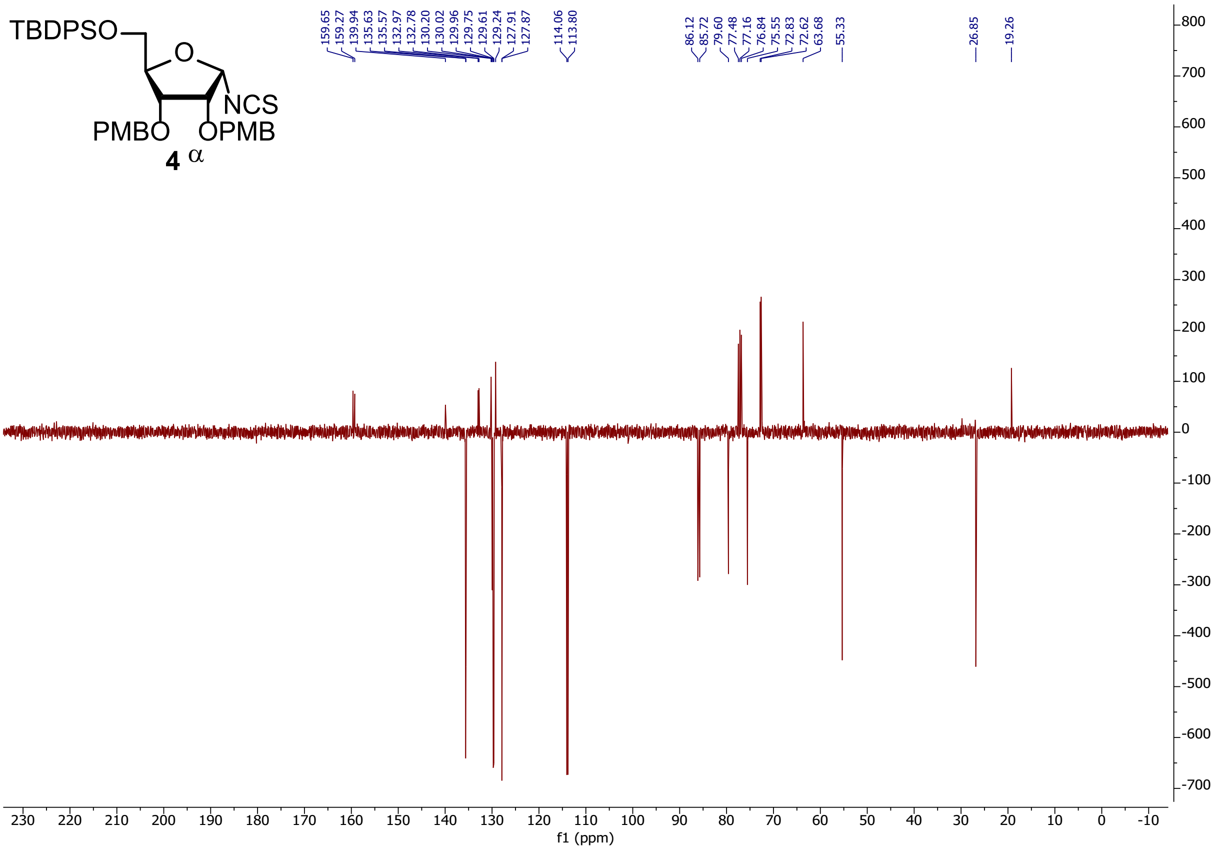

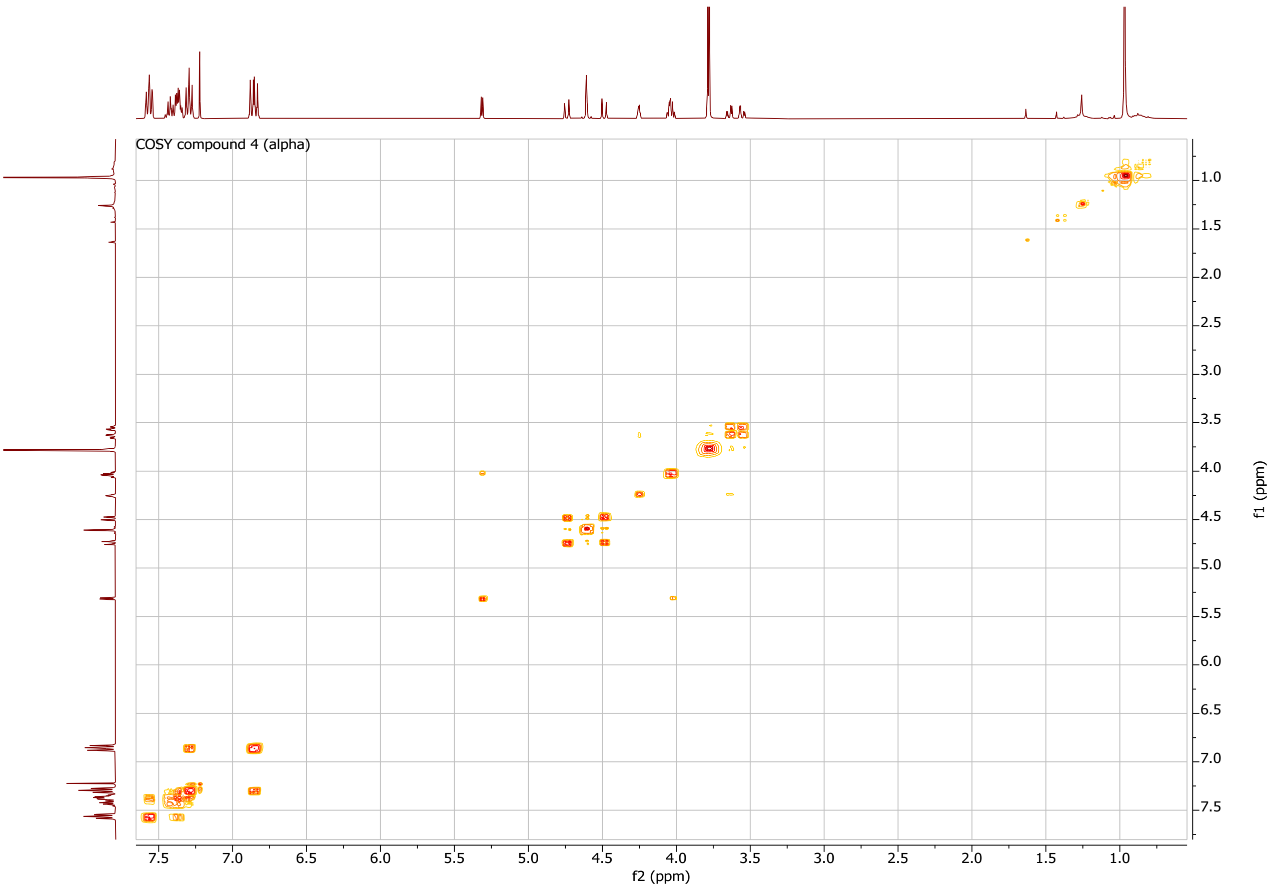
COSY compound 4 (alpha)
f2 (ppm)
f1 (ppm)

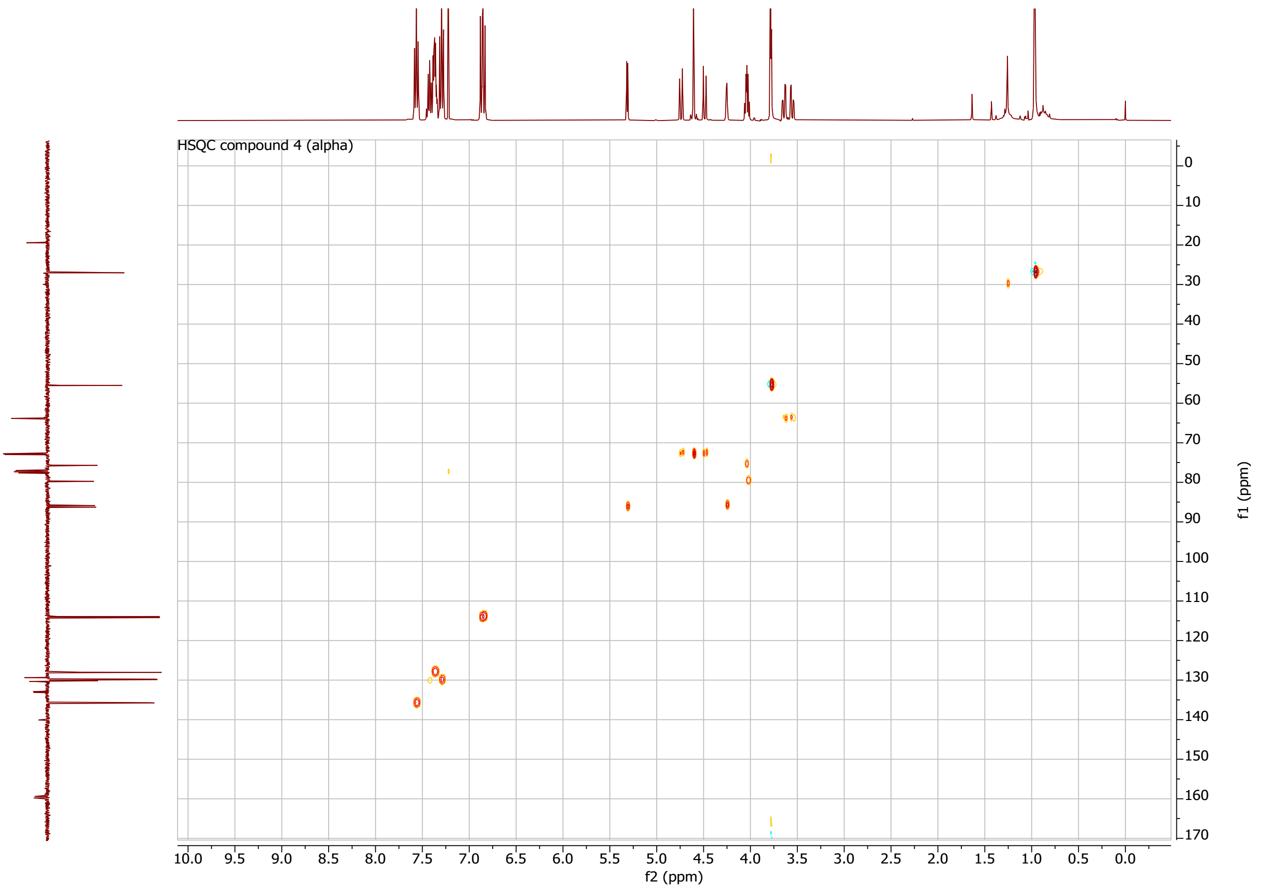
HSQC compound 4 (alpha)
f2 (ppm)
f1 (ppm)

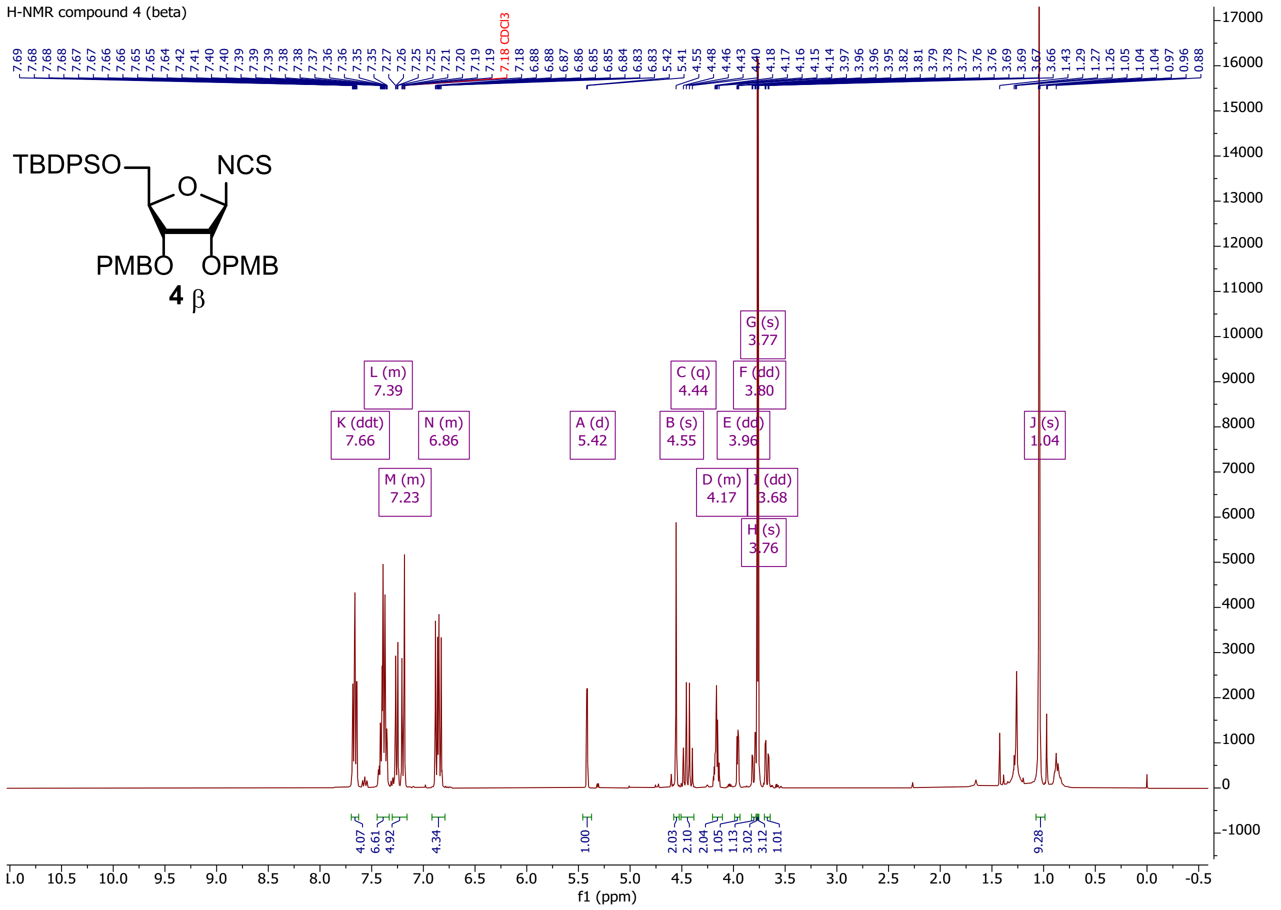
H-NMR compound 4 (beta)
TBDPSO
NCS
O
PMBO
OPMB
4 β
L (m) 7.39
K (ddt) 7.66
N (m) 6.86
M (m) 7.23
A (d) 5.42
C (q) 4.44
B (s) 4.55
G (s) 3.77
F (dd) 3.80
E (dd) 3.96
D (m) 4.17
I (dd) 3.68
H (s) 3.76
J (s) 1.04
4.07 6.61 4.92 4.34 1.00 2.03 2.10 2.04 1.05 1.13 3.02 3.12 1.01 9.28
f1 (ppm)

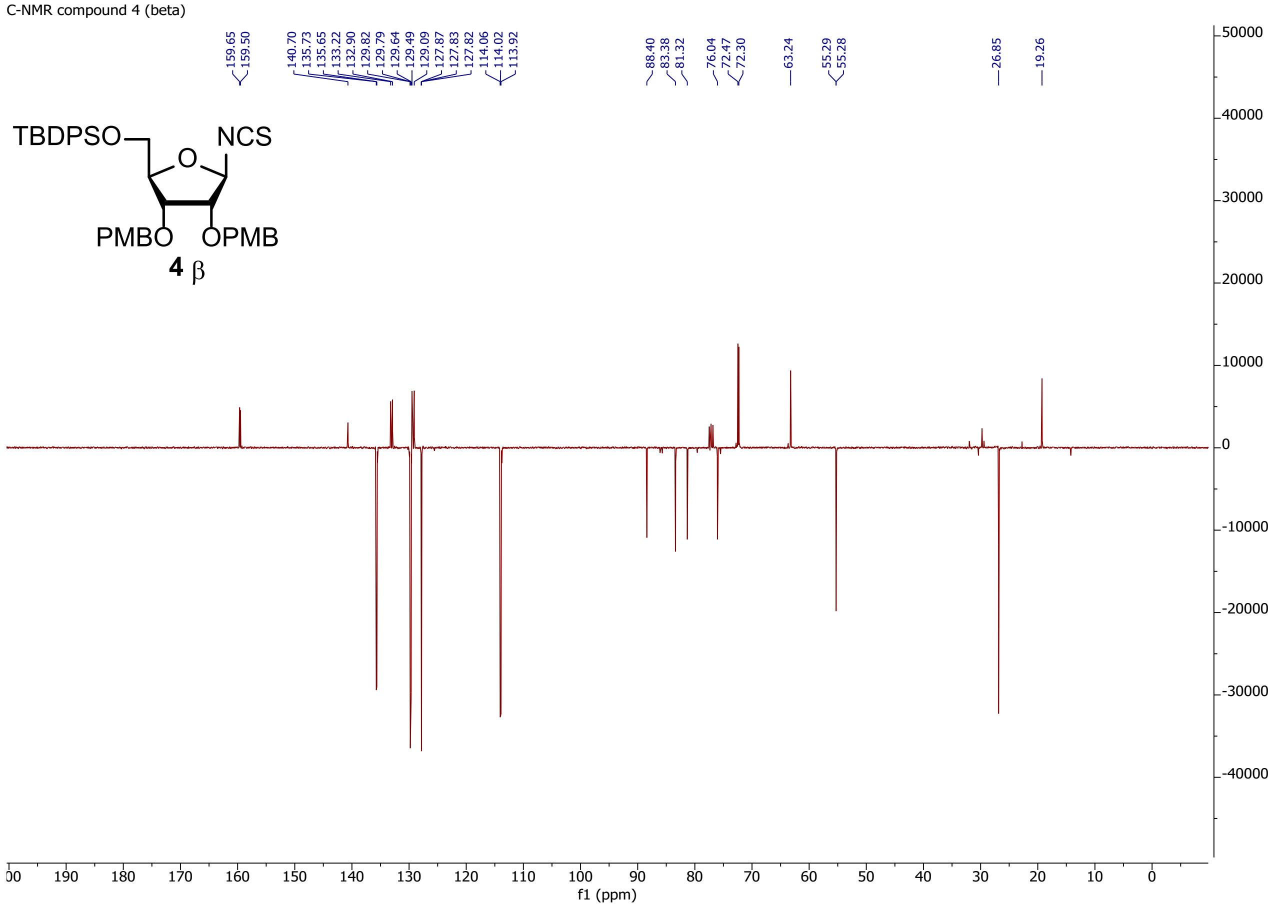
C-NMR compound 4 (beta)
159.65 159.50 140.70 135.73 135.65 133.22 132.90 129.82 129.79 129.64 129.49 129.09 127.87 127.83 127.82 114.06 114.02 113.92 88.40 83.38 81.32 76.04 72.47 72.30 63.24 55.29 55.28 26.85 19.26
TBDPSO
NCS
O
PMBO
OPMB
4 β
f1 (ppm)

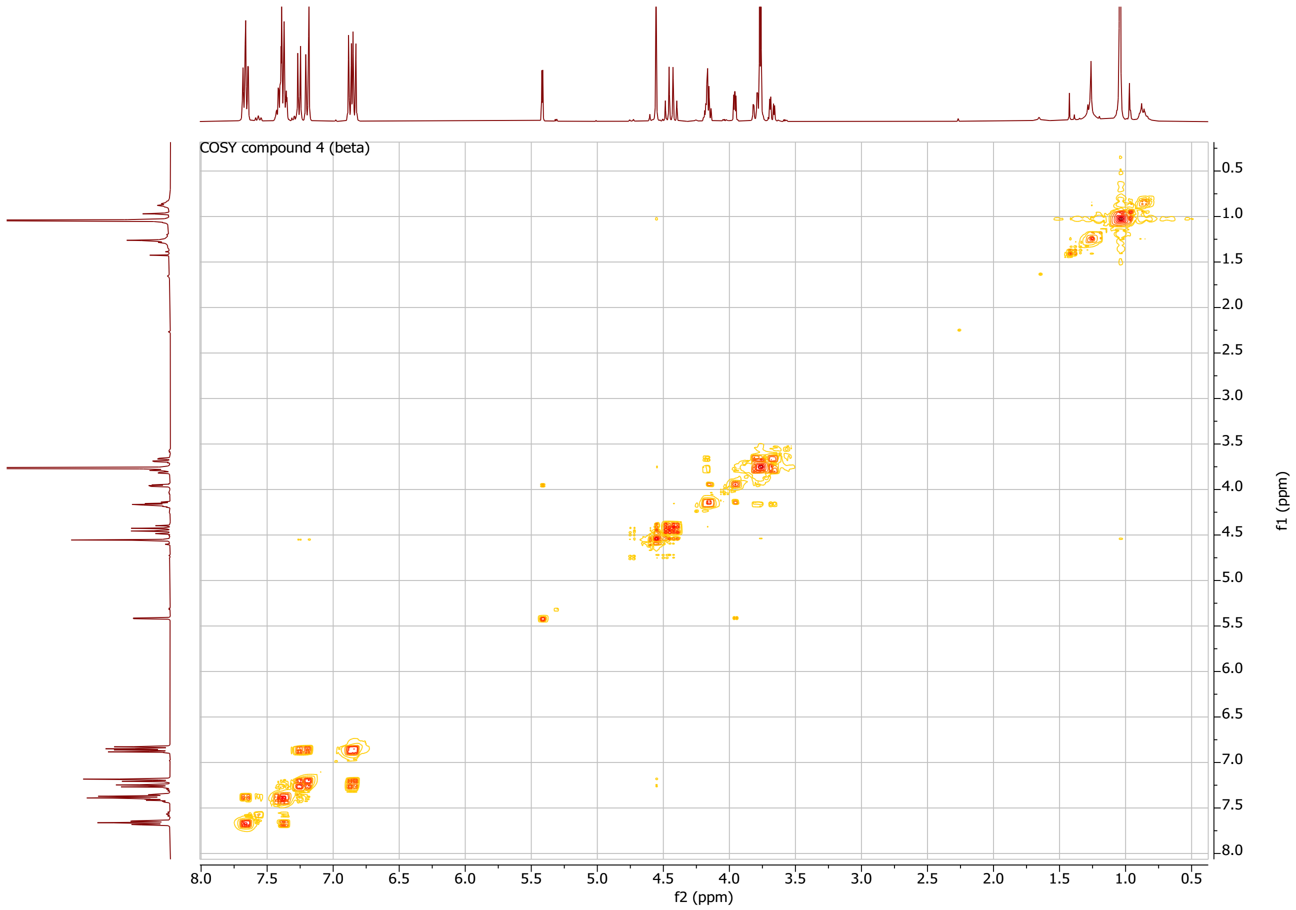
COSY compound 4 (beta)
f2 (ppm)
f1 (ppm)

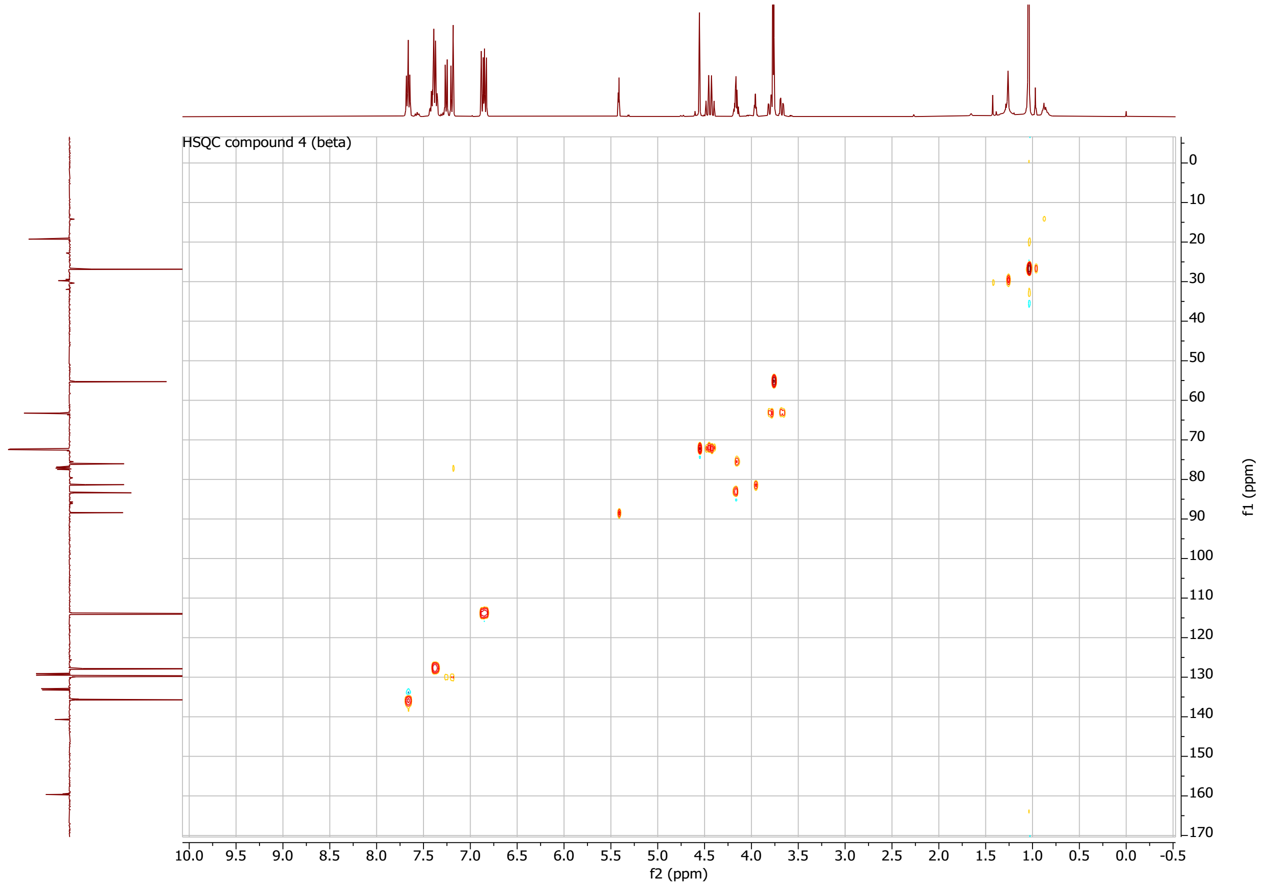
HSQC compound 4 (beta)
f2 (ppm)
f1 (ppm)

H-NMR compound 1

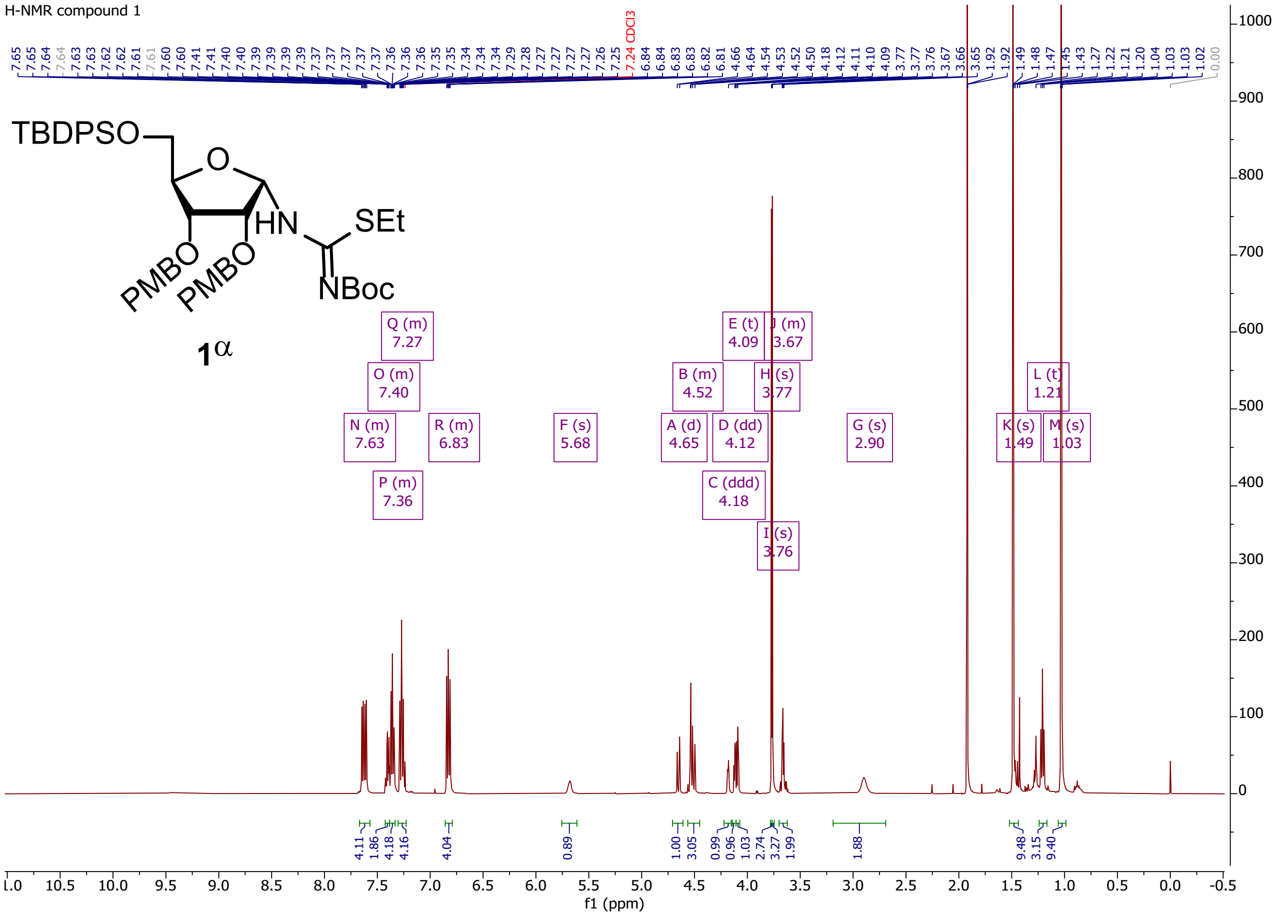

C-NMR compound 1

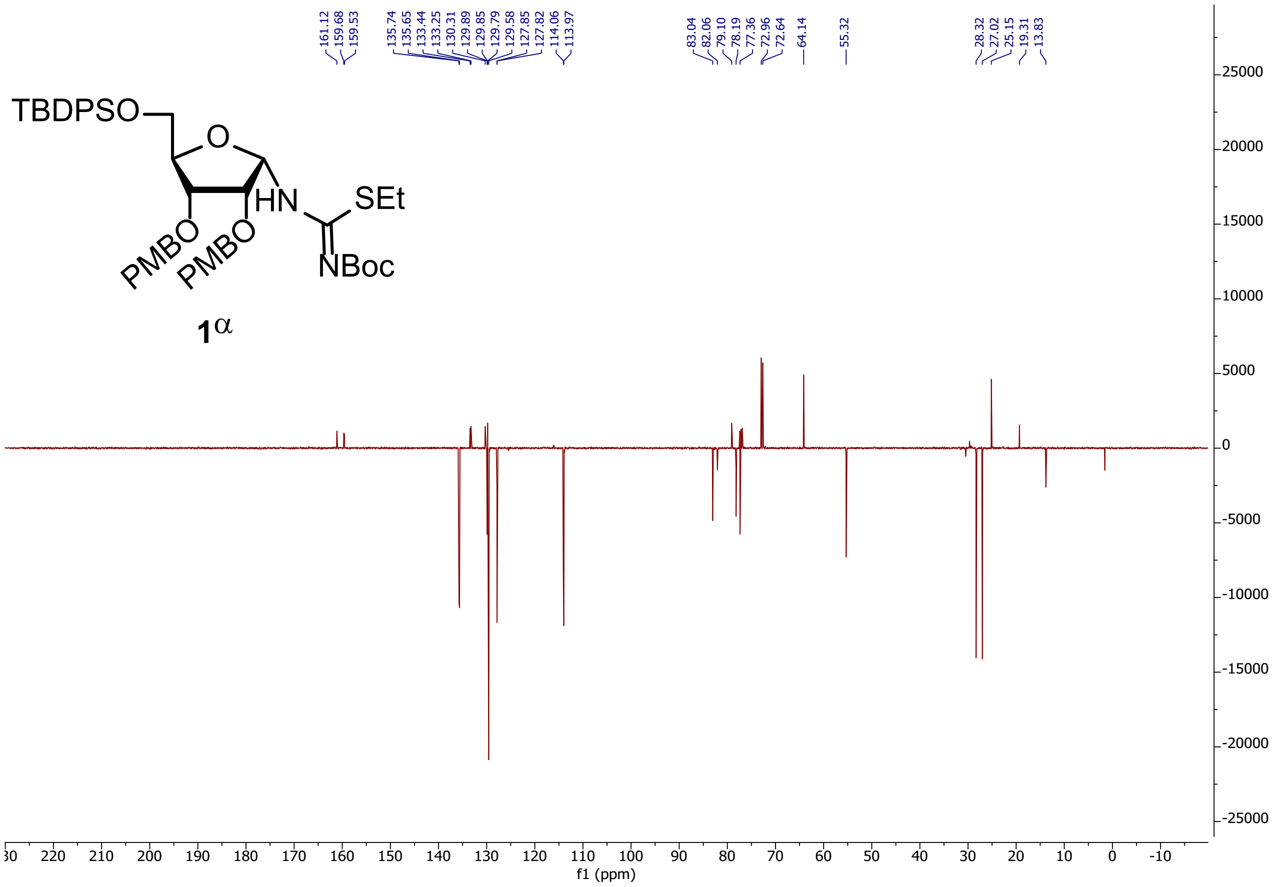

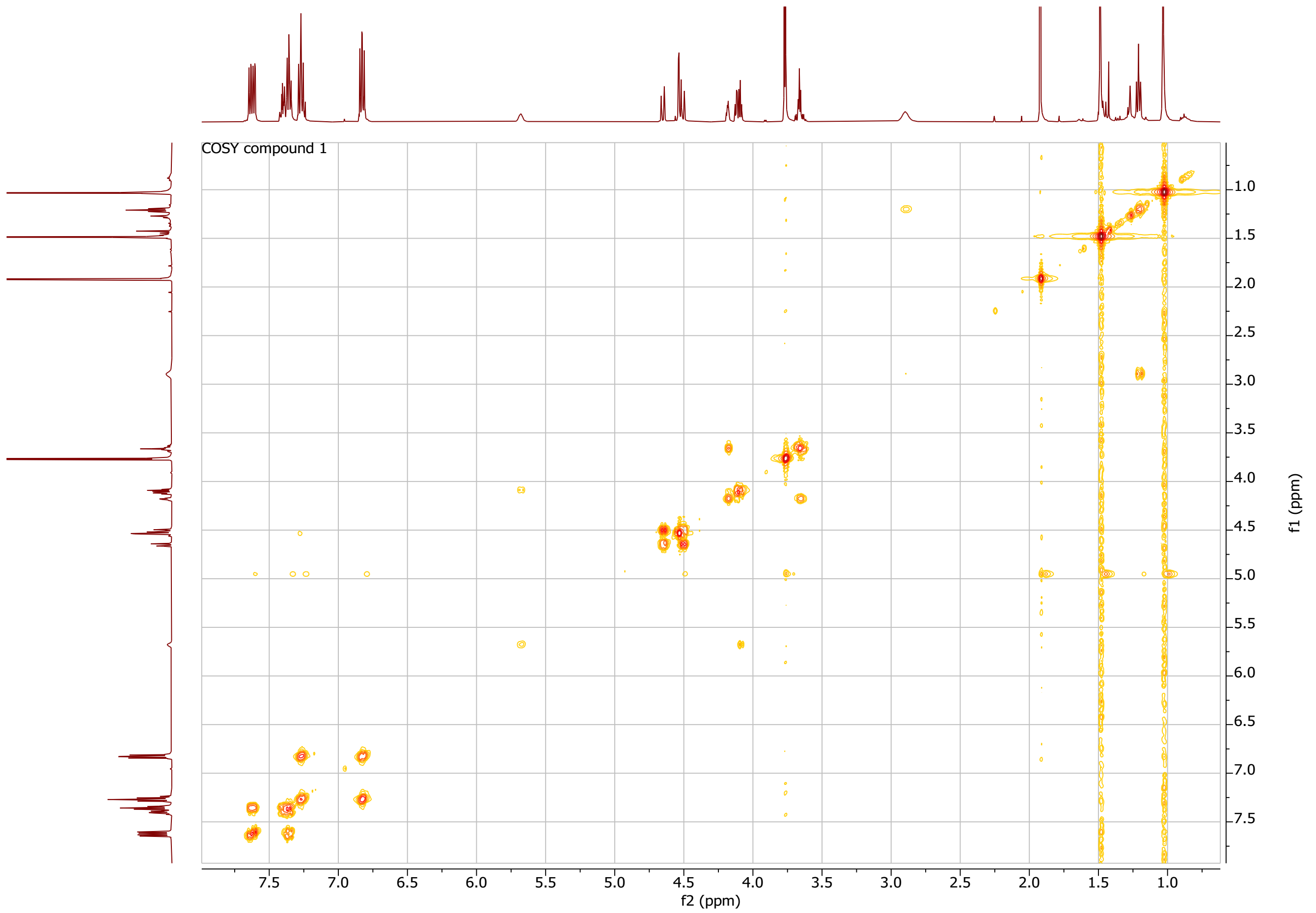
COSY compound 1
f2 (ppm)
f1 (ppm)

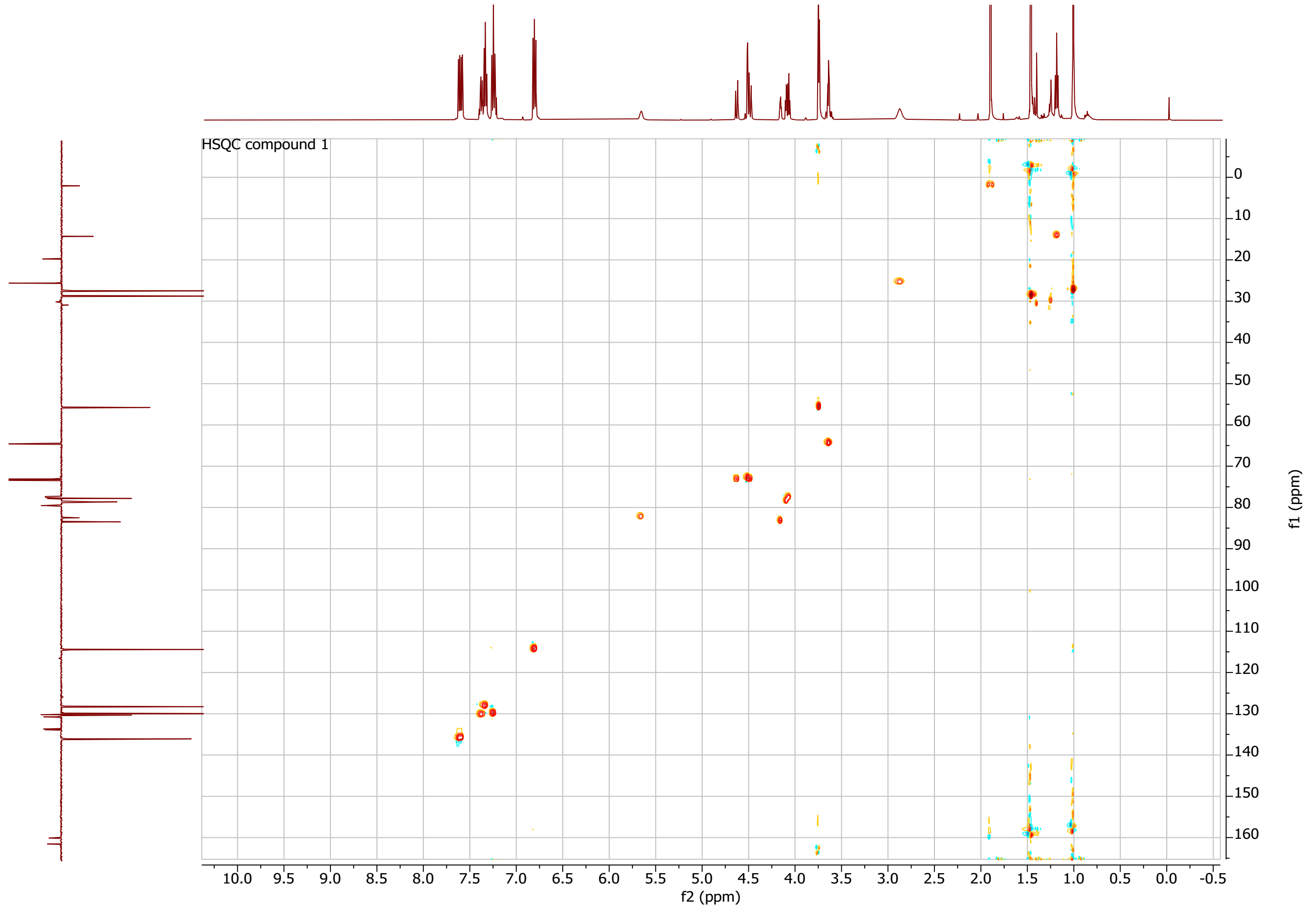
HSQC compound 1
f2 (ppm)
f1 (ppm)

H-NMR compound 1β

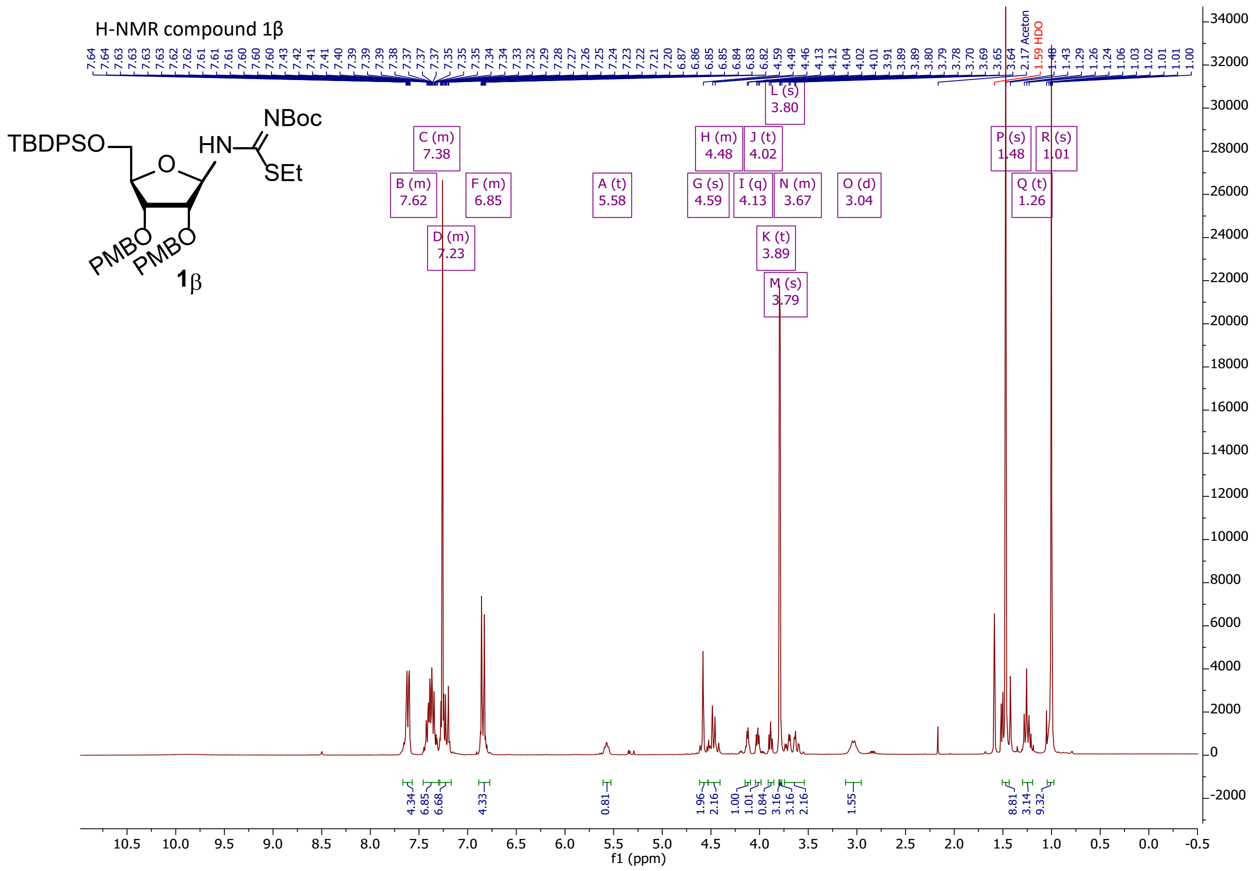

C-NMR compound 1β

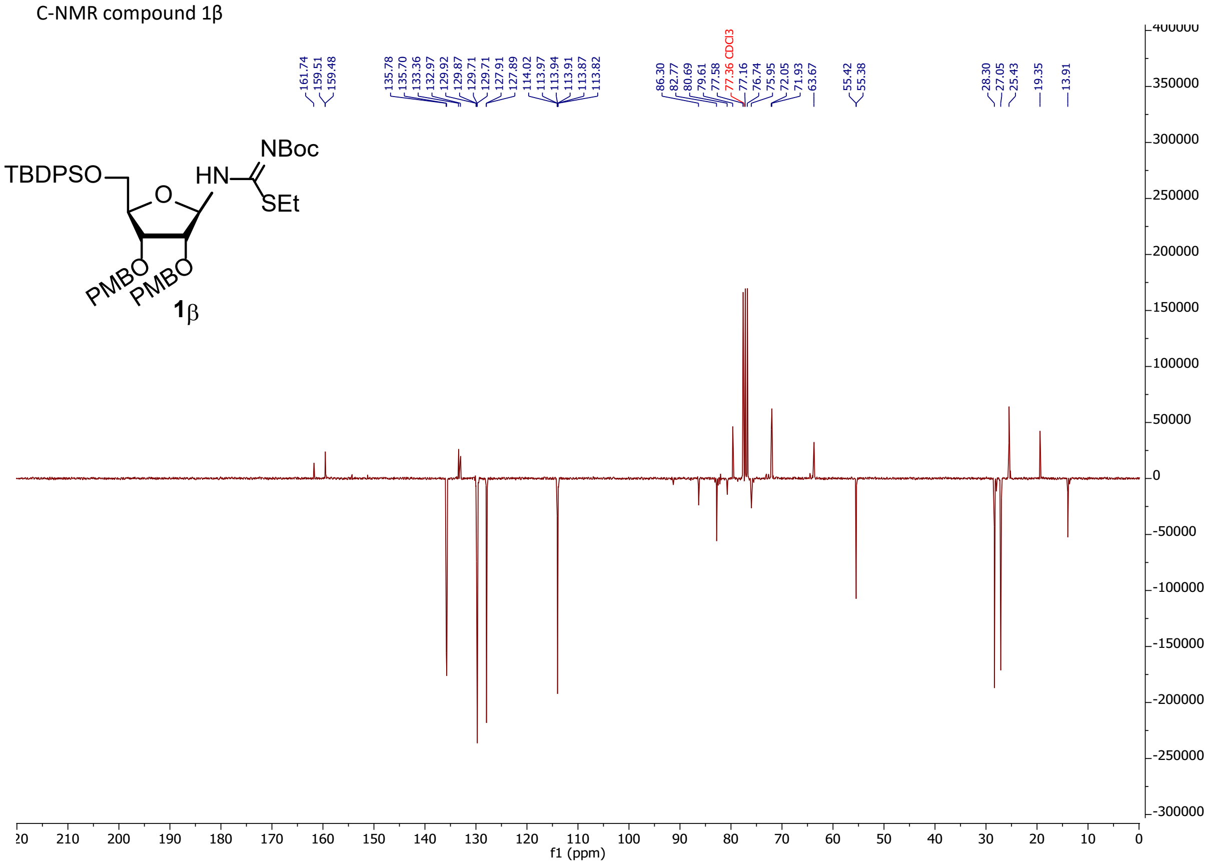

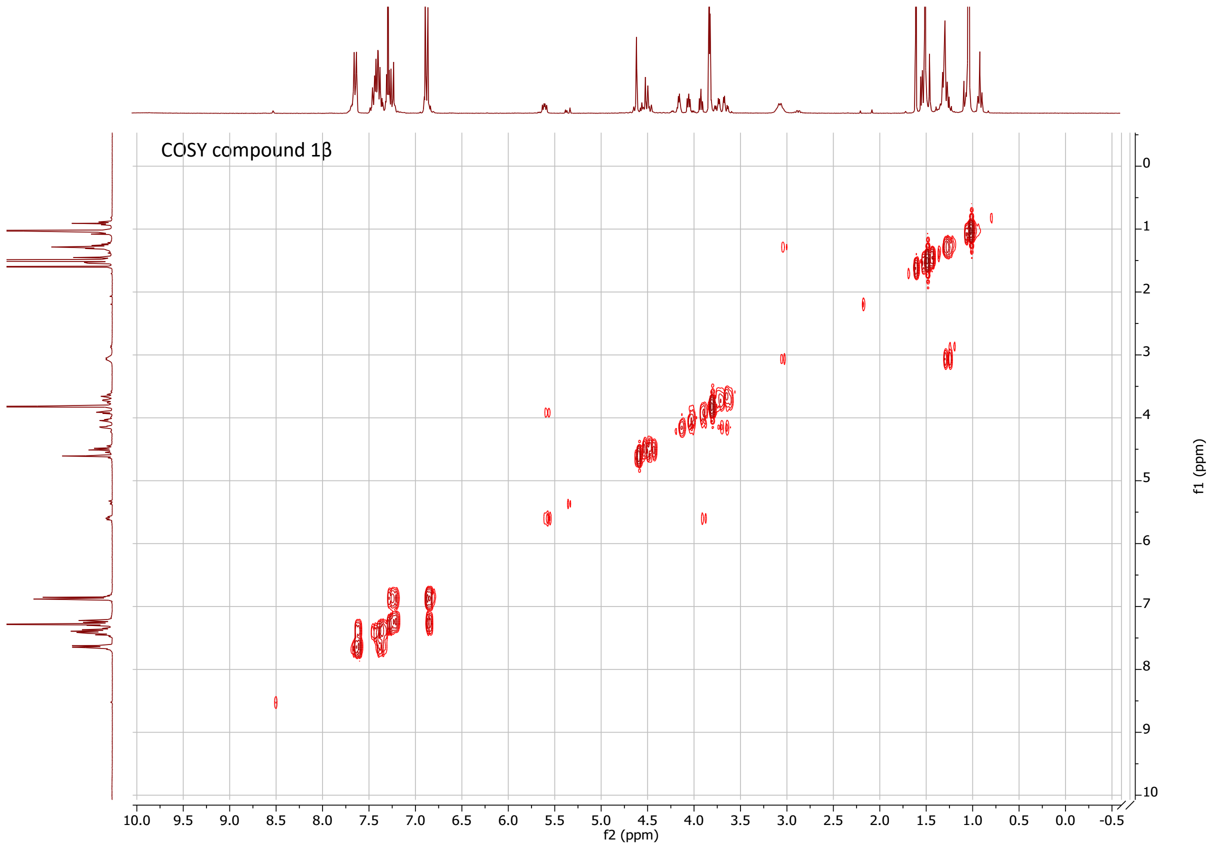
COSY compound 1β
10.0 9.5 9.0 8.5 8.0 7.5 7.0 6.5 6.0 5.5 5.0 4.5 4.0 3.5 3.0 2.5 2.0 1.5 1.0 0.5 0.0 -0.5
f2 (ppm)
0 1 2 3 4 5 6 7 8 9 10
f1 (ppm)

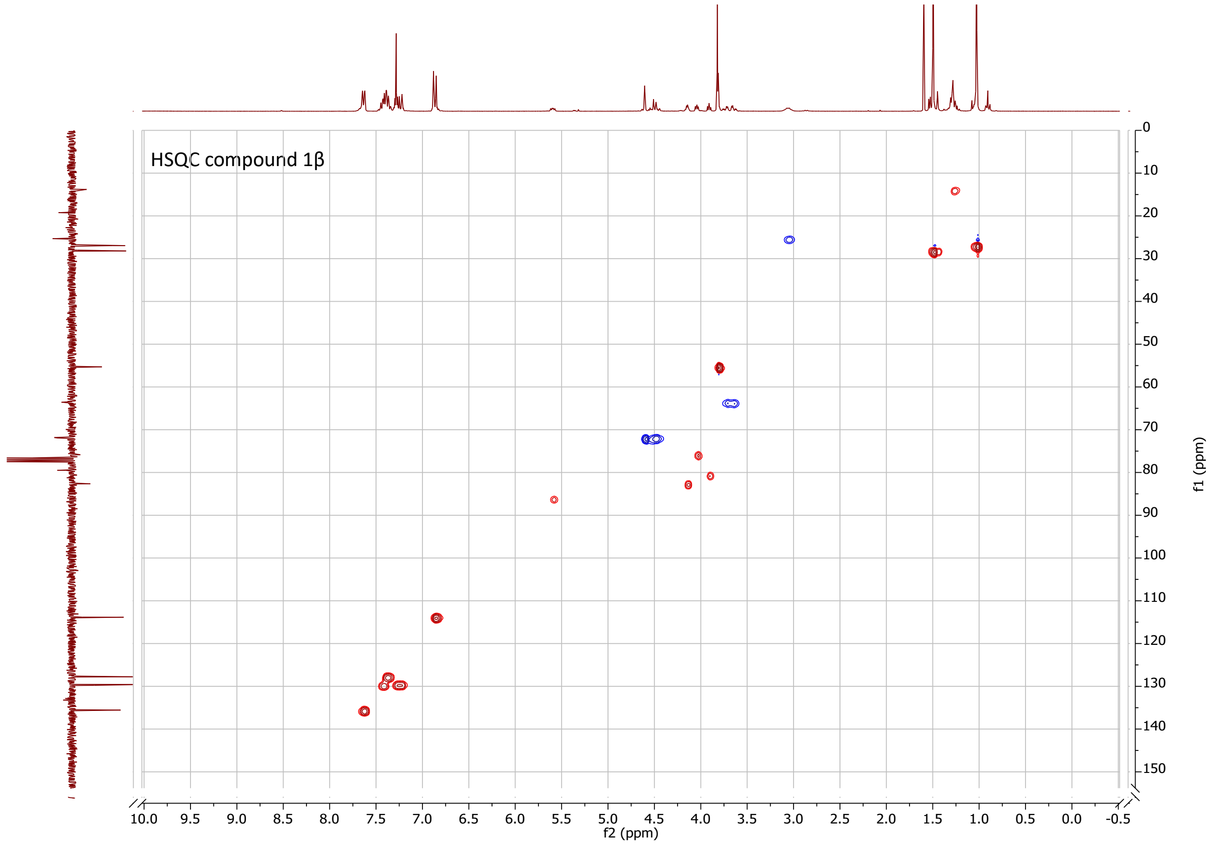
HSQC compound 1β
10.0 9.5 9.0 8.5 8.0 7.5 7.0 6.5 6.0 5.5 5.0 4.5 4.0 3.5 3.0 2.5 2.0 1.5 1.0 0.5 0.0 -0.5
f2 (ppm)
0 10 20 30 40 50 60 70 80 90 100 110 120 130 140 150
f1 (ppm)

HRMS of Arg-ADPribosylated peptide (14) - Via α-isothiourea 1α

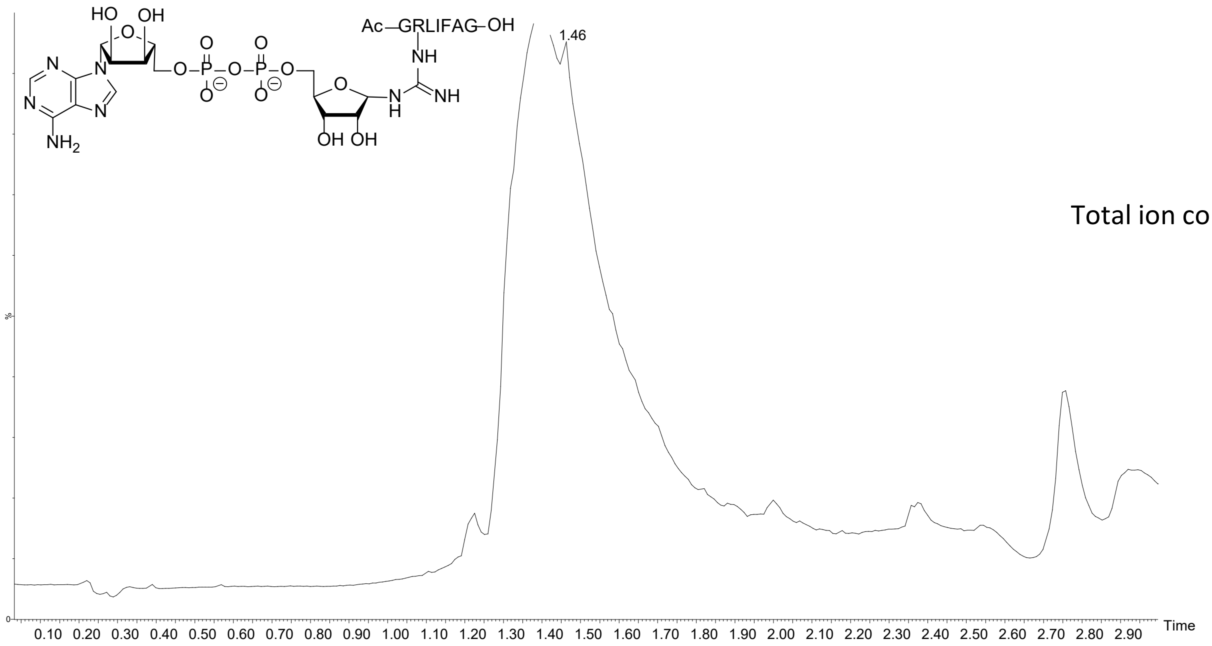

Total ion count

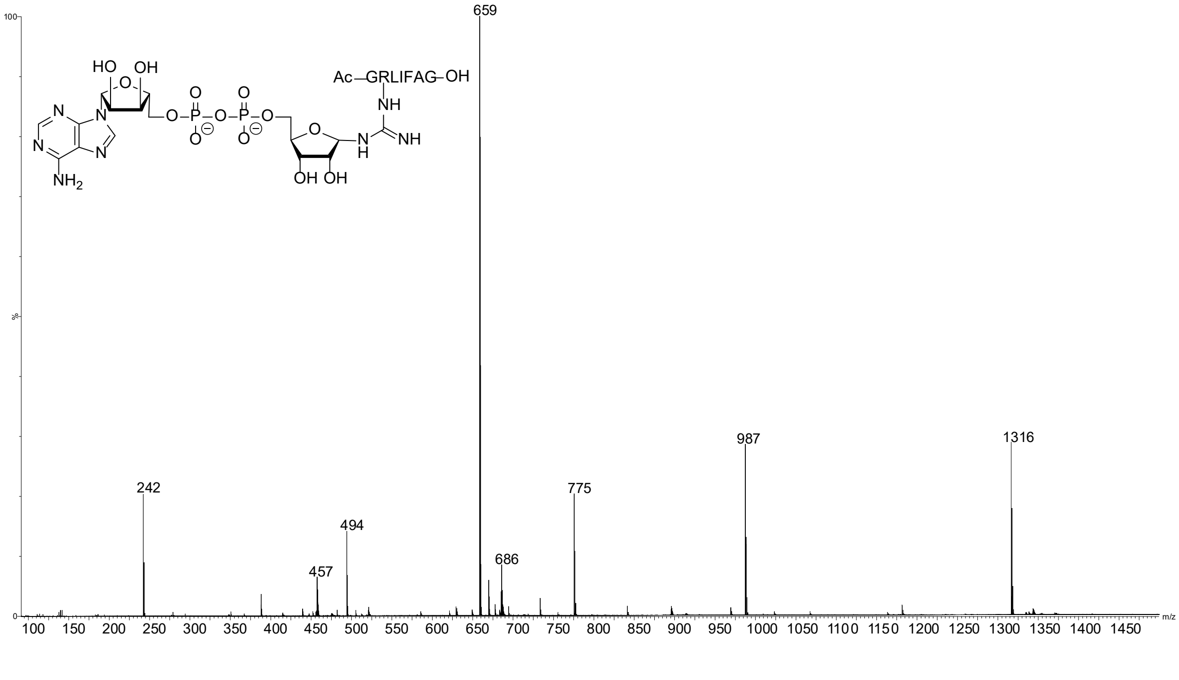

ESI MS

(100-1500 Da)

H-NMR peptide 14

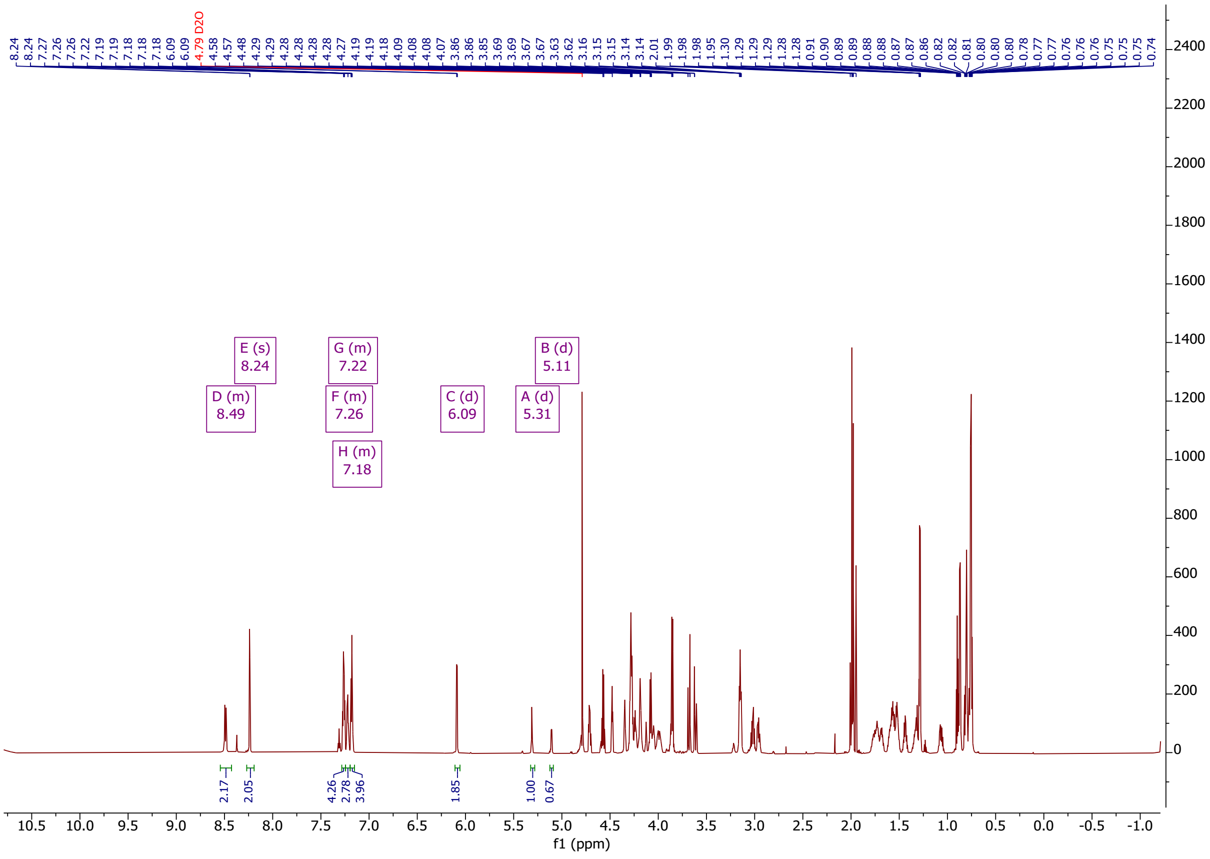

H-NMR peptide 14

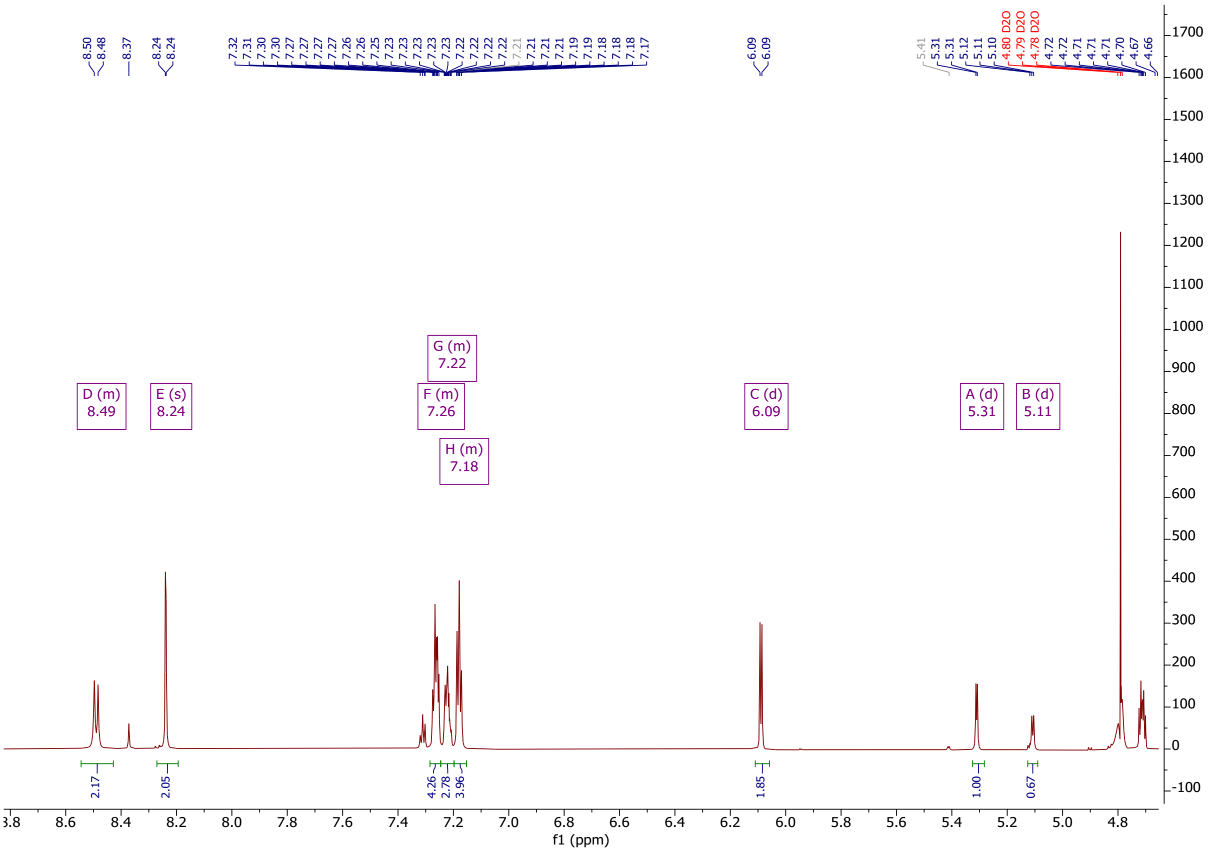

P-NMR peptide 14

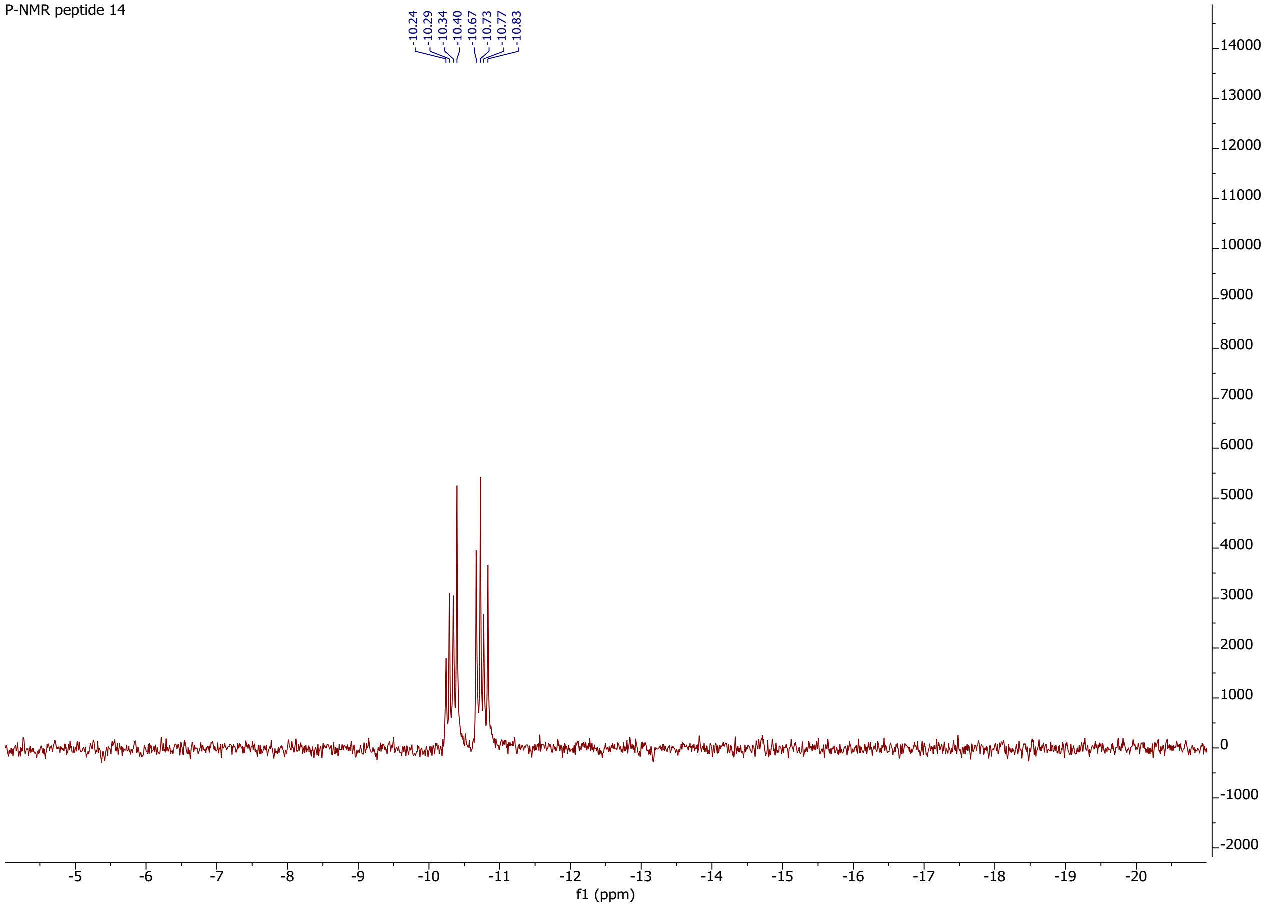

HRMS of Arg-ADPribosylated peptide (15) via β-isothiourea 1β

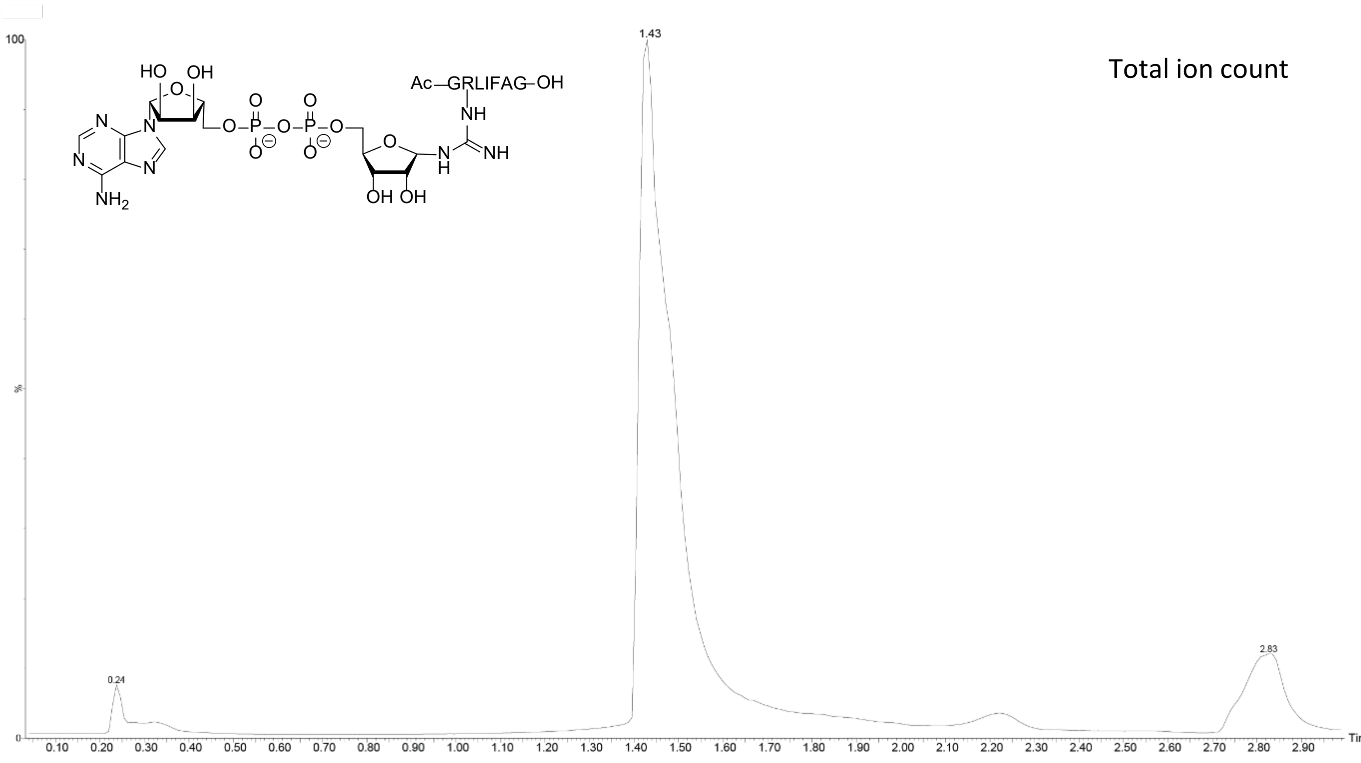

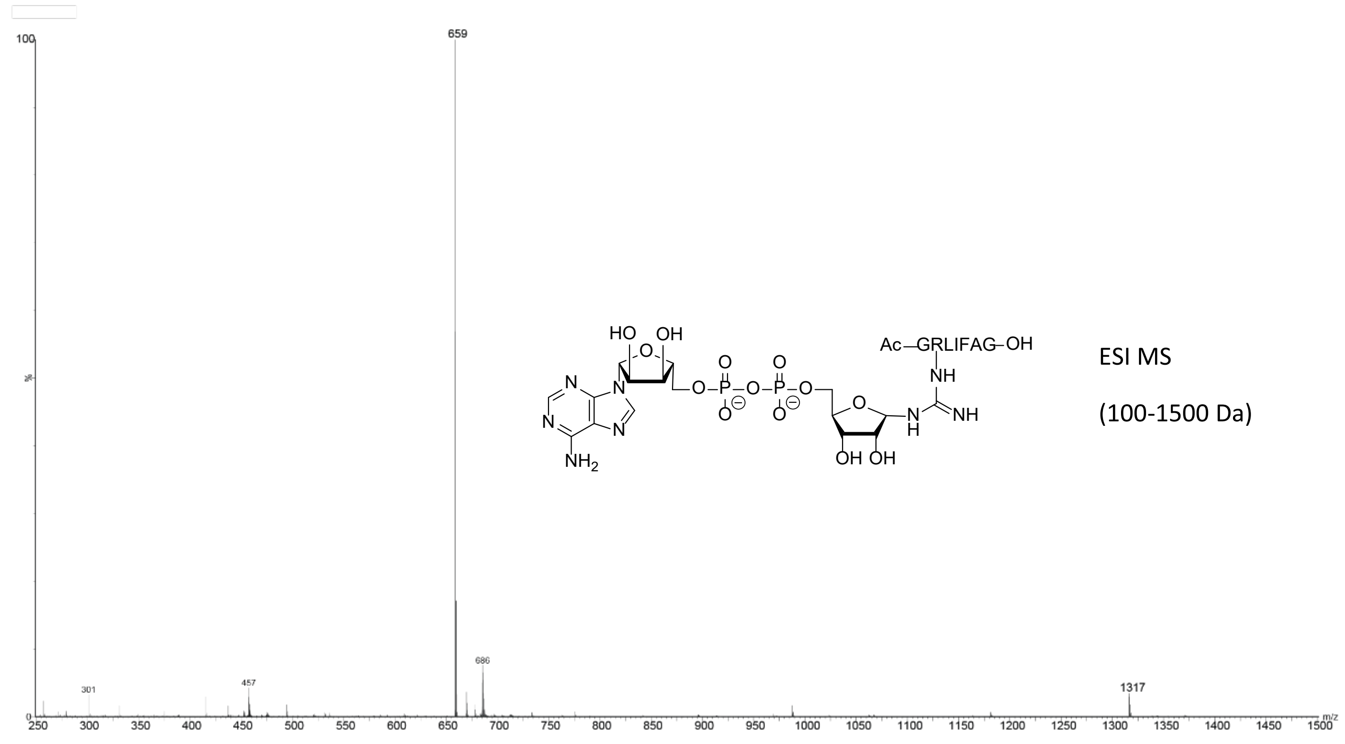

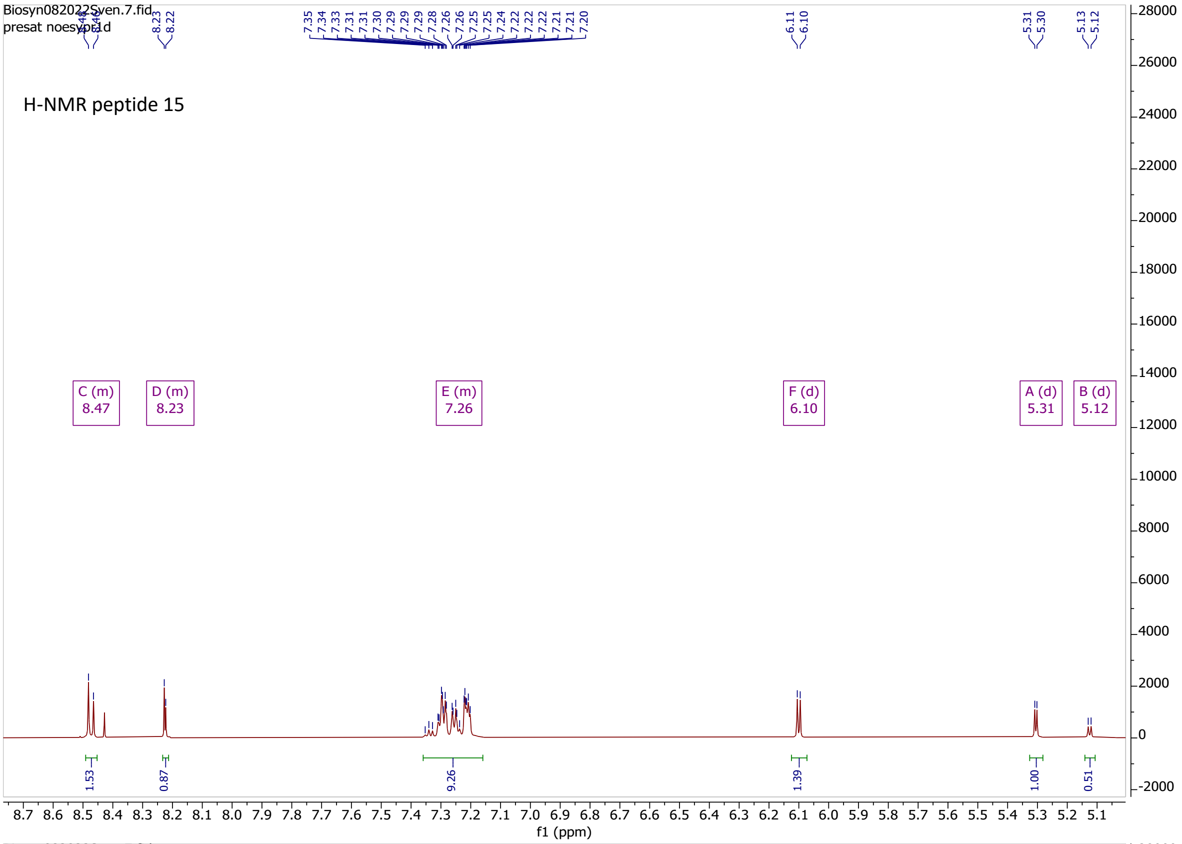
Biosyn082022Sven.7.fid
presat noesypr1d
H-NMR peptide 15
C (m) 8.47
D (m) 8.23
E (m) 7.26
F (d) 6.10
A (d) 5.31
B (d) 5.12
1.53
0.87
9.26
1.39
1.00
0.51
f1 (ppm)

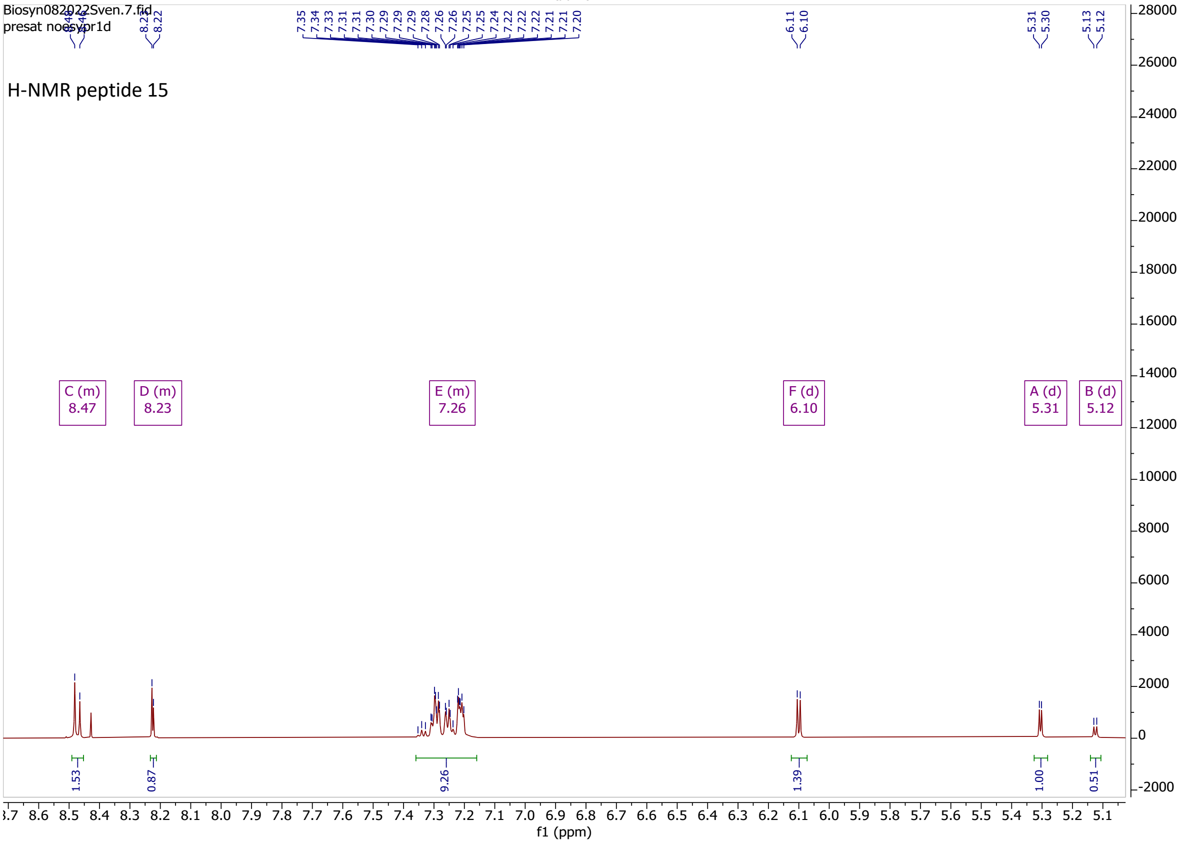
Biosyn082022Sven.7.fid
presat noesypr1d
H-NMR peptide 15
C (m) 8.47
D (m) 8.23
E (m) 7.26
F (d) 6.10
A (d) 5.31
B (d) 5.12
1.53
0.87
9.26
1.39
1.00
0.51
f1 (ppm)

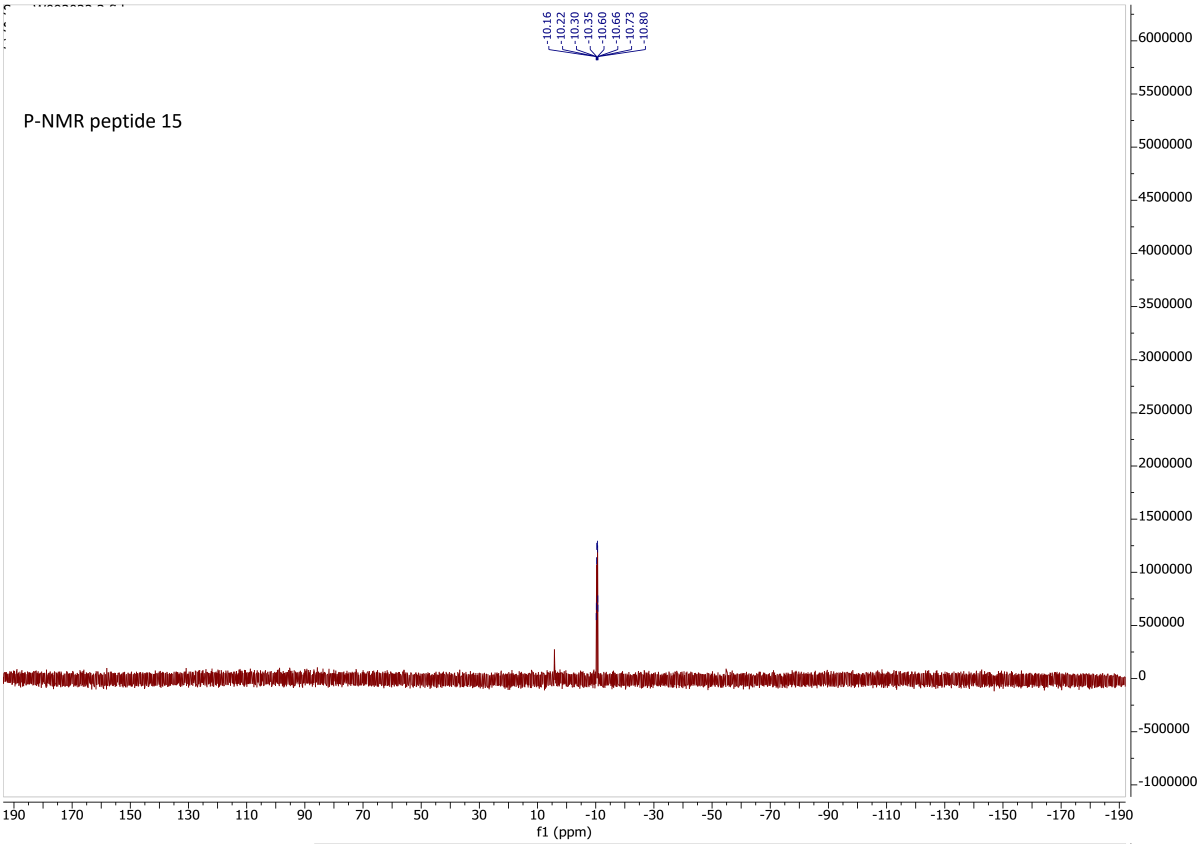
-10.16
-10.22
-10.30
-10.35
-10.60
-10.66
-10.73
-10.80
P-NMR peptide 15
f1 (ppm)

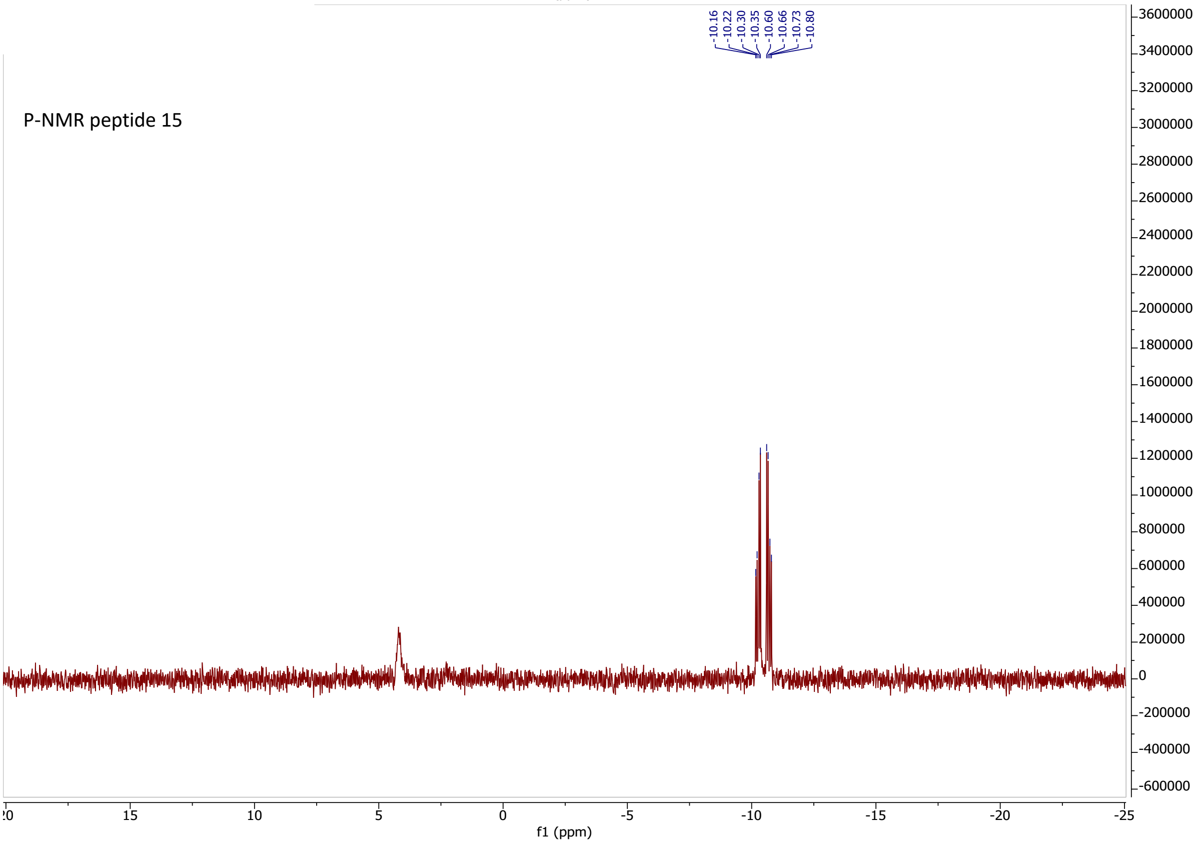
-10.16
-10.22
-10.30
-10.35
-10.60
-10.66
-10.73
-10.80
P-NMR peptide 15
f1 (ppm)

HRMS of Arg-ADPribosylated peptide (16)

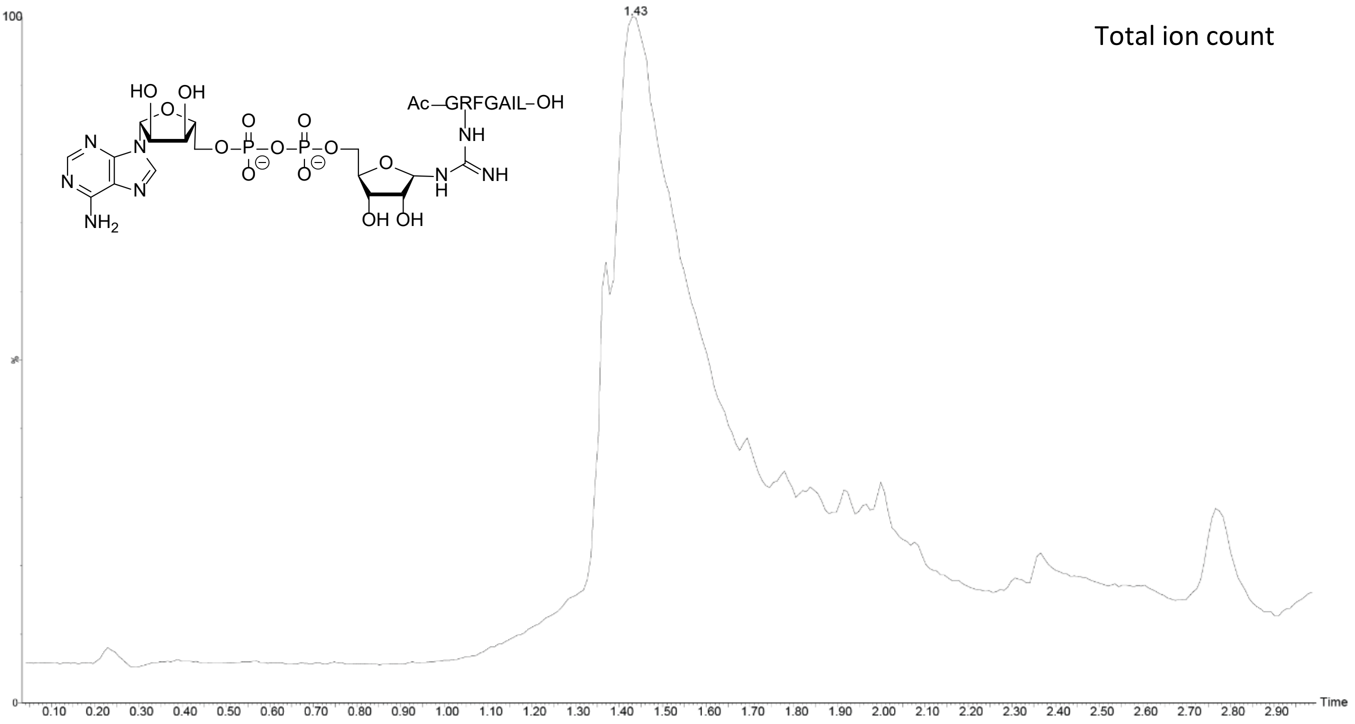

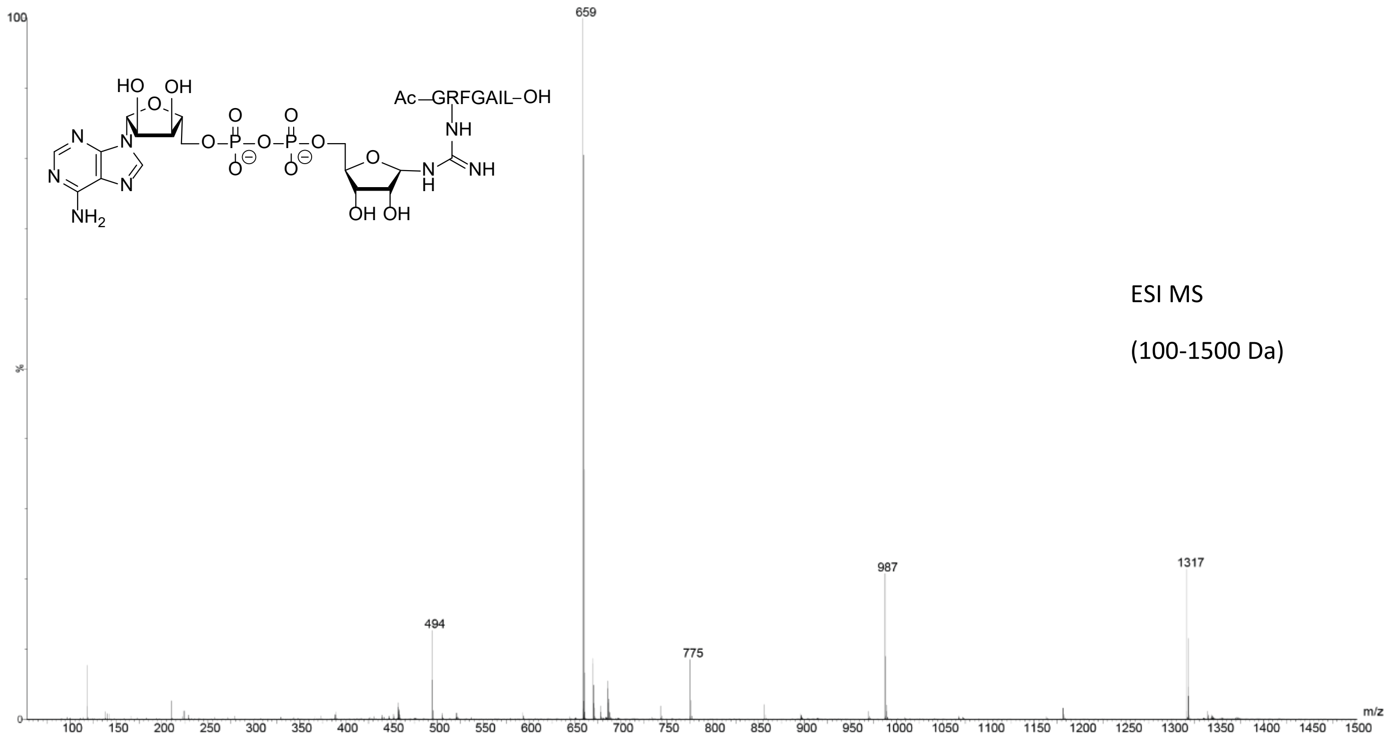

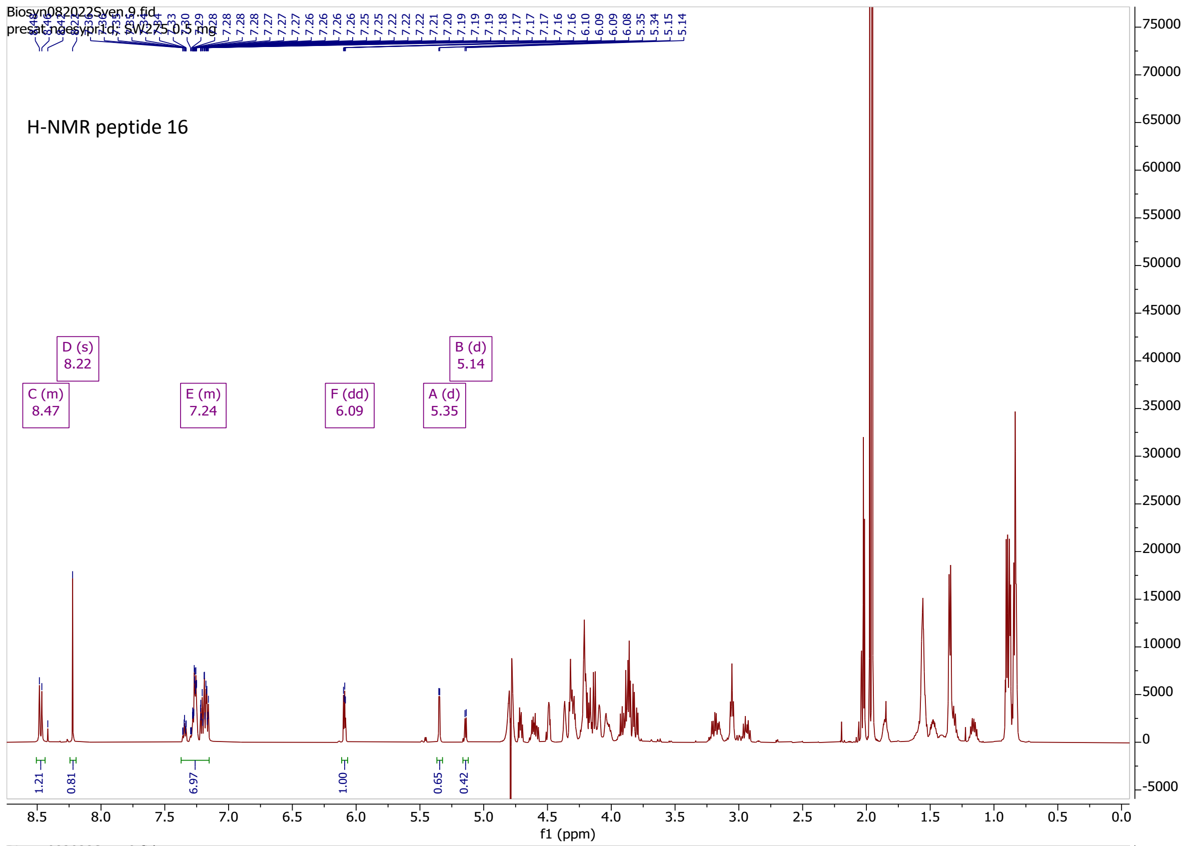
H-NMR peptide 16
C (m) 8.47
D (s) 8.22
E (m) 7.24
F (dd) 6.09
A (d) 5.35
B (d) 5.14
f1 (ppm)

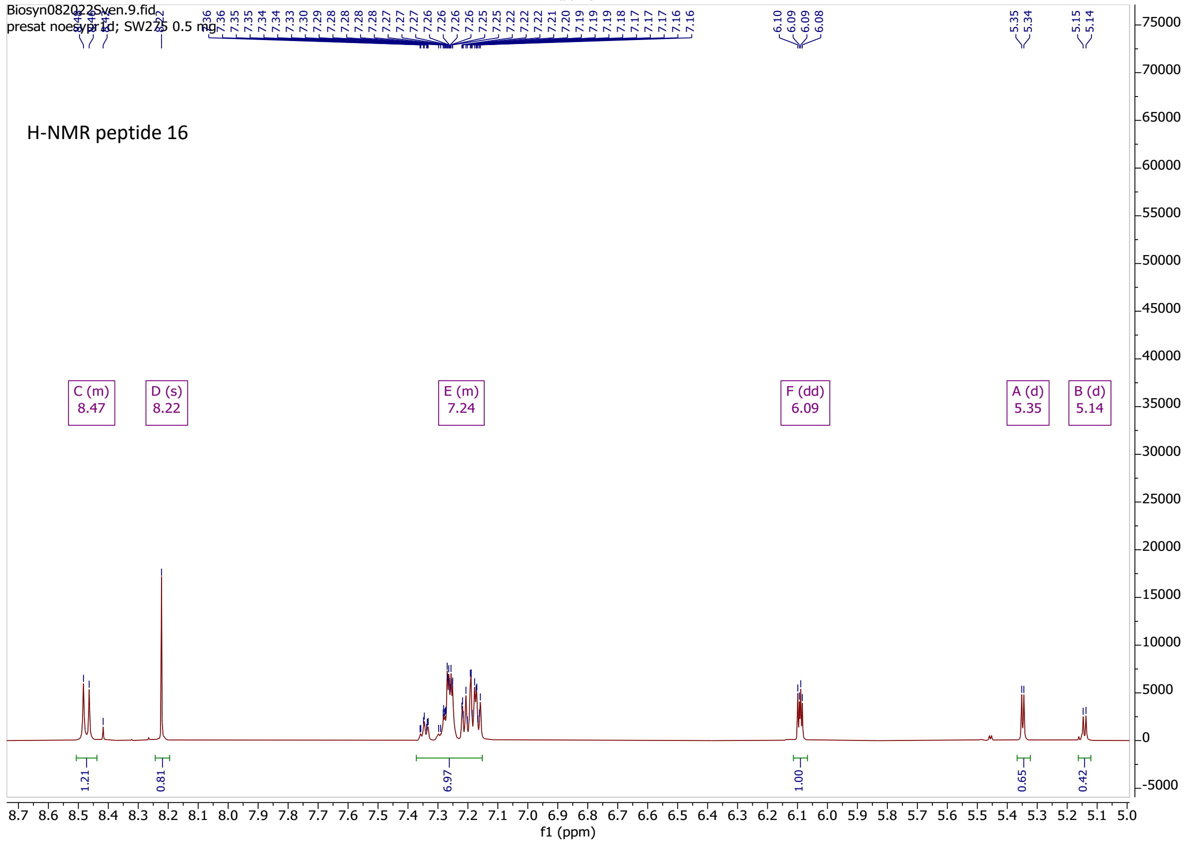
H-NMR peptide 16
C (m) 8.47
D (s) 8.22
E (m) 7.24
F (dd) 6.09
A (d) 5.35
B (d) 5.14
f1 (ppm)

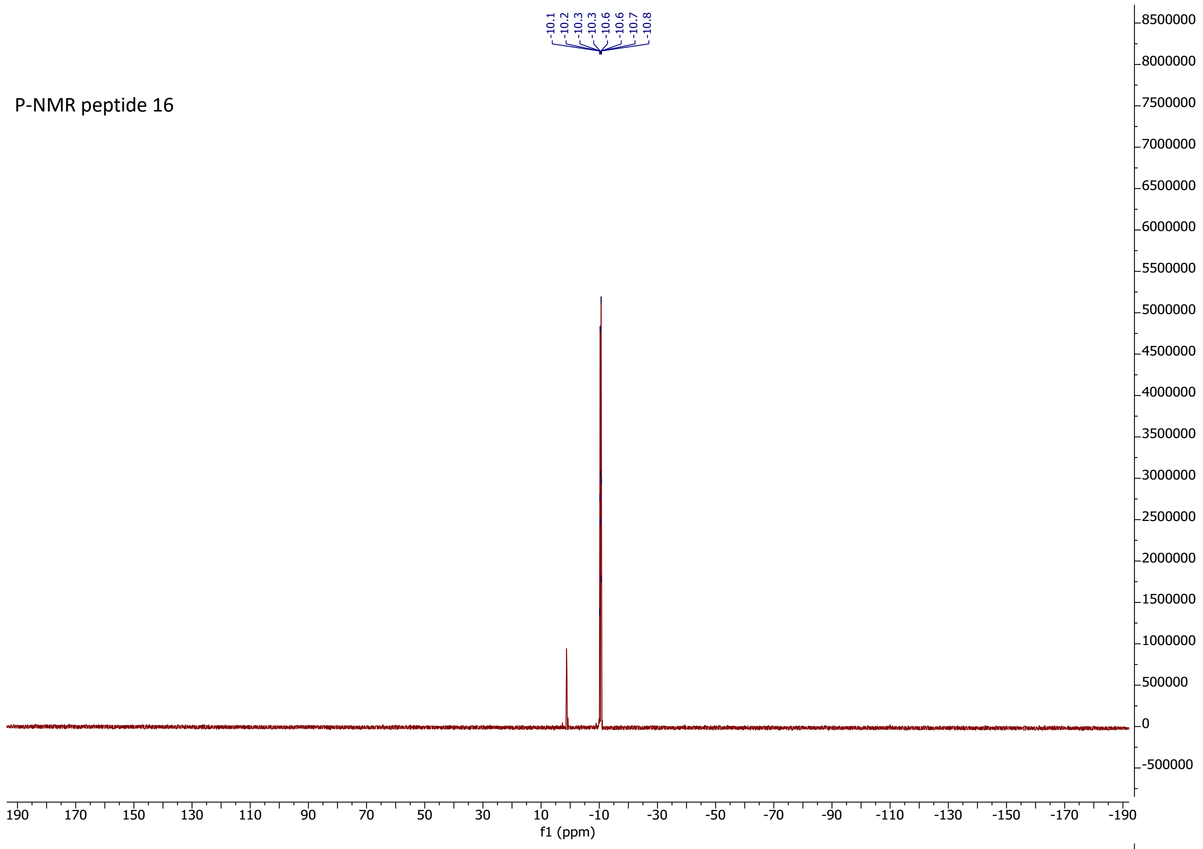
P-NMR peptide 16
-10.1
-10.2
-10.3
-10.3
-10.6
-10.6
-10.7
-10.8
f1 (ppm)

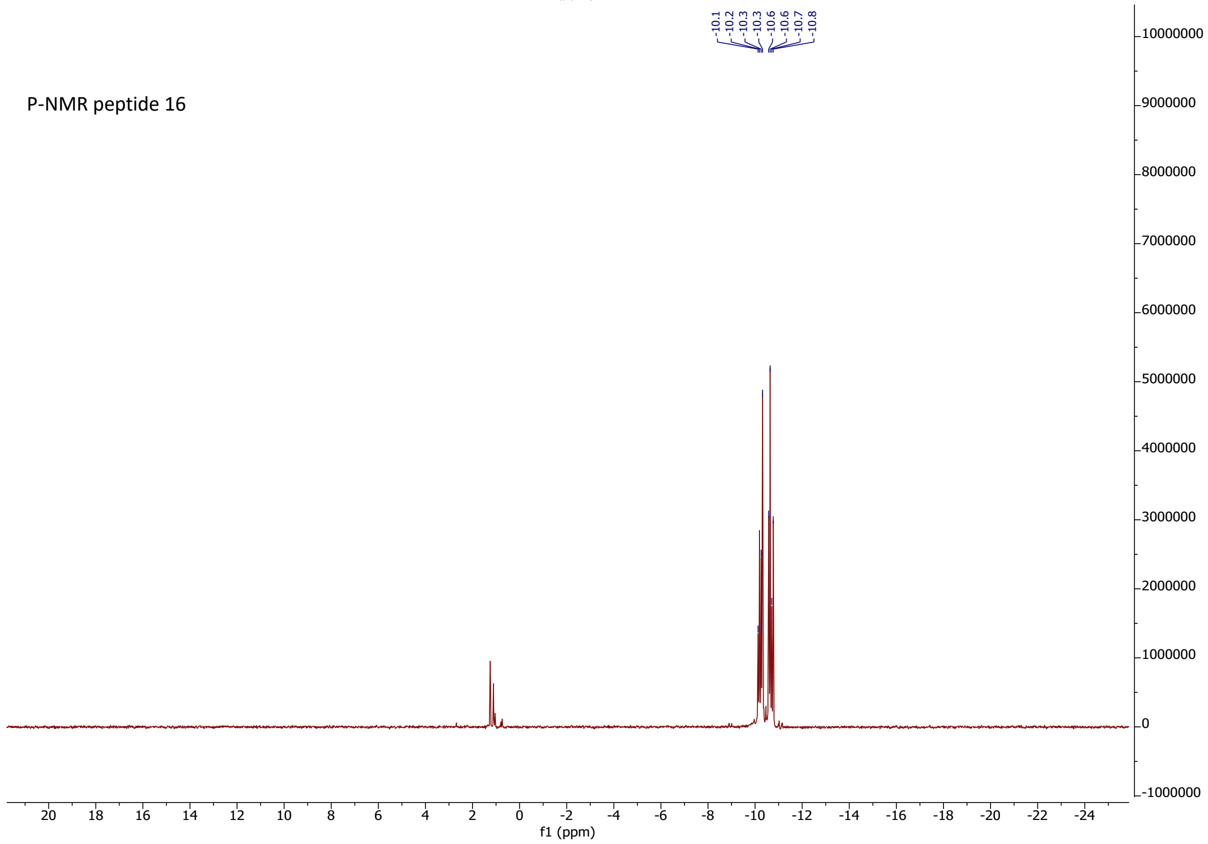
P-NMR peptide 16
-10.1
-10.2
-10.3
-10.3
-10.6
-10.6
-10.7
-10.8
f1 (ppm)

HRMS of Arg-ADPribosylated peptide (17)

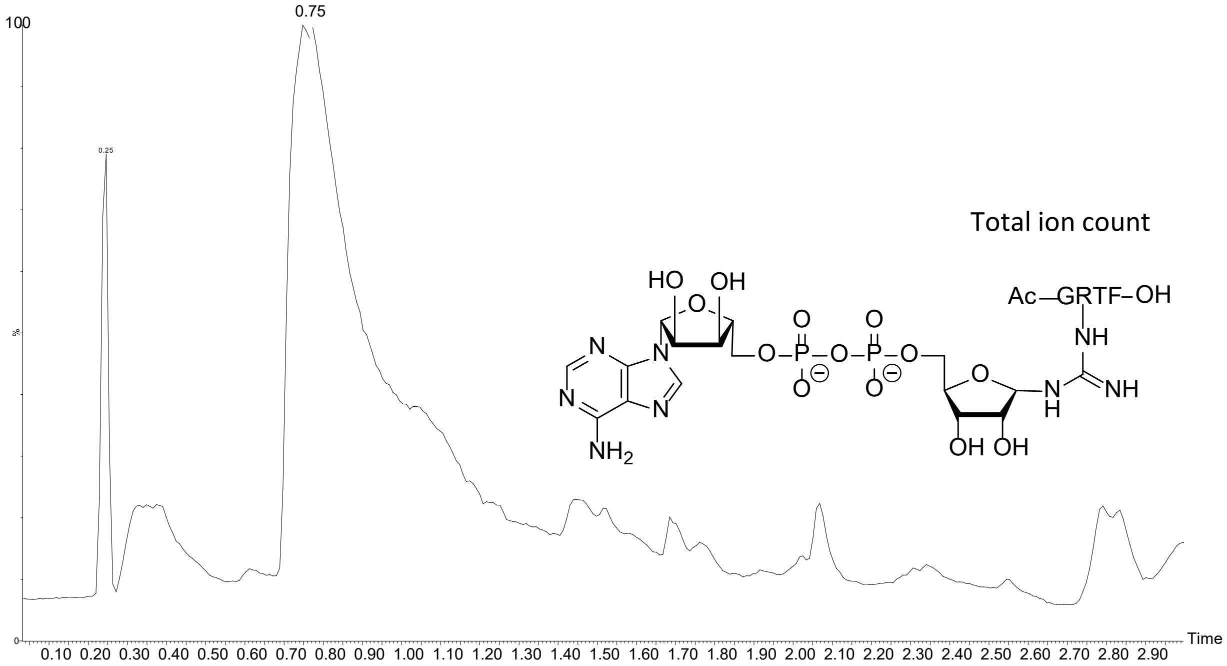

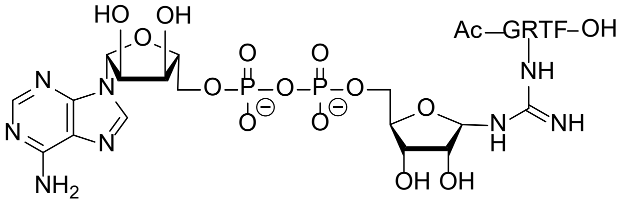

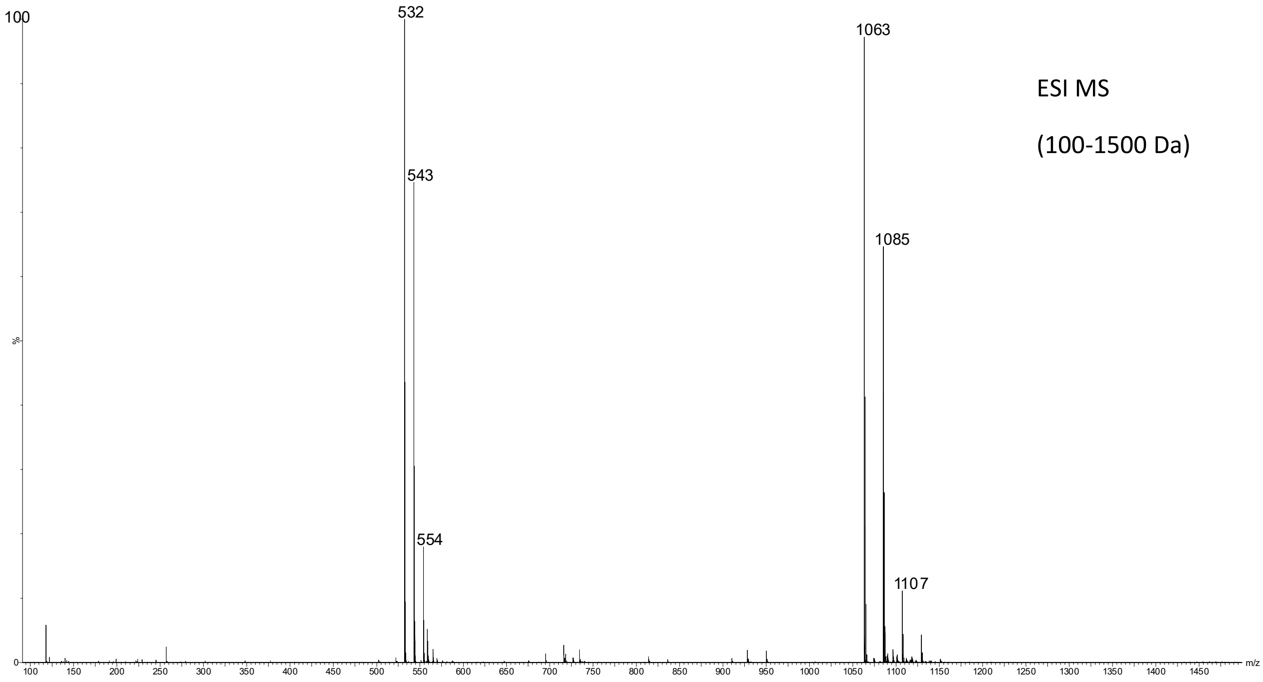

/

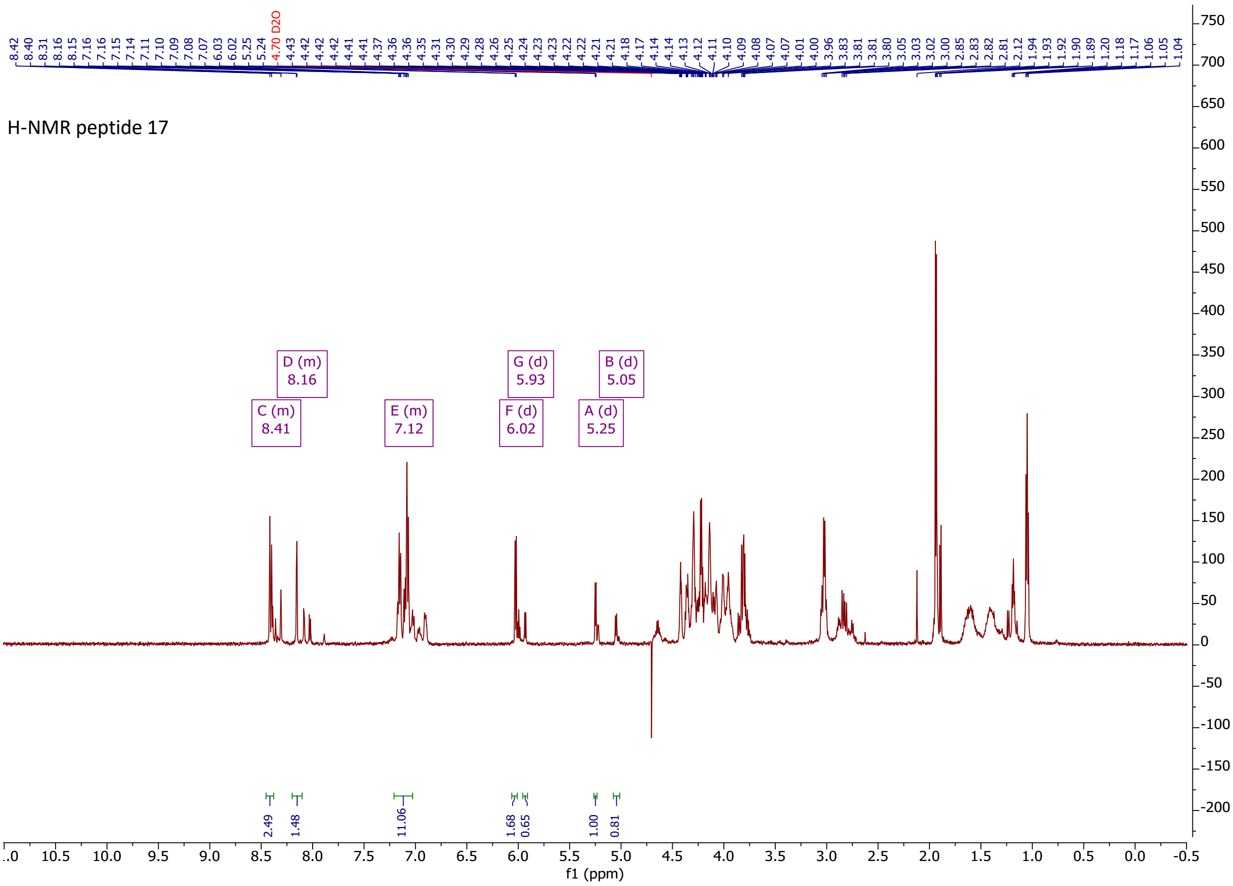
8.42 8.40 8.31 8.16 8.15 7.16 7.16 7.15 7.14 7.11 7.10 7.09 7.08 7.07 6.03 6.02 5.25 5.24 4.70 D2O 4.43 4.42 4.42 4.42 4.41 4.41 4.37 4.36 4.36 4.35 4.31 4.30 4.29 4.28 4.26 4.25 4.24 4.23 4.23 4.22 4.22 4.21 4.21 4.18 4.17 4.14 4.13 4.12 4.11 4.10 4.09 4.08 4.07 4.07 4.01 4.00 3.96 3.83 3.81 3.81 3.80 3.05 3.03 3.02 3.00 2.85 2.83 2.82 2.81 2.12 1.94 1.93 1.92 1.90 1.89 1.20 1.18 1.17 1.06 1.05 1.04
H-NMR peptide 17
D (m) 8.16
G (d) 5.93
B (d) 5.05
C (m) 8.41
E (m) 7.12
F (d) 6.02
A (d) 5.25
2.49
1.48
11.06
1.68
0.65
1.00
0.81
f1 (ppm)

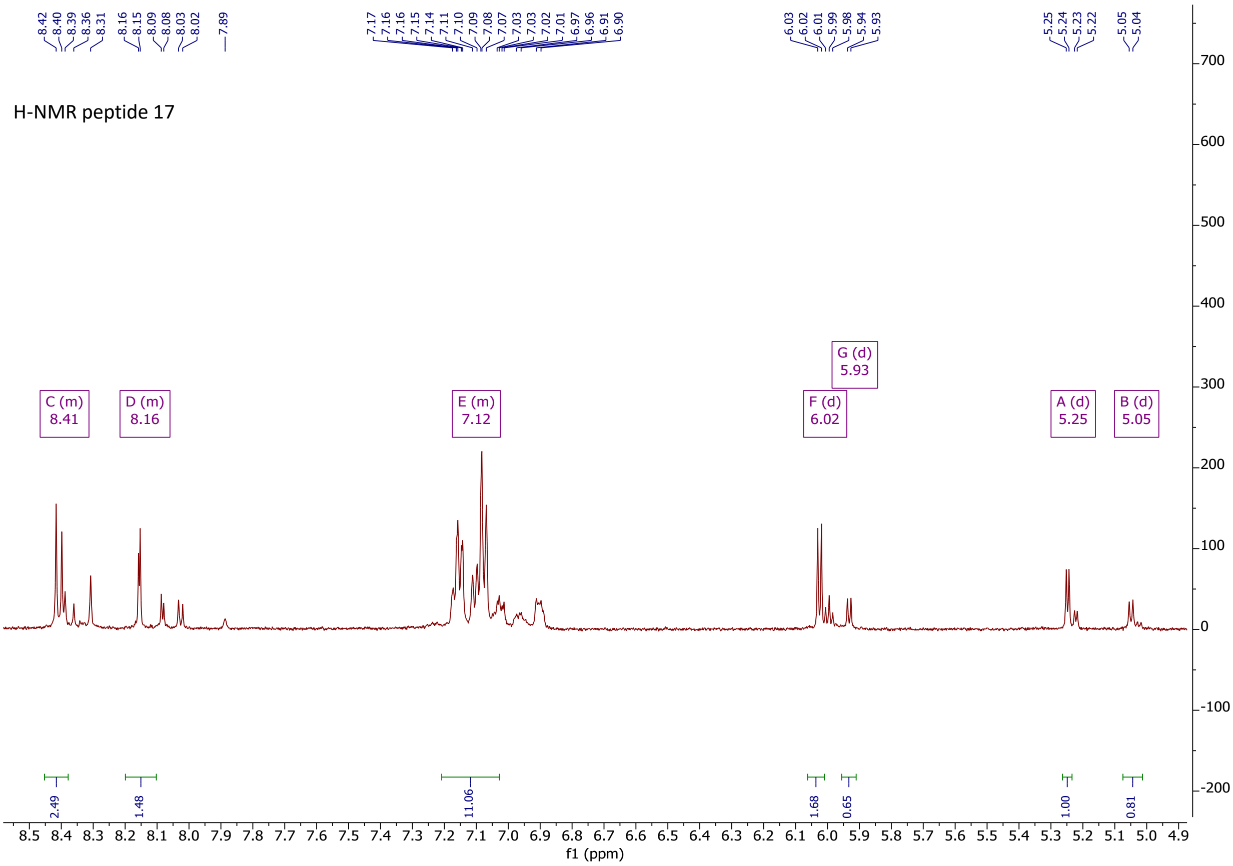
8.42 8.40 8.39 8.36 8.31 8.16 8.15 8.09 8.08 8.03 8.02 7.89
7.17 7.16 7.16 7.15 7.14 7.11 7.10 7.09 7.08 7.07 7.03 7.03 7.02 7.01 6.97 6.96 6.91 6.90
6.03 6.02 6.01 5.99 5.98 5.94 5.93
5.25 5.24 5.23 5.22 5.05 5.04
H-NMR peptide 17
G (d) 5.93
C (m) 8.41
D (m) 8.16
E (m) 7.12
F (d) 6.02
A (d) 5.25
B (d) 5.05
2.49
1.48
11.06
1.68
0.65
1.00
0.81
f1 (ppm)

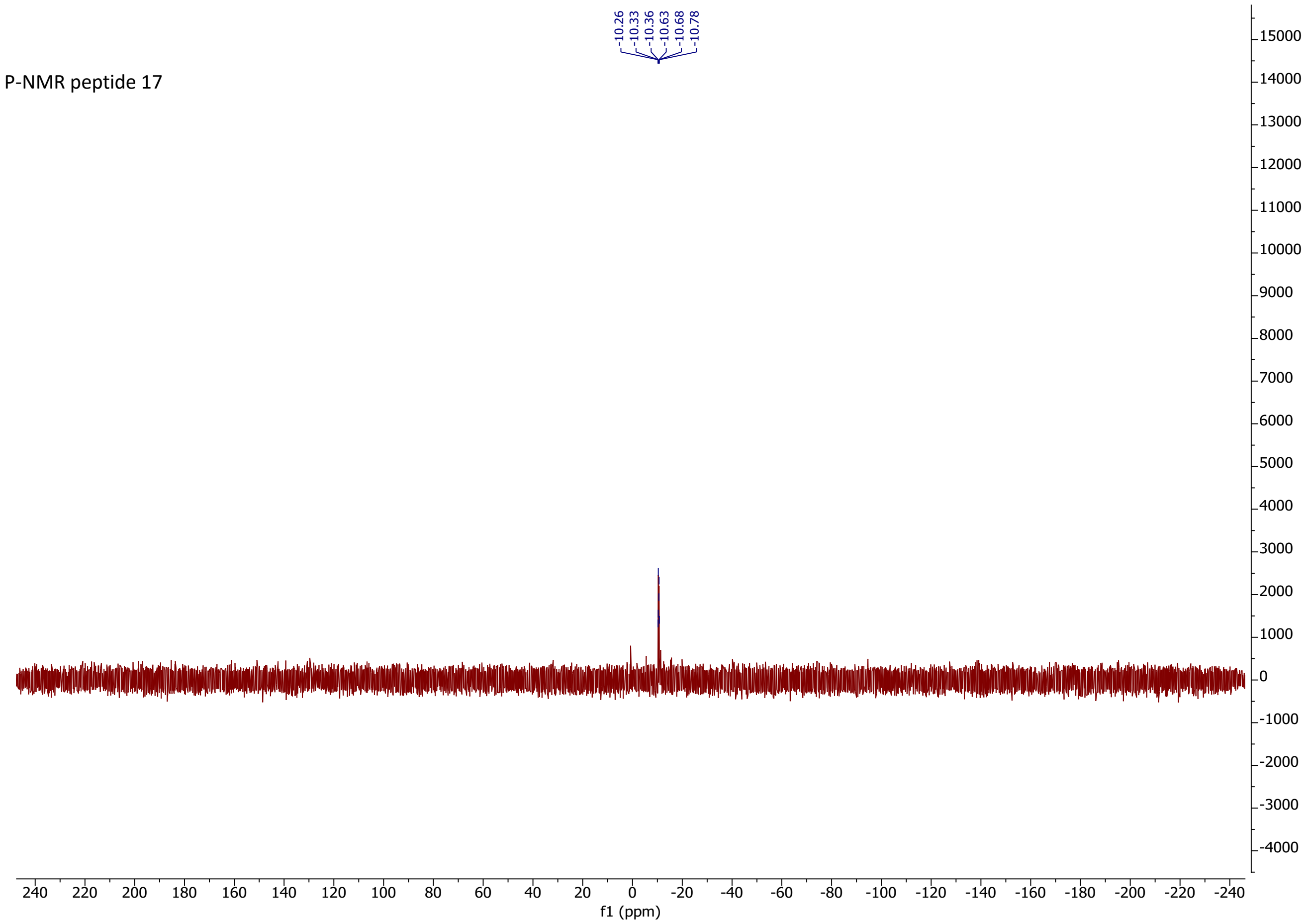
P-NMR peptide 17
-10.26
-10.33
-10.36
-10.63
-10.68
-10.78
f1 (ppm)

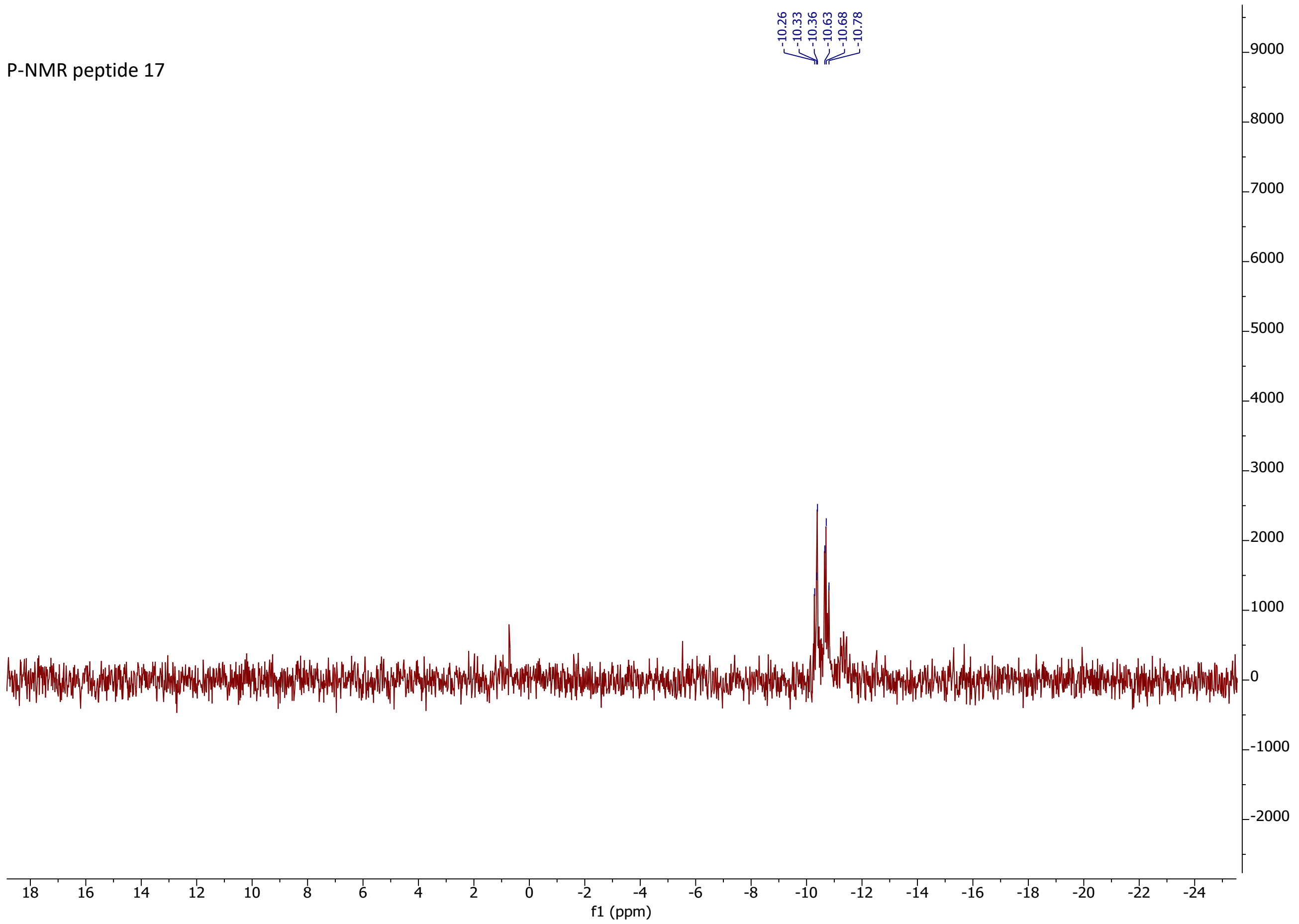
P-NMR peptide 17
-10.26
-10.33
-10.36
-10.63
-10.68
-10.78
f1 (ppm)
